# Chemical Composition Analysis of Highland Barley (*Hordeum vulgare* L.) with Different Modification Methods and Lipid Metabolism Mechanism Analysis of Highland Barley with Microwave Fluidization Modification

**DOI:** 10.3390/foods15081396

**Published:** 2026-04-17

**Authors:** Xiang Li, Kevin Shyong Wei Tan, Pengxiao Chen

**Affiliations:** 1 School of Food and Strategic Reserves, Henan University of Technology, Zhengzhou 450001, China; lixiang0625@haut.edu.cn; 2 Laboratory of Molecular and Cellular Parasitology, Healthy Longevity Translational Research Programme, Department of Microbiology and Immunology, Yong Loo Lin School of Medicine, National University of Singapore, 5 Science Drive 2, Singapore 117545, Singapore; mictank@nus.edu.sg

**Keywords:** highland barley, chemical composition, untargeted metabolomics, RNA-seq transcriptomics, lipid metabolism mechanism

## Abstract

In this study, the chemical composition of highland barley (HB), microwave fluidization HB (HB-1), extrusion and puffing HB (HB-2), and ultrafine pulverization HB (HB-3) were investigated based on untargeted metabolomics. In addition, RNA-seq transcriptomics, real-time polymerase chain reaction (qRT-PCR) and Western blot (WB) analysis were used to investigate the lipid metabolism mechanism of HB-1, induced by a high fat and cholesterol diet (HFCD). The results indicated that a total of 1292 metabolites were detected and classified into 78 distinct classes in the untargeted metabolomics analysis including fatty acyls, carboxylic acids and derivatives, glycerophospholipids, organooxygen compounds, prenol lipids, and so on. HB-1, HB-2, and HB-3 all increased the levels of amino acids and their derivatives, phenols, and carboxylic acid and its derivatives compared with HB. Furthermore, RNA-seq transcriptomic results indicated that HB-1 significantly modulated key genes of *Cyp2c38*, *Cyp2b13*, and *Cyp2b9* related to steroid hormone biosynthesis and *CD36*, *Plin4*, and *Fabp4* related to the PPAR signaling pathway, which played key roles in lipid metabolism. Moreover, qRT-PCR and WB results indicated that HB-1 obviously enhanced ADIPOQ expression level, while it reduced SCD-1, CD36, Fabp4, and SREBP-1c expression levels, suggesting that the alleviation of lipid metabolic dysregulation by HB-1 in hyperlipidemia mice might be mediated via participating in the PPARγ pathway. This study provided essential theoretical insights for the development and utilization of HB.

## 1. Introduction

Highland barley (HB, *Hordeum vulgare* L.) is an annual cereal crop belonging to the genus *Hordeum* within the family Gramineae. It is also commonly referred to as rice barley or naked barley. HB presents a nutritional advantage with its abundant protein, fiber, and vitamin, coupled with low fat and sugar. HB is rich in abundant bioactive ingredients including β-glucan, polysaccharides, dietary fibers, and phenols compounds, making it distinct from conventional grains [1]. Distinct physiological characteristics are observed within this species, which are categorized into two-rowed, four-rowed, and six-rowed varieties. Specifically, the four-rowed variant of HB is found to be predominantly cultivated in the Qinghai province, while the six-rowed variety is primarily produced throughout the Tibetan region. More than 80% of the output is remarkably attributed to these HB types [2]. Previous studies had indicated that HB exerted beneficial effects of preventing hyperlipidemia, improving lipid metabolism disorders, and attenuating liver inflammation [3]. In addition, multitude secondary metabolites are cumulatively accumulated in the grains of HB due to its cultivation in high-altitude terrains with intense seasonal aridity, ultraviolet solid radiation, and low oxygen levels. These compounds play a key role in providing HB with its health-promoting benefits [4]. Due to its unique nutritional value and biologically active components, HB has received considerable attention in the food industry. This focus indicates its potential in driving the development of functional food products [5].

In HB, the outer integument and cell wall architecture are largely composed of a diverse array of non-starch polysaccharides, such as arabinoxylan, pectin, and various hemicelluloses [6]. The presence of these chemical constituents and structural barriers protects HB from damage. Nevertheless, they limit grinding efficiency, processing performance, and the full liberation of bioactive compounds. Consequently, innovative food processing technologies designed to disrupt the peripheral integument and cell wall architecture have been widely adopted. These advancements are pivotal in the production of ready-to-eat barley-based products with tailored nutritional profiles within the whole-grain industry [7]. The modified technologies of HB mainly include microwave fluidization, heat fluidization, extrusion and puffing, ultrafine pulverization/grinding, and so on. A previous study had proved that ultrafine grinding significantly enhanced the physicochemical and antioxidant properties of hull-less barley (Qingke) dietary fibers [8]. Their findings revealed that this mechanical treatment elevated the concentrations of dietary fibers and β-glucan, which were critical for cholesterol reduction and glycemic regulation, thereby improving the overall nutritional profile of HB-derived components [9]. Thermal processing, particularly heat fluidization, has been shown to liberate free phenolics and improve β-glucan solubility in HB, albeit at the cost of reduced bound phenolic content [10]. However, previous studies on HB processing have mainly focused on isolated chemical composition, such as β-glucan, whereas systematic comparisons of the overall chemical composition changes induced by different modification methods are very few. Furthermore, related studies integrating the chemical composition of HB with its regulatory effects on lipid metabolism remain limited.

Hyperlipidemia is closely associated with oxidative stress and lipid dysfunction, which is a metabolic disorder characterized by significant mortality and morbidity [11]. The onset and development of the increase in metabolic syndromes including insulin resistance, Alzheimer’s disease, hypertension, and obesity are closely related to hyperlipidemia [12]. A high fat and cholesterol diet (HFCD) has been widely acknowledged as a major risk factor for hyperlipidemia, particularly for diets rich in saturated and trans fats [13]. Excessive fat and cholesterol intake could cause energy consumption exceeding the body’s requirements, and excess energy is stored as fat in the body, thereby increasing risks of hyperlipidemia, non-alcoholic steatohepatitis (NASH), non-alcoholic fatty liver disease (NAFLD), fat accumulation, and lipid metabolism disorder [14]. Lipid metabolism disorders, which are caused by long term HFCD intake, are marked by elevated levels of serum low-density lipoprotein cholesterol (LDL-C), total cholesterol (TC), triglyceride (TG), and more lipid droplets accumulation [15]. With the growing concern regarding hyperlipidemia caused by HFCD and the severe side effects of drugs for anti-hyperlipidemia and improving lipid metabolism, dietary supplementation with some bioactive food or their modified products is very necessary for the prevention of lipid metabolism disorders in hyperlipidemia patients [16]. Increasing studies have proved that dietary HB provided a promising and important treatment for preventing and reversing lipid metabolism disorders and liver injury [17]. Our previous study had proved that HB-1 exhibits the best effect of improving lipid metabolism. Modified HB (MHB), especially HB-1, significantly improved lipid indexes, liver function, liver injury, and blood glucose indexes related to hyperlipidemia compared with the high fat and cholesterol diet (HFCD). In addition, MHB, especially HB-1, recovered the disorder in gut microbiota by increasing *Bacteroidetes*/*Firmicutes* ratio, and *Lactobacillus* and *Allobaculum* abundances, while decreasing *Proteobacteria* abundance related to lipid metabolism bacteria. MHB, especially HB-1, reversed the decrease in short chain fatty acids levels induced by HFCD [18]. However, the related molecular mechanisms by which MHB affects lipid metabolism have not yet been fully clarified.

The liver serves as the primary regulator of lipid homeostasis, coordinating an integrated network of metabolic pathways, including de novo lipogenesis, fatty acids (FAs) uptake, redistribution of FAs to other organs, and the utilization of energy sources, which makes it the core organ responsible for energy and lipid metabolism [19]. These processes are strictly controlled by complex cooperation of transcription factors, nuclear receptors, and hormones, maintaining regulated and stable hepatic lipid homeostasis [20]. Liver lipid metabolism disorders and NAFLD are characterized by abnormal hepatic lipid deposition, especially triglycerides (TG), FAs, and various cholesterols, mostly induced by the imbalance between lipid intake and removal caused by HFCD [21]. Fatty Acid Binding Protein 4 (FABP4), Cluster of Differentiation 36 (CD36), Sterol Regulatory Element-Binding Protein-1c (SREBP-1c) and Stearoyl-CoA Desaturase-1 (SCD-1) were activated, and adiponectin (ADIPOQ) was inhibited by FAs. This behavior could lead to the transfer and differentiation of FAs in the liver, the production of fat droplets, TG, fat accumulation, and the promotion of NAFLD/NASH and lipid metabolism disorders [22]. ADIPOQ could increase FAs oxidation and reduce lipid accumulation, preventing fatty degeneration of the liver, including NAFLD. It also could reduce inflammatory responses by enhancing anti-inflammatory factors’ expressions and reducing pro-inflammatory factors’ expressions [23]. Abnormal expression of FABP4 is associated with hepatic fat deposition (NAFLD). However, the intricate molecular mechanism and related cellular behavior that occurred during the onset and progression of lipid metabolism disorders and hepatic steatosis were not entirely clarified.

In this study, microwave fluidization, extrusion and puffing, and ultrafine pulverization were used to modify HB, and an untargeted metabolomics analysis based on liquid chromatography-mass spectrometry (LC-MS) was employed to explore the metabolic profiles and identified differences in the chemical composition of these three modification methods. Combined with RNA-seq transcriptomics analysis, real-time polymerase chain reaction (qRT-PCR) assessment and Western blot (WB) analysis were used to explore the detailed molecular mechanism. This study aimed to characterize the impacts of different processing methods on the bioactive components of HB and to investigate the underlying mechanisms contributing to the hyperlipidemia-improving effects of MHB, thereby delivering key insights into its developmental prospects and therapeutic exploitation.

## 2. Materials and Methods

### 2.1. Materials and Reagents

All 4 samples of HB, including HB, microwave fluidized HB (HB-1), extruded and puffed HB (HB-2), and ultrafine pulverized HB (HB-3) were supplied by Qinghai Huashi Technology Investment Management Co., Ltd. (Qinghai, China). Methanol and acetonitrile were purchased from Thermo Fisher Scientific Inc. (Waltham, MA, USA). 2-Chloro-L-phenylalanine was purchased from Aladdin Biochemical Technology Co., Ltd. (Shanghai, China). Formic acid was purchased from Tokyo Chemical Industry Co., Ltd. (Tokyo, Japan). Ammonium formate was purchased from Sigma-Aldrich (Saint Louis, MO, USA). NEBNext Ultra II RNA Library Prep Kit for Illumina was purchased from New England Biolabs (Ipswich, MA, USA). Trizol reagent was purchased from Invitrogen (Carlsbad, CA, USA). DEPC water, dNTP, TBE, and 6×Loading buffer were purchased from Shanghai Sunny Biotechnology Co., Ltd. (Shanghai, China). Taq enzyme, random primers, RNase inhibitor, and MMLV were purchased from TaKaRa Biotechnology (Shiga, Japan). AceQ^®^ qPCR SYBR^®^ Green Master was purchased from Vazyme Biotechnology (Nanjing, China). RIPA Lysis Buffer was supplied by Meilun Biotechnology Co., Ltd. (Dalian, China). MOPS Running Buffer liquid (20×) was supplied by Genscript Biotechnology Co., Ltd. (Nanjing, China). Analytical grade chemicals were used for all other reagents in this study.

### 2.2. Sample Preparation

HB (Kunlun 14, harvested from the Tianyoude organic highland barley cultivation base) was supplied by Qinghai Huashi Technology Investment Management Co., Ltd. (Qinghai Engineering Research Institute for Comprehensive Utilization of Highland Barley Resources, Xining, Qinghai, China).

Process of microwave fluidized HB: Raw HB grains were cleaned, dehulled, and screened, followed by microwave-fluidized treatment using a DXY-20 kW tunnel-type microwave system. The samples were spread at a thickness of approximately 1 cm and processed at 110 °C with a conveyor frequency of 5 Hz. After treatment, the microwave-fluidized HB was ground using a high-speed multifunctional grinder and passed through a 120-mesh sieve to obtain the final microwave-fluidized HB powder (HB-1).

Process of extruded and puffed HB: The moisture content of raw HB powder was first determined in accordance with GB/T 21305-2007 [24]. The moisture level was subsequently adjusted to 16% by adding a calculated volume of distilled water; the mixture was homogenized using an electric mixer for 10–15 min and sealed in polyethylene bags overnight to ensure uniform moisture diffusion. The extruder was preheated for 30 min prior to operation. The extrusion process was performed across three distinct thermal zones maintained at 50 °C (Zone I), 120 °C (Zone II), and 150 °C (Zone III). Post-extrusion, the expanded samples were pulverized using a pulsed grinding technique (20 s intervals every 5 min) to maintain the powder temperature below 45 °C, thereby preventing undesired starch gelatinization. The final extruded and puffed HB was passed through a 120-mesh sieve and stored for subsequent analysis (HB-2).

Process of ultrafine pulverized HB: Raw HB grains were cleaned to remove impurities and stones, followed by slight dehulling and pre-grinding. Subsequently, the material was subjected to dry ultrafine grinding, with the processing temperature maintained below 35 °C and the particle size controlled to pass through a 120-mesh sieve. The obtained powder was further classified by airflow sieving, packaged, and collected as the final ultrafine HB powder (HB-3).

### 2.3. Untargeted Metabolomics Determination

HB, HB-1, HB-2, and HB-3 (*n* = 3) were accurately weighed (100 mg) and transferred into 2 mL centrifuge tubes. Following the addition of 400 µL methanol, the samples were vigorously vortexed and then equilibrated by centrifugation (12,000 rpm, 4 °C) for 10 min. Following centrifugation, the resultant supernatant was collected into new 2 mL tubes and concentrated to dryness. Subsequently, the samples were re-dissolved using 150 µL of an 80% aqueous methanol solution containing 4 ppm 2-chloro-l-phenylalanine. The obtained supernatant was collected, filtered through a 0.22 μm membrane, and the filtrate was added into a detection vial for LC-MS analysis.

The analysis was conducted using a Thermo Vanquish ultra-high-performance liquid chromatography system (Thermo Fisher Scientific, Waltham, MA, USA) with an ACQUITY UPLC^®^ HSS T3 column (2.1 × 100 mm, 1.8 µm) (Waters, Milford, MA, USA). The flow rate was set to 0.3 mL/min, the column temperature was maintained at 40 °C, and the injection volume was 2 µL. LC-ESI (+)-MS analysis was performed with the mobile phase consisting of 0.1% formic acid in acetonitrile (B2) and 0.1% formic acid in water (A2). The gradient elution program was as follows: from 0 to 1 min, 10% B2; from 1 to 5 min, 10% to 98% B2; from 5 to 6.5 min, 98% B2; from 6.5 to 6.6 min, 98% to 10% B2; and from 6.6 to 8 min, 10% B2. LC-ESI (−)-MS analysis was performed with the mobile phase consisting of acetonitrile (B3) and 5 mM ammonium formate in water (A3). The gradient elution program was as follows: from 0 to 1 min, 10% B3; from 1 to 5 min, 10% to 98% B3; from 5 to 6.5 min, 98% B3; from 6.5 to 6.6 min, 98% to 10% B3; and from 6.6 to 8 min, 10% B3.

Mass spectrometric detection was conducted on a Thermo Q Exactive Focus instrument (Thermo Fisher Scientific, Waltham, MA, USA) equipped with an electrospray ionization (ESI) source. Acquisition of MS1 and MS/MS data was conducted simultaneously in Full MS-ddMS2 mode (data-dependent MS/MS). Electrospray ionization (LC-ESI) voltages were configured at +3.50 kV for LC-ESI (+)-MS and −2.50 kV for LC-ESI (−)-MS. Sheath and auxiliary gas flow rates were configured to 40 and 10 arb, respectively. A capillary temperature of 325 °C was employed, with full-scan MS data being acquired at a resolution of 70,000. The *m*/*z* range for the first-stage scan was 100–1000. HCD was used for second-stage fragmentation with a collision energy of 30 eV, and the second-stage resolution was set to 17,500. The first three ions in the collected signal were fragmented, and dynamic exclusion was applied to remove unnecessary MS/MS data.

The raw data files (.raw format) were imported into the commercial software Compound Discoverer™ 3.3 (version 3.3.2.31, Thermo Fisher Scientific, Waltham, MA, USA) for further processing. Peak extraction, alignment, and correction were performed based on the software’s new peak detection and peak quality scoring algorithms. Interference from background noise and low-quality peaks was significantly reduced by unique peak rating calculations and filtering processes. Peaks that were not detected in more than 50% of the QC samples were filtered out. Missing values for undetected peaks were imputed using the software’s Fill Gaps algorithm, followed by normalization based on the sum of total peak areas. Metabolites identification was performed based on a self-built library, the mzCloud online database (https://www.mzcloud.org/, accessed on 12 April 2026), and several external repositories including LIPID MAPS (https://www.lipidmaps.org/, accessed on 12 April 2026), HMDB (https://hmdb.ca/, accessed on 12 April 2026), MoNA (https://mona.fiehnlab.ucdavis.edu/, accessed on 12 April 2026), and the NIST 2020 MS/MS spectral library. MS1 mass tolerance was set to 15 ppm, and MS2 match factor threshold was established at 50. Data analysis involved univariate statistical analysis, multivariate statistical analysis, screening of differential metabolites, correlation analysis of differential metabolites, and KEGG pathway enrichment analysis.

### 2.4. Animal Arrangement

Specific pathogen-free (SPF) male ICR mice, aged 6 weeks with an initial body weight of 35.0 ± 3.0 g, were supplied by GemPharmatech Co., Ltd. (Nanjing, China). All mice (*n* = 10) were maintained in a SPF animal facility at the Experimental Animal Center of Jiangnan University and kept under a 12 h light/dark cycle at a controlled temperature of 21–24 °C and relative humidity of 50–60%. Standard chow and double-distilled water were provided ad libitum. All experimental procedures were approved by the Experimental Animal Ethical Management and Use Committee of Jiangnan University (Reference No. JN.No20210615c1000930[194]).

A total of 60 mice were randomly assigned to 6 experimental groups (*n* = 10). The specific group allocations and treatments were as follows:

(1) Normal control diet (NCD): fed the standard AIN-93M diet.
(2) High-fat/cholesterol diet (HFCD): fed the XT108C diet.
(3) HFCD + HB: fed the XT108C diet supplemented with HB.
(4) HFCD + HB-1: fed the XT108C diet supplemented with HB-1.
(5) HFCD + HB-2: fed the XT108C diet supplemented with HB-2.
(6) HFCD + HB-3: fed the XT108C diet supplemented with HB-3.

### 2.5. Diet Treatments

All experimental diets (XT108C alone or supplemented with HB, HB-1, HB-2, and HB-3) were manufactured to ensure nutritional consistency. All dietary formulations were prepared and provided by Jiangsu Synergy Pharmaceutical Bioengineering Co., Ltd. (Nanjing, China). The detailed compositions of mice feed are shown in Table S1.

### 2.6. Sample Collection

Following a one-week acclimation period, the body weight (BW), body weight gain (BWG), and feed intake (FI) of the 6 experimental groups were monitored and documented weekly throughout the 10-week experimental duration. On the final day of the animal experiment, the mice were fasted for 12 h. All mice were humanely euthanized via isoflurane inhalation at the end of the animal experiment. The blood, liver, kidneys, spleen, pancreas, brown fat, perirenal fat, subcutaneous fat, epididymis fat, cecal content, and colon content were collected from this same cohort and kept for further analysis.

### 2.7. Transcriptomics Analysis

Our previous study had proved that HB-1 exhibits the best effect of improving lipid metabolism—so HB-1 was chosen to investigate the detailed mechanism of regulating lipid metabolism in HFCD-induced hyperlipidemia mice. RNA-sequencing transcriptomics analysis was used to investigate the related differential genes and signaling pathways between HFCD and HFCD + HB-1 (*n* = 3). Using Trizol^®^ reagent, total RNA was purified from hepatic tissues between HFCD and HFCD + HB-1 (*n* = 3) according to the manufacturer’s instructions. Subsequently, any residual genomic DNA was removed by treatment with DNase I. The integrity and concentration of the extracted RNA were assessed using an Agilent 2100 Bioanalyzer and quantified via a NanoDrop 2000 spectrophotometer (Thermo Fisher Scientific, Waltham, MA, USA), respectively. For each sample, 3 μg of total RNA was employed for the construction of sequencing libraries. Total RNA samples were processed to extract mRNA via poly-T oligo-attached magnetic beads for the generation of sequencing libraries. mRNA molecules were subjected to fragmentation at high temperatures using divalent cations in an Illumina proprietary buffer. First-strand cDNA was generated via SuperScript II and random hexamers based on the fragmented mRNA templates. Second-strand cDNA synthesis was facilitated by the coordinated action of RNase H and DNA Polymerase I. cDNA fragments underwent end repair, with residual overhangs being repaired to blunt ends through exonuclease and polymerase-mediated reactions, followed by enzyme inactivation or purification to remove the proteins. After adenylation of the 3′ ends of the DNA fragments, Illumina PE adapter oligonucleotides were ligated to the samples to facilitate library construction. To select for cDNA fragments with the desired length of 400–500 bp, library fragments were purified and screened employing the AMPure XP system (Beckman Coulter, Brea, CA, USA). To selectively amplify the adapter-ligated fragments, the library was subjected to 15 cycles of PCR enrichment utilizing the Illumina PCR Primer Cocktail. Library validation was performed on an Agilent 2100 Bioanalyzer utilizing the High Sensitivity DNA assay (Agilent Technologies, Santa Clara, CA, USA). This step ensured the precise quantification and size distribution assessment of the purified products following the AMPure XP cleanup. High-throughput sequencing was performed on the NovaSeq 6000 system (Illumina, San Diego, CA, USA) using a paired-end 150 bp (PE150) strategy.

Quality control was performed by sequencing the samples on the platform to generate image files, which were subsequently processed by the platform software to produce the original FASTQ data (Raw Data). The sequencing output contained adapter sequences and low-quality reads, which were removed using fastp (v0.22.0) to obtain high-quality sequences (Clean Data) for downstream analyses. The reference genome and corresponding gene annotation files were retrieved from the official genome database. The high-quality filtered reads were aligned to the reference genome using HISAT2 (version 2.1.0) with default parameters. Only uniquely mapped reads were retained for subsequent transcript abundance estimation. The raw expression levels for each gene were quantified by calculating the read counts using HTSeq (version 0.9.1). To account for variations in gene length and sequencing depth, the expression data were normalized using the Fragments Per Kilobase of transcript per Million mapped reads (FPKM) method. Differentially expressed genes (DEGs) were identified using the DESeq package (version 1.38.3). The filtering criteria for significance were defined as an absolute log_2_ fold change (|log_2_FC| > 1) and a *p*-value < 0.05. Subsequently, bidirectional hierarchical clustering was performed on the identified DEGs using the Pheatmap package (v1.0.12) in R. The Euclidean distance method was used to calculate dissimilarity, and the Complete Linkage method was utilized to cluster the expression patterns of genes across different samples. All genes were mapped to terms in the Gene Ontology (GO) database, and the number of differentially expressed genes in each term was counted. GO enrichment analysis of the differential genes (all DEGs, up-regulated DEGs, and down-regulated DEGs) was performed using topGO (v2.50.0), with *p*-values calculated by the hypergeometric test; terms with *p* < 0.05 were considered significantly enriched. The main biological functions of differential genes were identified based on significantly enriched GO terms. KEGG pathway enrichment was conducted using ClusterProfiler (v4.6.0), and pathways with *p* < 0.05 were considered significant. In addition, Gene Set Enrichment Analysis (GSEA, v4.1.0) was performed for all genes, and pathway enrichment maps were generated. To identify previously unannotated genetic features, transcript assembly was performed using StringTie (version 2.2.1) based on the mapped reads and the existing reference genome. The resulting assembly was compared against known gene models to identify novel transcripts. These unannotated sequences were characterized by their unique splicing patterns and genomic coordinates relative to the reference annotation. Differential alternative splicing events were analyzed using rMATS (v4.0.1). The main types of splicing events examined included SE, RI, MXE, A5SS, and A3SS. Variant calling for SNPs and InDels were performed using VarScan (v2.3.9). Sites were filtered according to the following thresholds: base quality Q > 20, coverage > 8 reads, supporting reads > 2, and *p*-value < 0.01. The filtered variants were then subjected to further analysis. Prediction of transcription factors was conducted by querying PlantTFDB for plant sequences and AnimalTFDB for animal sequences. The family classification of each predicted transcription factor was determined according to the database. DEGs identified as transcription factors were then subjected to statistical analysis. Differences in exon usage were analyzed using the DEXSeq (v1.44.0) package. Exon usage differences were defined as variations in exon utilization between experimental conditions. Protein–protein interaction analysis of differentially expressed genes was performed using the STRING database (https://string-db.org/, accessed on 12 April 2026) to investigate relationships among the target genes.

### 2.8. qRT-PCR Analysis

For qRT-PCR analysis, liver tissues were collected among NCD, HFCD, and HFCD + HB-1 (*n* = 3). Total RNA was isolated from homogenized hepatic tissues utilizing a commercial total RNA kit, ensuring high purity for downstream applications. Total RNA, which was quantified and verified for quality, was reverse-transcribed into cDNA using the PrimeScript™ 1st Strand cDNA Synthesis Kit. The following reagents were added to a microcentrifuge tube on ice: 1 μg of total RNA (template), 1 μL of Oligo (dT) (50 uM), and 1 μL of dNTP Mix (10 mmol/L). The reaction volume was adjusted to 10 μm with RNase-free dH2O. After thorough mixing, the mixture was incubated at 65 °C for 5 min and immediately chilled on ice. The following components were added to the previously prepared mixture (10 μL) on ice: 4 μL of 5× Reaction Buffer, 0.5 μL of RNase Inhibitor (40 U/μL), and 1 μL of MMLV Reverse Transcriptase (200 U/μL). The total volume was adjusted to 20 μL with RNase-free dH2O. Finally, the reaction mixture was incubated at 42 °C for 30–60 min. The reaction was terminated by heating at 95 °C for 5 min. The resulting cDNA was then placed on ice for immediate use or stored for future analysis. Quantitative PCR (qPCR) was conducted in a 20 μL system containing 10 μL of 2× SYBR Green Master Mix, 0.4 μL of 10 μm specific forward and reverse primers, and 1 μL of cDNA. RNase-free dH2O was added to reach the final volume. This reaction (Reaction A) was applied to all target and housekeeping genes. The qPCR reaction was prepared according to the composition of Reaction A and performed using a real-time PCR system (EDC-810 thermal cycler, Eastwin Scientific Instrument Co., Ltd., Beijing, China). The thermal cycling conditions consisted of an initial denaturation at 95 °C for 5 min, followed by 40 cycles of denaturation at 95 °C for 15 s and annealing/extension at 60 °C for 30 s. Gene expression values were normalized against GAPDH to account for variations in RNA loading and reverse transcription efficiency. The 2^−ΔΔCt^ method was employed to assess relative expression levels of target genes, which were normalized and expressed as fold changes against the control group.

### 2.9. Western Blot Analysis

Liver tissues were homogenized in 2 mL tubes with steel beads and 200 μL of lysis buffer (containing 1% PMSF and 1% phosphatase inhibitors) using an automated tissue lyser. After a 30 min incubation on ice, the homogenates were centrifuged at 12,000 rpm for 5 min at 4 °C. The resulting supernatants were aliquoted and preserved at −20 °C. Protein concentration was determined using a BCA Protein Assay Kit. Bovine serum albumin (BSA) standards were prepared at concentrations of 1, 0.8, 0.6, 0.4, and 0.2 mg/mL, with PBS used as a blank control. Protein samples were diluted 10-fold by mixing 2 μL of the sample with 18 μL of PBS. Subsequently, 20 μL of each standard (in duplicate) and diluted sample (in triplicate) were added to a 96-well plate. BCA working solution (Reagent A: B = 50:1, *v*/*v*) was prepared, and 200 μL was added to each well. After incubation at 37 °C for 15 min in darkness, the optical density (OD) was measured at 562 nm using a DG-3022A microplate reader. The protein concentrations were calculated based on the linear regression equation derived from the BSA standard curve. The extracted protein supernatant was mixed with 5× protein loading buffer at a volume ratio of 4: 1 and boiled in a water bath for 10 min. After denaturation, the samples were cooled to room temperature and stored at −20 °C. The prepared gel was mounted in the electrophoresis tank, and running buffer was added to the reservoir. Protein samples and the molecular weight marker were loaded into the wells using a micropipette, with 40 μg total protein per sample. Electrophoresis was performed at a constant voltage of 180 V until the bromophenol blue dye front reached the bottom of the gel, which took approximately 60 min. After electrophoresis, the gel was transferred onto a PVDF membrane of the same size, which had been pre-activated in methanol for 30 s and equilibrated in transfer buffer. The gel and membrane were assembled in a transfer cassette with sponge pads according to the correct orientation, and the cassette was placed in the transfer apparatus. Proteins were transferred under the following conditions: <20 kDa, 1 cycle × 5 min; 20–250 kDa, 2 cycles × 5 min; >250 kDa, 3 cycles × 5 min. The PVDF membranes were blocked with TBST containing 5% non-fat milk for 2 h at room temperature on a shaker to minimize non-specific binding. For the detection of phosphorylated proteins, the membranes were blocked with 1% bovine serum albumin (BSA). Following blocking, the membranes were incubated with the appropriately diluted primary antibodies overnight at 4 °C. The specific antibody concentrations used in this study were as follows: anti-rabbit antibodies against Peroxisome proliferator-activated receptor γ (PPARγ) (1:1000, 58 kDa, Affinity, 16643-1-AP), FABP4 (1:1000, 15 kDa, Affinity, DF6035), CD36 (1:1000, 88 kDa, Affinity, DF13262), SREBP-1c (1:1000, 122 kDa, Affinity, AF6283), ADIPOQ (1:1000, 26 kDa, Affinity, DF7000), SCD-1 (1:1000, 41 kDa, Bioss, bs-3787R), p-P65 (1:1000, 65 kDa, Affinity, AF2006, Serine Ser536), TLR4 (1:1000, 100 kDa, Affinity, AF7017), IKKβ (1:1000, 87 kDa, Affinity, AF6010), and GAPDH (1:1000, 37 kDa, Xianzhi Biotech, AB-P-R 001). Excess primary antibody was removed by washing the PVDF membrane five times with TBST, 5 min per wash. The PVDF membrane was incubated with the appropriate HRP-conjugated secondary antibody diluted 1: 10,000 in TBST at room temperature on a shaker for 2 h. Excess secondary antibody was removed by washing the PVDF membrane five times with TBST, 5 min per wash. Protein bands were visualized using an ECL detection system. The PVDF membrane was placed protein-side up, and 200 μL of the working solution was applied evenly across the membrane surface. Signals were captured using an automated imaging system with optimized exposure settings. Blots were visualized using an ECL kit (KF8003, Affinity). The gray value of protein bands was analyzed by ipp.

### 2.10. Statistical Analysis

Partial data analysis and graphing were conducted via GraphPad Prism (version 10.1.2, San Diego, CA, USA), with results expressed as mean ± standard deviation (SD). Comparisons among 3 or more groups were analyzed by one-way ANOVA, with subsequent Dunnett’s post hoc testing. Student’s *t*-tests were employed to assess the significance between 2 treatment groups using a two-tailed calculation. Statistical significance was indicated as follows: **** for *p* < 0.0001, *** for *p* < 0.001, ** for *p* < 0.01, and * for *p* < 0.05.

## 3. Results

### 3.1. Untargeted Metabolomics Analysis

#### 3.1.1. Metabolites Identification Results

**Figure 1:**
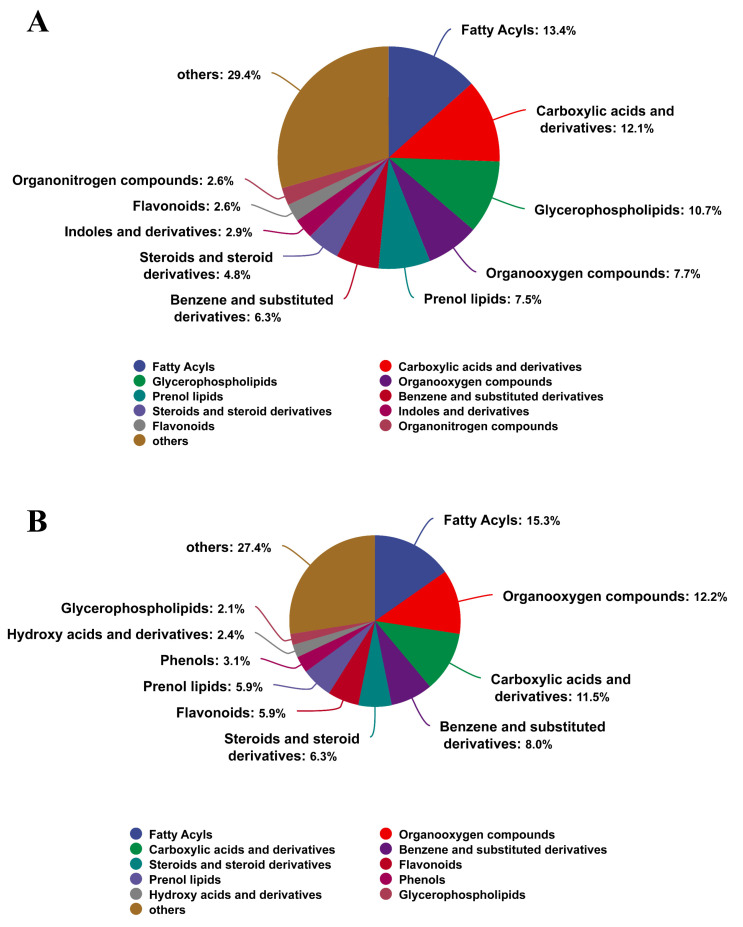
Metabolites identification analysis. (**A**) Metabolite classification in positive ion mode; (**B**) Metabolite classification in negative ion mode.

In Figure 1A, for LC-ESI (+)-MS analysis, fatty acyls (13.4%), carboxylic acids and derivatives (12.1%), glycerophospholipids (10.7%), organooxygen compounds (7.7%), prenol lipids (7.5%), benzene and substituted derivatives (6.3%), steroids and steroid derivatives (4.8%), indoles and derivatives (2.9%), flavonoids (2.6%), and organonitrogen compounds (2.6%) were identified as the major metabolite classes.

In Figure 1B, for LC-ESI (−)-MS analysis, fatty acyls (15.3%), organooxygen compounds (12.2%), carboxylic acids and derivatives (11.5%), benzene and substituted derivatives (8.0%), steroids and steroid derivatives (6.3%), flavonoids (5.9%), prenol lipids (5.9%), phenols (3.1%), hydroxy acids and derivatives (2.4%), and glycerophospholipids (2.1%) were identified as the major metabolite classes.

#### 3.1.2. Expression Abundance Analysis

The expression abundance of metabolites was statistically analyzed and organized through density distribution to comprehensively examine the expression abundance patterns of all substances across various samples. The statistical results were visualized using violin plots. In Figure S1A,B, whether in LC-ESI (+)-MS or LC-ESI (−)-MS, the violin plot results for samples were similar, indicating that their overall metabolite profiles were closely aligned, reflecting high similarity.

The correlation of metabolites’ expression levels was considered an important indicator for assessing experimental reliability and the appropriateness of sample selection. Before performing differential expression analysis, the correlation of metabolites expression levels was examined. Pearson correlation coefficient was used to represent the correlation of metabolites’ expression levels. In Figure S1C,D, whether in LC-ESI (+)-MS or LC-ESI (−)-MS, the results indicated that the differences between HB, HB-1, HB-2, and HB-3 were small, with high similarity, while the differences were large, with low similarity. In Figure S1E,F, whether in LC-ESI (+)-MS or LC-ESI (−)-MS, the results indicated that the differences among the four HB samples were large.

#### 3.1.3. Multivariate Statistical Analysis and Differential Metabolite Screening

As can be seen from Figure S2, Principal Component Analysis (PCA), Partial Least Squares-Discriminant Analysis (PLS-DA) and Orthogonal Partial Least Squares Discriminant Analysis (OPLS-DA) results showed that HB and HB-1 exhibited considerable separation, reflecting a significant distinction between HB and HB-1. In Figure S3 and Table S3, based on FC ≥ 2 or ≤ 0.5, *p*-value < 0.05, vip > 1 and FDR < 0.05, compared with HB, HB-1 significantly increased the levels of Kaempferol, Isoferulic acid, Palmitoylethanolamide, Gallic acid, Luteolinidin, and so on. As can be seen from Figure S4, PCA, PLS-DA and OPLS-DA results showed that HB and HB-2 exhibited considerable separation, reflecting a significant distinction between HB and HB-2. In Figure S5 and Table S4, compared with HB, HB-2 significantly increased the levels of Genistein, Quercetin, Caffeic acid, Kaempferol, Sinapyl alcohol, and so on. As can be seen from Figure S6, PCA, PLS-DA and OPLS-DA results showed that HB and HB-3 exhibited considerable separation, reflecting a significant distinction between HB and HB-3. In Figure S7 and Table S5, compared with HB, HB-3 significantly increased the levels of Kaempferol, Genistein, Luteolinidin, Quercetin, Naringin, Hesperetin, Baicalein, Gallic acid, Betaine, Curcolone, and so on. In addition, in Figure S8A, the Venn plot indicated that the identified average numbers of differential metabolites among HB vs. HB-1, HB vs. HB-2, and HB vs. HB-3 were 259, 397, and 346, respectively. The distinct metabolites among HB vs. HB-1, HB vs. HB-2, and HB vs. HB-3 were 48, 186, and 135, respectively. In Figure S9B, the number of up- and down-regulated metabolites among HB vs. HB-1 were 125 and 293. The number of up- and down-regulated metabolites among HB vs. HB-2 were 346 and 320. The number of up- and down-regulated metabolites among HB vs. HB-3 were 453 and 185.

#### 3.1.4. Differential Metabolites Trend

Trend analysis was conducted based on the bidirectional clustering heatmap results obtained from the aforementioned clustering analysis. Metabolites were further divided into distinct clusters (defaulting to four) according to the similarity in their expression patterns. Metabolites within each cluster were categorized as belonging to the same group, likely performing similar functions. The blue trend lines in the figure visually illustrated the changes in expression abundance of different types of metabolites across samples, enabling a narrowing of the analysis scope to focus on key metabolites. The differential metabolites trend results among HB vs. HB-1, HB vs. HB-2, and HB vs. HB-3 were shown in Figures S9B, S10B and S11B.

The results of the random forest analysis highlighted the importance of differential metabolites among different treatment groups. Metabolites with high importance scores were identified as key markers distinguishing the groups. As shown in Figure S9A, the important key differential metabolites of HB-1 were Chimyl alcohol, xi-3-Methyldodecane, (Z)-[(4-hydroxyphenyl)acetaldehyde oxime], 5-Acetyl-2,3-dihydro-1,4-thiazine, Annuolide C, and so on. As shown in Figure S10A, the important key differential metabolites of HB-2 were N1-trans-Feruloylagmatine, 4-Gallocatechol, *N*-(gamma-Glutamyl)ethanolamine, *N*-Methyl-4-dimethylallyltryptophan, Mannose 6-phosphate, and so on. As shown in Figure S11A, the important key differential metabolites of HB-3 were Menthone, 10-Hydroxydecanoic acid, Geranyl Phosphate, FA 20_3; O4, xi-3-Methyldodecane, and so on.

#### 3.1.5. Differential Metabolites Enrichment Analysis

Based on the differential metabolites among HB, HB-1, HB-2, and HB-3, differential metabolic pathways were identified based on the Kyoto Encyclopedia of Genes and Genomes (KEGG) database, and the top-enriched pathways were visualized using bubble charts to illustrate the impact and significance of the treatments. As shown in Figure S12 and Table S6, the significant KEGG pathways of HB vs. HB-1 were aminoacyl-tRNA biosynthesis, D-amino acid metabolism, biosynthesis of amino acids, Cutin, suberine and wax biosynthesis, ABC transporters, and so on. As shown in Figure S13 and Table S7, the significant KEGG pathways of HB vs. HB-2 were arginine biosynthesis, biosynthesis of amino acids, phenylpropanoid biosynthesis, ABC transporters, Flavone and flavonol biosynthesis and so on. As shown in Figure S14 and Table S8, the significant KEGG pathways of HB vs. HB-3 were arginine biosynthesis, Flavone and flavonol biosynthesis, tyrosine metabolism, alpha-Linolenic acid metabolism, Cutin, suberine and wax biosynthesis, and so on.

#### 3.1.6. Multiple Group Comparison Analysis

**Figure 2:**
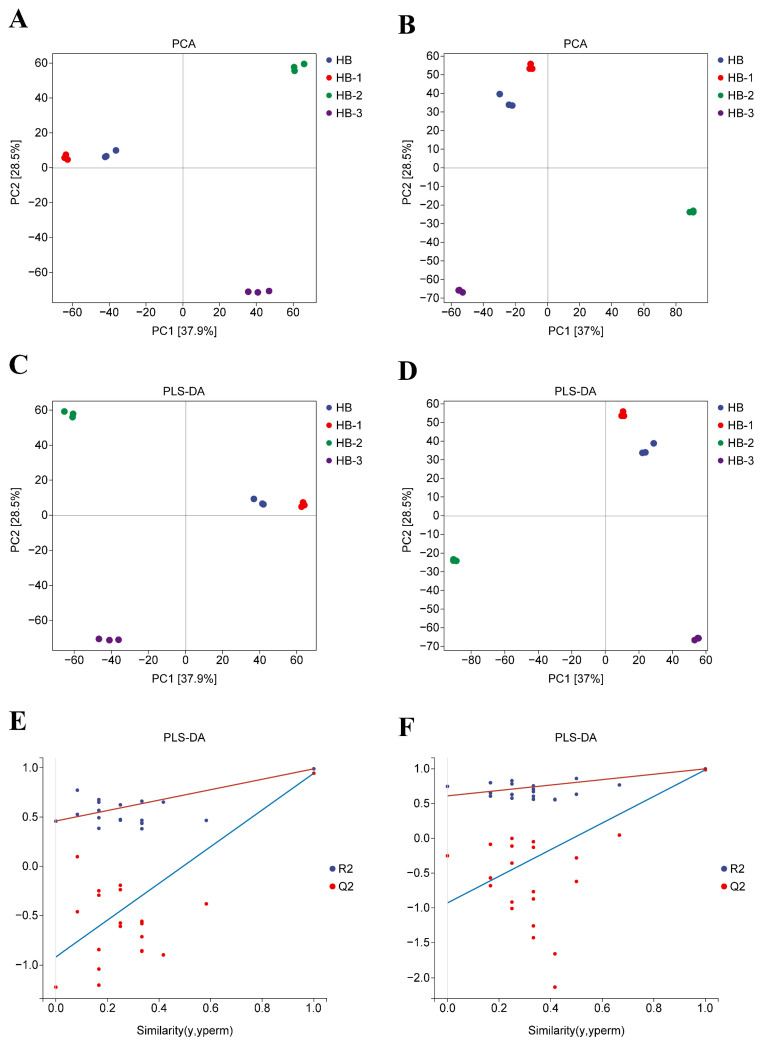
Multivariate statistical analysis among HB, HB-1, HB-2, and HB-3. (**A**) PCA plot in LC-ESI (+)-MS, R^2^X (cum) 0.664, pre 2; (**B**) PCA plot in LC-ESI (−)-MS, R^2^X (cum) 0.655, pre 2; (**C**,**E**) PLS-DA plot in LC-ESI (+)-MS, R^2^X (cum) 0.767, R^2^Y (cum) 0.99, Q^2^ (cum) 0.945, pre 3; (**D**,**F**) PLS-DA plot in LC-ESI (−)-MS R^2^X (cum) 0.822, R^2^Y (cum) 0.999, Q^2^ (cum) 0.985, pre 4.

**Figure 3:**
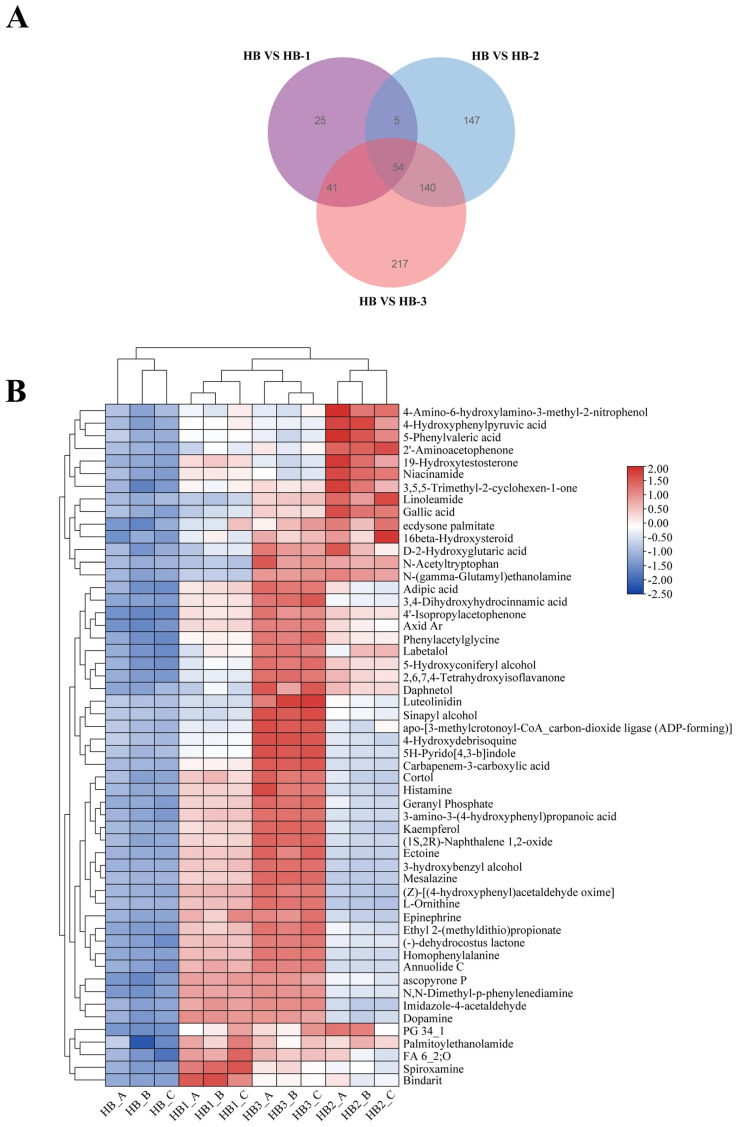
Differential metabolites result among HB, HB-1, HB-2, and HB-3. (**A**) Venn plot; (**B**) the main differential metabolites of HB-1, HB-2, and HB-3 compared with HB.

The differences among three or more groups were statistically analyzed using the Kruskal–Wallis test and ANOVA method. The obtained key differential metabolites among HB, HB-1, HB-2, and HB-3 are shown in Figure S15. As can be seen from Figure 2, PCA and PLS-DA results showed that HB, HB-1, HB-2, and HB-3 exhibited considerable separation, reflecting a significant distinction. As illustrated in Figure 3 and Table S9, based on FC ≥ 2 or ≤0.5, *p*-value < 0.05, vip > 1 and FDR < 0.05, compared with HB, the important key differential metabolites were Kaempferol, Quercetin, Genistein, Gallic acid, Isoferulic acid, and so on. The important key differential metabolites are shown in Figure S16A. The differential metabolites trend results are shown in Figure S16B. As shown in Figure S17 and Table S10, the significant KEGG pathways among HB, HB-1, HB-2, and HB-3 were linoleic acid metabolism, biosynthesis of amino acids, Cutin, suberine and wax biosynthesis, alpha-linolenic acid metabolism, arginine biosynthesis, and so on.

### 3.2. Transcriptomics Results

As shown in Figure S18, among the differentially regulated genes between HFCD and HFCD + HB-1, 97 genes were increased and 77 genes were decreased. Potential biological DEGs were screened and selected using thresholds of |log2FoldChange| > 1 and *p* < 0.05 to compare the transcriptomic profiles between HFCD and HFCD + HB-1.

**Figure 4:**
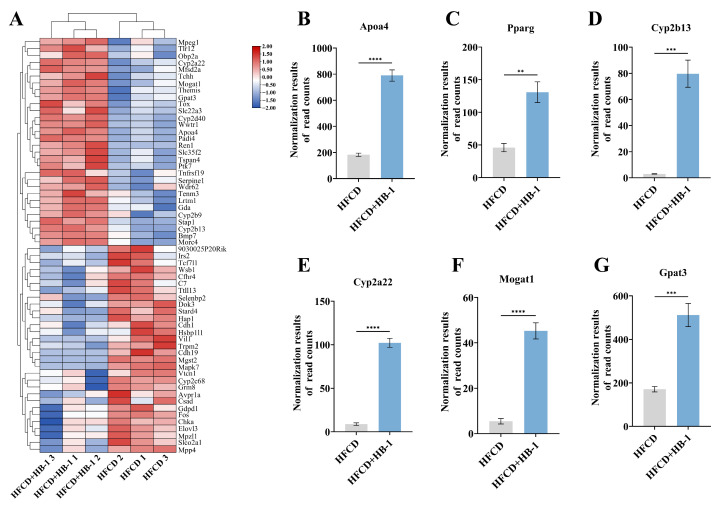
The main differential genes between HFCD and HFCD + HB-1. Data was visualized as mean ± SD (*n* = 3). (**A**) Main differential genes between HFCD and HFCD+HB-1; (**B**) Relative expression level of *Apoa4*; (**C**) Relative expression level of *paarg*; (**D**) Relative expression level of *Cyp2b13*; (**E**) Relative expression level of *Cyp2a22*; (**F**) Relative expression level of *Mogat1*; (**G**) Relative expression level of *Gpat3*. *T*-tests were used to analyze the significance between HFCD and HFCD + HB-1 based on two-tailed calculation; *p* < 0.0001 was shown as ****, *p* < 0.001 was shown as ***, and *p* < 0.01 was shown as **.

As illustrated in Figure 4 and Table S11, the expression levels of DEGs were significantly regulated in HFCD + HB-1 compared with HFCD including *Apoa4*, *Pparg*, *Cyp2b13*, *Cyp2a22*, *Mogat1*, *Gpat3*, *Cidea*, *Timp1*, *Plin4*, *Gck*, *Lgals1*, *Vnn1*, and *Oxct1*. As illustrated in Figure S19 and Table S12, the main GO enrichment pathways of HFCD and HFCD + HB-1 were response to oxygen-containing compound, regulation of fatty acid metabolic process, monocarboxylic acid metabolic process, cellular ketone metabolic process, cellular response to oxygen-containing compound, cellular response to chemical stimulus, fatty acid metabolic process, regulation of cellular ketone metabolic process, carboxylic acid metabolic process, response to xenobiotic stimulus, response to stilbenoid, oxoacid metabolic process, and organic acid metabolic process.

**Figure 5:**
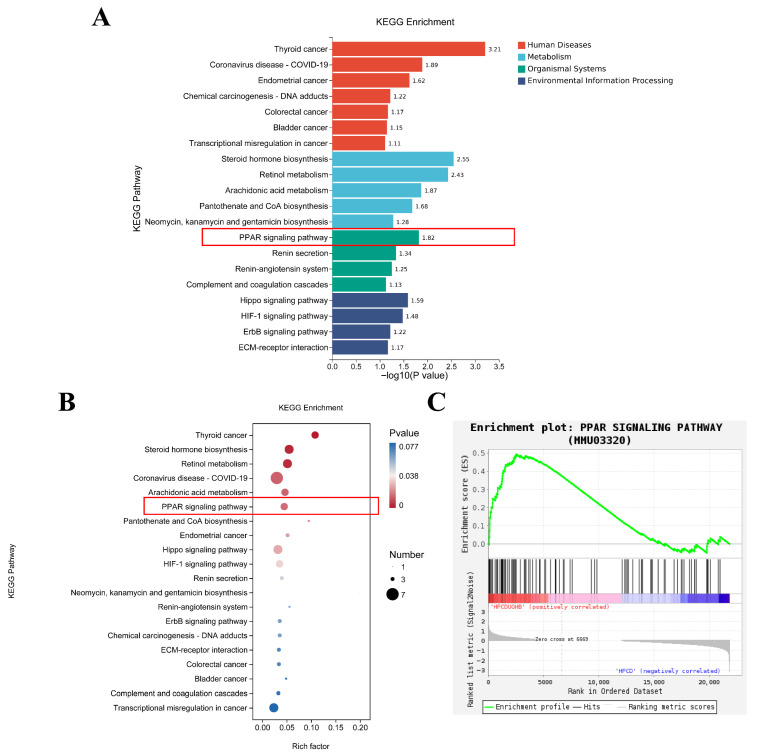
KEGG enrichment pathways analysis between HFCD and HFCD+HB-1. (**A**) Bar chart of KEGG pathway enrichment; (**B**) Bubble plot of KEGG pathway enrichment; (**C**) Gene Set Enrichment Analysis (GESA) plot.

As illustrated in Figure 5 and Table S13, the main KEGG pathways of HFCD and HFCD + HB-1 were steroid hormone biosynthesis, retinol metabolism, PPAR signaling pathway, arachidonic acid metabolism, cholesterol metabolism, linoleic acid metabolism, fat digestion and absorption, and pantothenate and CoA biosynthesis.

### 3.3. qRT-PCR Results

**Figure 6:**
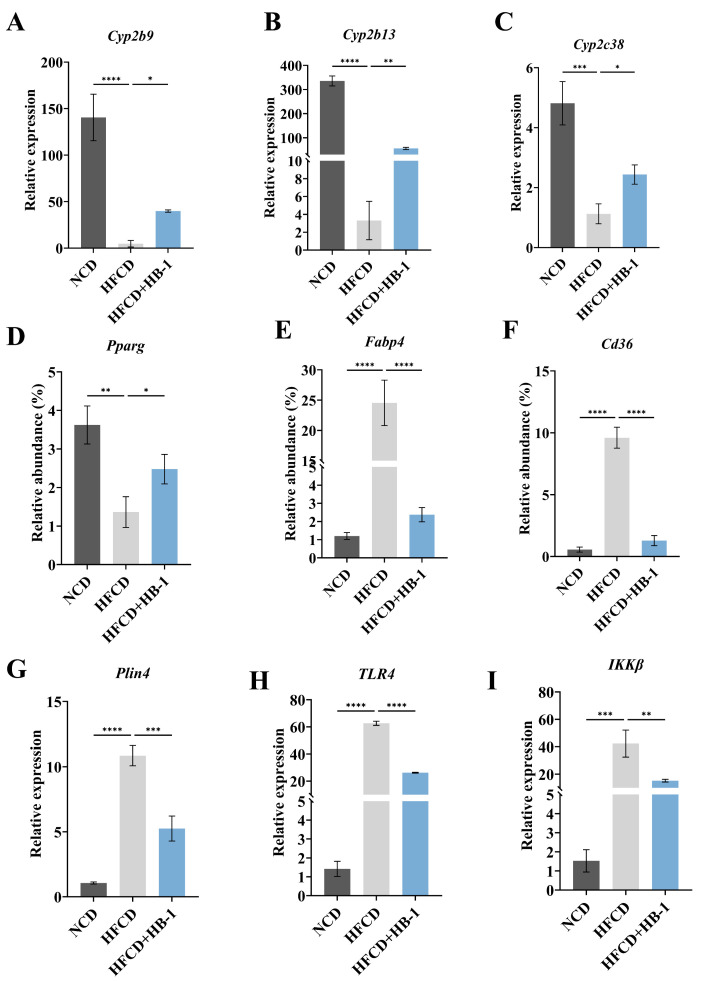
Gene expression levels of (**A**) *Cyp2b9*, (**B**) *Cyp2b13*, (**C**) *Cyp2c38*, (**D**) *Pparg*, (**E**) *Fabp4*, (**F**) *Cd36*, (**G**) *Plin4*, (**H**) *TLR4*, and (**I**) *IKKβ* in liver tissues among NCD, HFCD, and HFCD + HB-1. Data were expressed as mean ± SD (*n* = 3) based on Dunnett’s test. * indicated *p* < 0.05, ** indicated *p* < 0.01, *** indicated *p* < 0.001, **** indicated *p* < 0.0001.

Quantitative validation (Figure 6A–C) confirmed a significant increase in the expression of Cyp-related genes (*Cyp2b9*, *Cyp2b13*, and *Cyp2c38*) following HB-1 administration in HFCD-fed mice (*p* < 0.05). The results were according to the results of RNA-seq transcriptomics analysis and further validated it.

As illustrated in Figure 6D–G, the transcriptomic findings were corroborated by qRT-PCR analysis. Compared with the HFCD, the mRNA expression level of PPARG was significantly up-regulated, whereas the expression levels of Fabp4, Cd36, and Plin4 were markedly down-regulated in the HFCD + HB-1 group (*p* < 0.05). Lipid metabolism dysregulation in HFCD-induced hyperlipidemia mice might be alleviated by the HB-1 treatment, which was mediated by participating in the PPAR signaling pathway.

As illustrated in Figure 6H,I, specifically, the mRNA expression levels of TLR4 and IKKβ were obviously down-regulated in the HFCD + HB-1 compared to HFCD (*p* < 0.05). The results suggested that HB-1 might regulate the attenuation of inflammatory signaling markers in HFCD-induced hyperlipidemia mice by participating in the NF-κB pathway.

### 3.4. Western Blot Results

**Figure 7:**
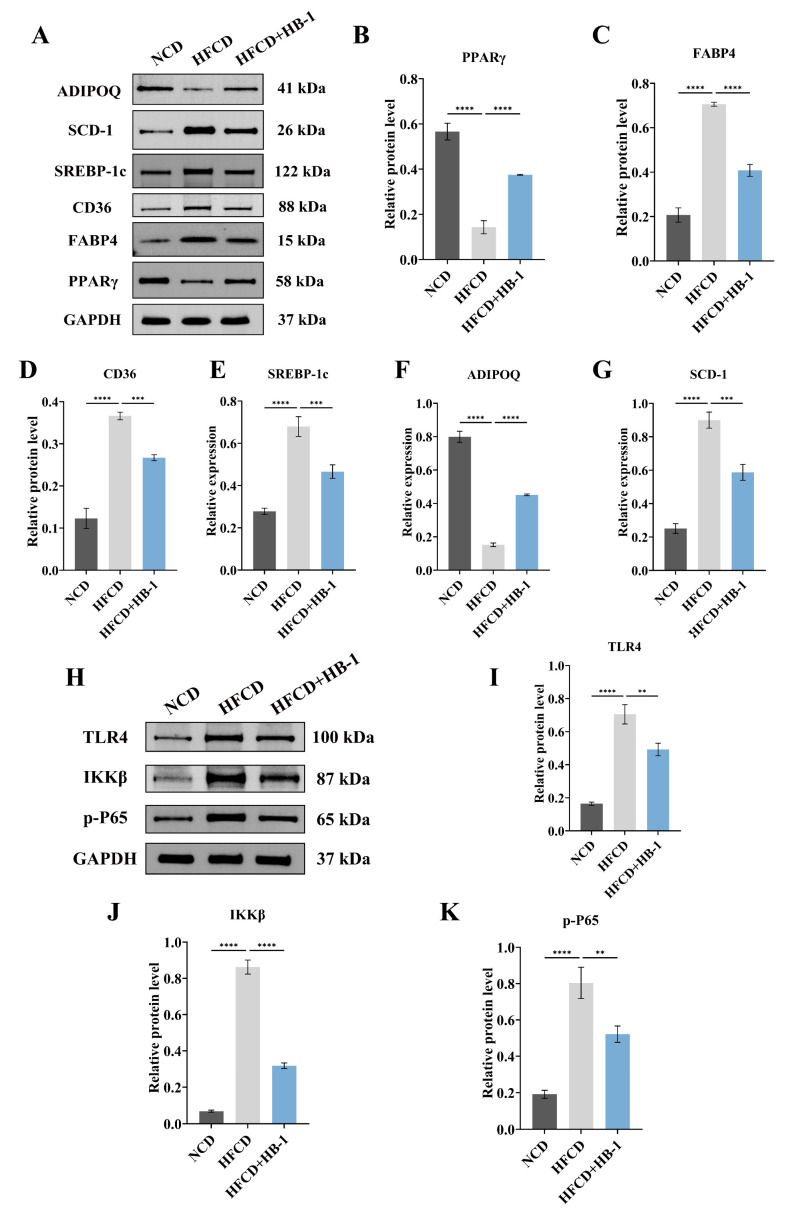
Protein expression levels (**A**,**H**) including (**B**) PPARγ, (**C**) Fabp4, (**D**) CD36, (**E**) SREBP-1c, (**F**) ADIPOQ, (**G**) SCD-1, (**I**) TLR4, (**J**) IKKβ, and (**K**) p-P65 in liver tissues among NCD, HFCD, and HFCD + HB-1. Data were expressed as mean ± SD (*n* = 3) based on Dunnett’s test. ** indicated *p* < 0.01, *** indicated *p* < 0.001, **** indicated *p* < 0.0001.

As illustrated in Figure 7A–G, compared with HFCD, the levels of PPARγ and ADIPOQ were obviously elevated in HFCD + HB-1, whereas the levels of FABP4, CD36, SREBP-1c, and SCD-1 were markedly reduced (*p* < 0.05). These findings demonstrated that HB-1 might ameliorate HFCD-induced lipid metabolism dysregulation in hyperlipidemia mice by participating in the PPARγ signaling pathway.

As illustrated in Figure 7H–K, compared with HFCD, TLR4, IKKβ, and p-P65 levels were significantly reduced in HFCD + HB-1 (*p* < 0.05). These findings suggested that HB-1 might regulate the attenuation of inflammatory signaling markers of liver in hyperlipidemia mice byparticipating in the TLR4/NF-κB signaling pathway.

## 4. Discussion

The cell wall and outer layer of HB were mainly composed of β-glucan, hemicellulose, araboxylan, cellulose, and pectin, which belong to non-starch polysaccharides. It is hard to grind, process, and fully release the bioactive ingredients of HB due to the protection of HB by these bioactive components and organizational structures from damage. Therefore, the innovation and application of food processing technologies to modify the cell wall and outer layer of HB have been widely accepted in barley-based foods in the field of the whole-grain foods industry, which is rich in abundant bioactive ingredients. In this study, the three different modification processes, including microwave fluidization, extrusion and puffing, and ultrafine pulverization, were used to modify HB, and untargeted metabolomics based on LC-MS were used to analyze the detailed differences in metabolites or related pathways before or after modification. A total of 1292 metabolites were identified. Based on their foundational chemical attributes and structures, these metabolites were categorized into 78 distinct classes. Among these, fatty acyls, carboxylic acids and derivatives, glycerophospholipids, organooxygen compounds, prenol lipids, benzene and substituted derivatives, steroids and steroid derivatives, indoles and derivatives, flavonoids, organonitrogen compounds, phenols, and glycerolipids were the main components of HB, HB-1, HB-2, and HB-3. The results were in agreement with the previous study, indicating the abundant secondary metabolites distributed within Qingke’s grains, which are mainly found in high-altitude regions [25]. Compared with HB, HB-1, HB-2, and HB-3 all increased the levels of amino acids and their derivatives (e.g., l-Ornithine, Homophenylalanine, 3-amino-3-(4-hydroxyphenyl) propanoic acid, *N*-Acetyltryptophan), phenols (e.g., Kaempferol, Gallic acid, Luteolinidin, 4-Hydroxyphenylpyruvic acid, Sinapyl alcohol, 5-Hydroxyconiferyl alcohol), carboxylic acid and its derivatives (e.g., Carbapenem-3-carboxylic acid, Adipic acid, 3,4-Dihydroxyhydrocinnamic acid, 5-Phenylvaleric acid, d-2-Hydroxyglutaric acid), flavonoids (e.g., Kaempferol, 2,6,7,4-Tetrahydroxyisoflavanone), and so on. Moreover, all three different modification processes increased the levels of amino acids and their derivatives. The denaturation and breakdown of Qingke’s proteins under high temperature, pressure, and shear force were the primary reasons for this phenomenon, leading to a higher concentration of free amino acids [26]. In addition, compared with HB, HB-1 increased the levels of Kaempferol, Isoferulic acid, Palmitoylethanolamide, Gallic acid, Luteolinidin, and so on. Compared with HB, HB-2 increased the levels of Genistein, Quercetin, Caffeic acid, Kaempferol, Sinapyl alcohol, and so on. Compared with HB, HB-3 increased the levels of Kaempferol, Genistein, Luteolinidin, Quercetin, Naringin, Hesperetin, Baicalein, Gallic acid, Betaine, Curcolone, and so on. Moreover, all three different modification processes regulated the pathways of linoleic acid metabolism, biosynthesis of amino acids, Cutin, suberine, and wax biosynthesis alpha-linolenic acid metabolism, arginine biosynthesis, and so on.

**Figure 8:**
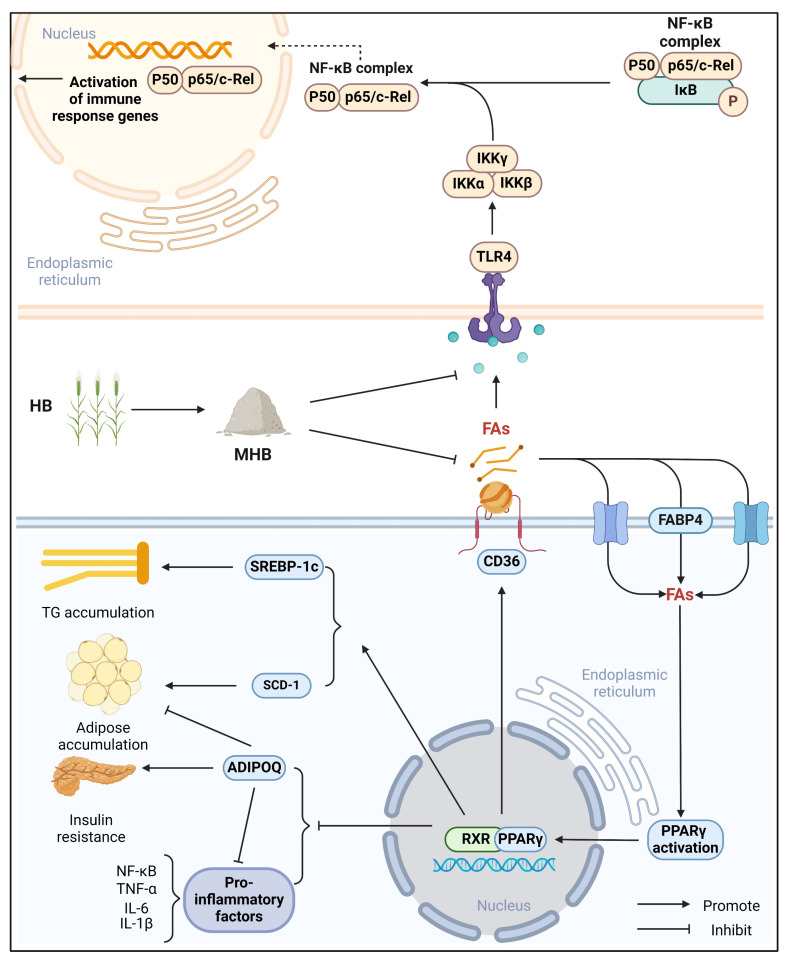
The detailed mechanism of regulating lipid metabolism and attenuation of inflammatory signaling markers by MHB.

As illustrated in Figure 8, PPAR signaling pathway was proved to be primarily governed by fatty acid β-oxidation. Fabp4, CD36, SREBP-1c, ADIPOQ, and SCD-1 were observed to facilitate a cascade of metabolic events, ranging from triacylglycerol storage and fatty acid transport to the development of lipid droplets and hepatic adipose deposition [27,28]. SREBP-1c could promote TG synthesis, which is a transmembrane protein distributed in the endoplasmic reticulum. The previous studies had proved that lipogenic genes or proteins including FAS, ACC, and SCD-1 controlled by SREBP-1c were obviously up-regulated in HFD-fed mice liver [29]. In this study, compared with HFCD, HFCD + HB-1 significantly increased the expression levels of ADIPOQ, while reducing the expression levels of Fabp4, CD36, SREBP-1c, and SCD-1. The results obtained were according to the previous study [30]. SCD-1 is widely recognized as a pivotal enzyme during the pathway of de novo fatty acid synthesis. Moreover, SCD-1 enhances the synthesis of triacylglycerols, and transforms saturated fatty acids (SFA) into monounsaturated fatty acids (MUFA). HFCD caused the increase in the level of SCD-1 in hyperlipidemia liver, indicating the regulation mediated by key enzymes involved in controlling lipid biosynthesis. However, HFCD + HB-1 significantly inhibit this trend. CD36 plays a key role in different aspects of FAS metabolism, including lipid intake, transport, and utilization [31]. CD36 is accepted as a receptor characterized by a high affinity for long-chain fatty acids. It is closely associated with fat metabolism due to its high expression in adipose tissue. Moreover, the increase in CD36 level could result in hepatic steatosis in HFD mice due to the excessive intake of FAS [32]. FABP4 is one of the key carrier proteins in adipose accumulation. FABP4 is located on the cell membrane and is a carrier protein responsible for transferring FAS from the outside of the membrane to the inside of the membrane, thereby promoting fat accumulation and fat droplet formation, inhibiting ADIPOQ, and further leading to liver fat accumulation and lipid metabolism disorders, leading to hyperlipidemia, NAFLD, and NASH [33]. In this study, HB-1 might regulate lipid metabolism disorders via participating in PPARγ pathway.

Moreover, to validate the results of inflammatory signaling markers induced by HFCD, the TLR4/NF-κB pathway key proteins were analyzed. In this study, HB-1 significantly decreased the expression levels of TLR4, IKKβ, and p-P65. The innate immune system is activated after the body is exposed to external stimuli, causing the activation of TLR4 expression. This leads to specific immune responses and the activation of NF-κB expression [34]. NF-κB is closely related to stress response, inflammatory response, apoptosis regulation, and immune response, which functions as a critical nuclear transcription factor. p65 and p50 subunits, whether homodimer or heterodimer, are the typical forms of NF-κB protein. NF-κB is regulated through its interaction with the IκB inhibitory protein in the cytoplasm, forming a trimeric complex [35]. When liver cells are stimulated by external factors including fatty acids, cholesterol, and fat, TLR4 is activated and exposed to external stimuli, causing the activation of NF-κB, inducing the production of proinflammatory cytokines such as TNF-α, IL-1β, and IL-6, thereby inducing inflammation [36]. In this study, HB-1 might regulate the attenuation of inflammatory signaling markers via participating in the TLR4/NF-κB pathway.

In summary, based on LC-MS untargeted metabolomics, microwave fluidization, extrusion and puffing, ultrafine pulverization could increase the bioactive ingredients of HB including Kaempferol, Genistein, Quercetin Gallic acid, Luteolinidin, and so on. In addition, based on RNA-seq transcriptomics data, qRT-PCR, and WB analysis, the results indicated that HB-1 might improve lipid metabolism disorders via participating in the PPARγ pathway, which increases the protein expression levels of ADIPOQ, while decreasing the expression levels of Fabp4, CD36, SREBP-1c, and SCD-1. HB-1 might regulate the attenuation of inflammatory signaling markers via participating in the TLR4/NF-κB pathway, which decreases the expression levels of TLR4, IKKβ, and p-P65. Meanwhile, there were several limitations in this study. Only partial genes and proteins in the PPARγ and TLR4/NF-κB pathways were validated. Therefore, the inhibitory effects on these signaling pathways could not be fully confirmed. In addition, no functional experiments, such as pathway inhibition or activation assays, were conducted, and the causal relationship between HB-1 and these signaling pathways remain to be further elucidated.

## Data Availability

The original contributions presented in the study are included in the article/Supplementary Material, further inquiries can be directed to the corresponding author.

## Supplementary Materials

The following supporting information can be downloaded at: https://www.mdpi.com/article/10.3390/foods15081396/s1, Figure S1. Expression abundance analysis. (A) Abundance violin plot in LC-ESI (+)-MS; (B) Abundance violin plot in LC-ESI (-)-MS; (C) Samples correlation analysis in LC-ESI (+)-MS; (D) Samples correlation analysis in LC-ESI (-)-MS; (E) Overall metabolites clustering diagram in LC-ESI (+)-MS; (F) Overall metabolites clustering diagram in LC-ESI (-)-MS. Figure S2. Multivariate statistical analysis of HB VS HB-1. (A) PCA plot in LC-ESI (+)-MS; (B) PCA plot in LC-ESI (-)-MS; (C and E) PLS-DA plot in LC-ESI (+)-MS; (D and F) PLS-DA plot in LC-ESI (-)-MS; (G) OPLS-DA plot in LC-ESI (+)-MS; (H) OPLS-DA plot in LC-ESI (-)-MS. Figure S3. Differential metabolites result between HB and HB-1. (A) Volcano plot in positive mode; (B) Volcano plot in negative mode; (C) Significantly differential metabolites between HB and HB-1; (D) Main metabolites related to lipid metabolism; (E) Main metabolites related to anti-inflammation. Figure S4. Multivariate statistical analysis of HB VS HB-2. (A) PCA plot in LC-ESI (+)-MS; (B) PCA plot in LC-ESI (-)-MS; (C and E) PLS-DA plot in LC-ESI (+)-MS; (D and F) PLS-DA plot in LC-ESI (-)-MS; (G) OPLS-DA plot in LC-ESI (+)-MS; (H) OPLS-DA plot in LC-ESI (-)-MS. Figure S5. Differential metabolites result between HB and HB-2. (A) Volcano plot in positive mode; (B) Volcano plot in negative mode; (C) Significantly differential metabolites between HB and HB-1; (D) Main metabolites related to anti-inflammation; (E) Main metabolites related to lipid metabolism. Figure S6. Multivariate statistical analysis of HB VS HB-3. (A) PCA plot in LC-ESI (+)-MS; (B) PCA plot in LC-ESI (-)-MS; (C and E) PLS-DA plot in LC-ESI (+)-MS; (D and F) PLS-DA plot in LC-ESI (-)-MS; (G) OPLS-DA plot in LC-ESI (+)-MS; (H) OPLS-DA plot in LC-ESI (-)-MS. Figure S7. Differential metabolites result between HB and HB-3. (A) Volcano plot in positive mode; (B) Volcano plot in negative mode; (C) Significantly differential metabolites between HB and HB-1; (D) Main metabolites related to lipid metabolism; (E) Main metabolites related to anti-inflammation. Figure S8. (A) Venn analysis; (B) Number of up-regulated and down-regulated metabolites among HB VS HB-1, HB VS HB-2, and HB VS HB-3. Figure S9. (A) Random Forest plot of HB VS HB-1; (B) Differential metabolites trend analysis. Figure S10. (A) Random Forest plot of HB VS HB-2; (B) Differential metabolites trend analysis. Figure S11. (A) Random Forest plot of HB VS HB-3; (B) Differential metabolites trend analysis. Figure S12. (A) KEGG enrichment bar plot of HB VS HB-1; (B) KEGG enrichment factor plot of HB VS HB-1. Figure S13. (A) KEGG enrichment bar plot of HB VS HB-2; (B) KEGG enrichment factor plot of HB VS HB-2. Figure S14. (A) KEGG enrichment bar plot of HB VS HB-3; (B) KEGG enrichment factor plot of HB VS HB-3. Figure S15. Multiple group comparison analysis at positive mode (A) and negative mode (B) based on Kruskal-Wallis test and ANOVA analysis. Figure S16. (A) Random Forest plot among HB, HB-1, HB-2, and HB-3; (B) Differential metabolites trend analysis. Figure S17. (A) KEGG enrichment bar plot among HB, HB-1, HB-2, and HB-3; (B) KEGG enrichment factor plot among HB, HB-1, HB-2, and HB-3. Figure S18. Liver transcriptomic analysis between HFCD and HFCD+HB-1. (A) Volcano plot; (B) HFCD and HFCD+HB-1 PCA plot; (C) Up-regulated and down-regulated differential genes. Figure S19. GO enrichment pathways analysis between HFCD and HFCD+HB-1. Table S1. Mouse basic diet composition and nutrition level. Table S2. The selected genes and sequences of primers. Table S3. The top 30 differential metabolites analysis between HB and HB-1. Table S4. The top 30 differential metabolites analysis between HB and HB-2. Table S5. The top 30 differential metabolites analysis between HB and HB-3. Table S6. The top 50 significant KEGG pathways between HB and HB-1. Table S7. The top 50 significant KEGG pathways between HB and HB-2. Table S8. The top 50 significant KEGG pathways between HB and HB-3. Table S9. The top 50 differential metabolites analysis among HB, HB-1, HB-2 and HB-3. Table S10. The top 50 significant KEGG pathways among HB, HB-1, HB-2 and HB-3. Table S11. The top 50 differential genes analysis between HFCD and HFCD+HB-1. Table S12. The top 50 significant GO pathways between HFCD and HFCD+HB-1. Table S13. The top 50 significant KEGG pathways between HFCD and HFCD+HB-1. Uncropped, unprocessed and full gel and blot.
