# Supplementary material for: Chemical Composition Analysis of Highland Barley (Hordeum vulgare L.) with Different Modification Methods and Lipid Metabolism Mechanism Analysis of Highland Barley with Microwave Fluidization Modification
Source: Foods. 2026 Apr 17;15(8):1396. doi: 10.3390/foods15081396 (PMC13114515; doi:10.3390/foods15081396)
Supplement: Supplementary file 1 [file foods-15-01396-s001.zip › Figure S1.pdf]

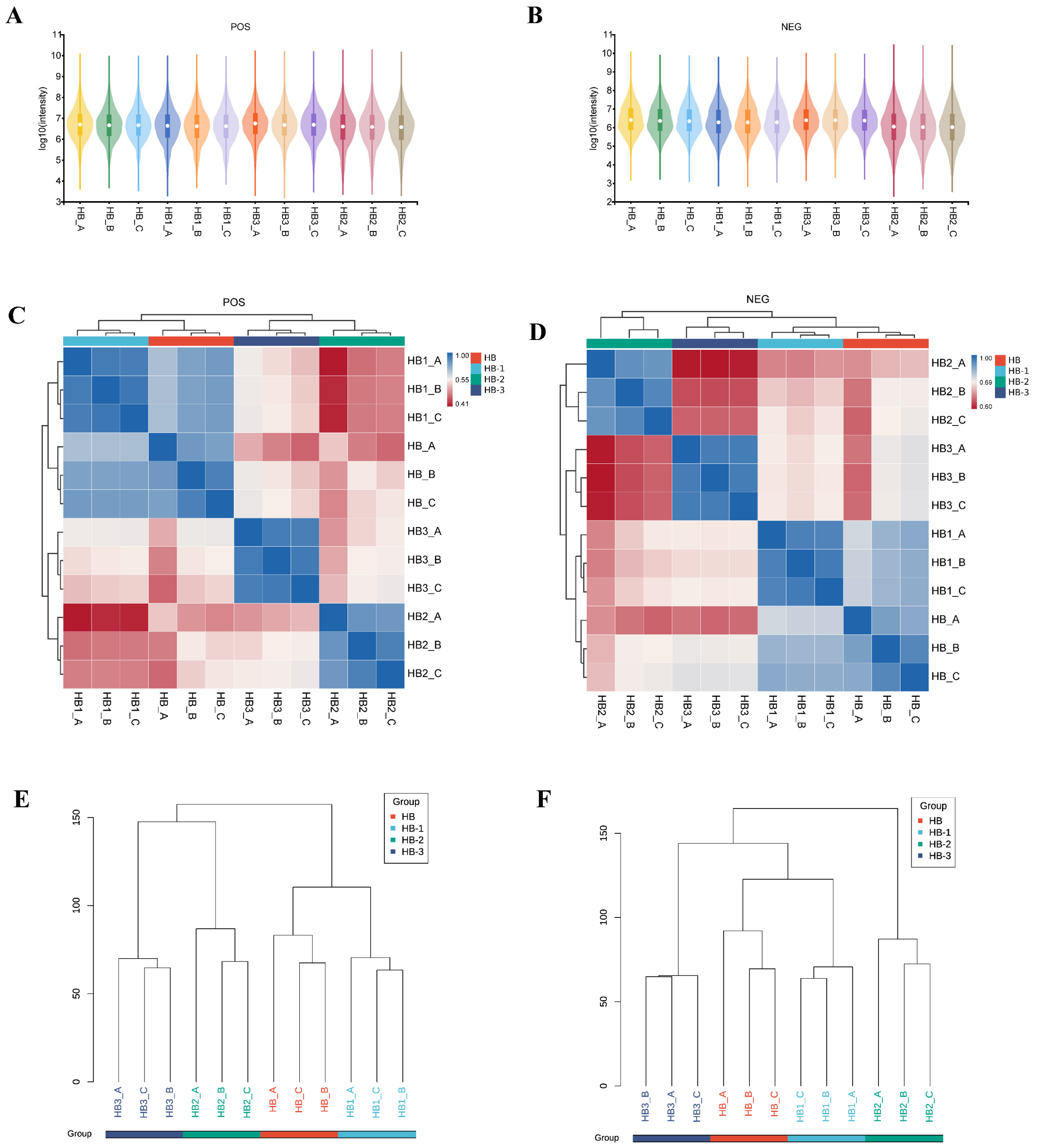

Figure S1 Expression abundance analysis. (A) Abundance violin plot in LC-ESI (+)-MS; (B) Abundance violin plot in LC-ESI (-)-MS; (C) Samples correlation analysis in LC-ESI (+)-MS; (D) Samples correlation analysis in LC-ESI (-)-MS; (E) Overall metabolites clustering diagram in LC-ESI (+)-MS; (F) Overall metabolites clustering diagram in LC-ESI (-)-MS.
