# Supplementary material for: Chemical Composition Analysis of Highland Barley (Hordeum vulgare L.) with Different Modification Methods and Lipid Metabolism Mechanism Analysis of Highland Barley with Microwave Fluidization Modification
Source: Foods. 2026 Apr 17;15(8):1396. doi: 10.3390/foods15081396 (PMC13114515; doi:10.3390/foods15081396)
Supplement: Supplementary file 1 [file foods-15-01396-s001.zip › Figure S17.pdf]

A

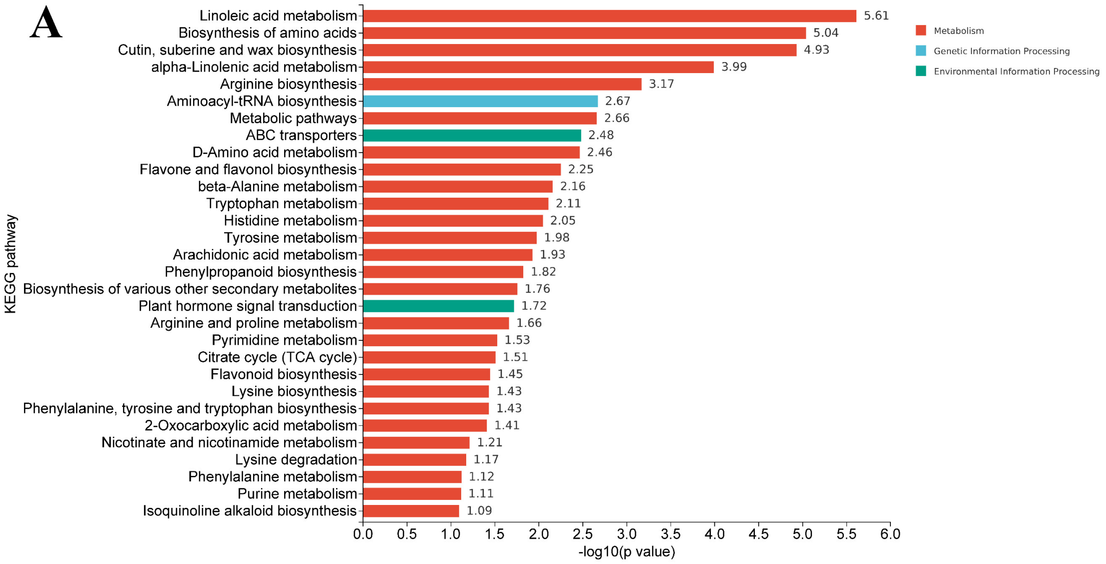

B

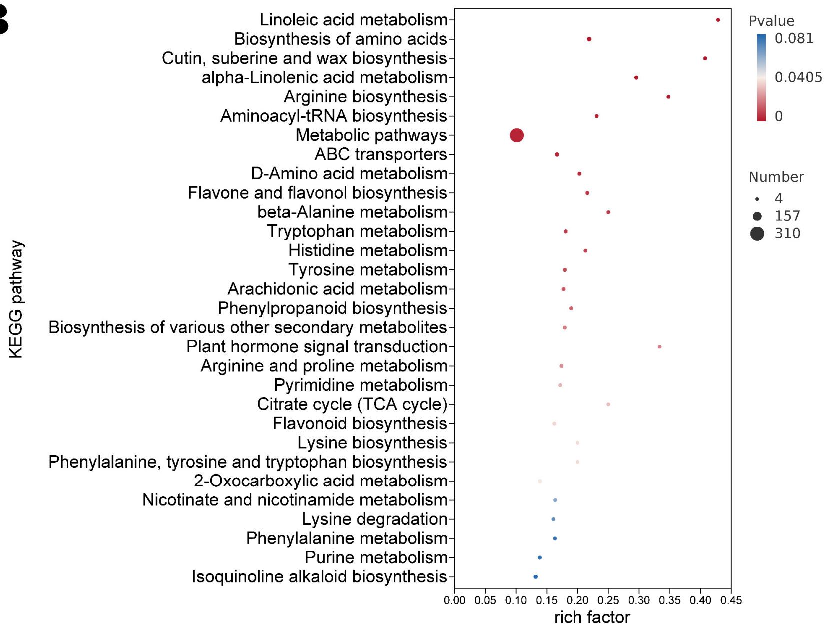

Figure S17 (A) KEGG enrichment bar plot among HB, HB-1, HB-2, and HB-3; (B) KEGG enrichment factor plot among HB, HB-1, HB-2, and HB-3.
