# Supplementary material for: Chemical Composition Analysis of Highland Barley (Hordeum vulgare L.) with Different Modification Methods and Lipid Metabolism Mechanism Analysis of Highland Barley with Microwave Fluidization Modification
Source: Foods. 2026 Apr 17;15(8):1396. doi: 10.3390/foods15081396 (PMC13114515; doi:10.3390/foods15081396)
Supplement: Supplementary file 1 [file foods-15-01396-s001.zip › Figure S18.pdf]

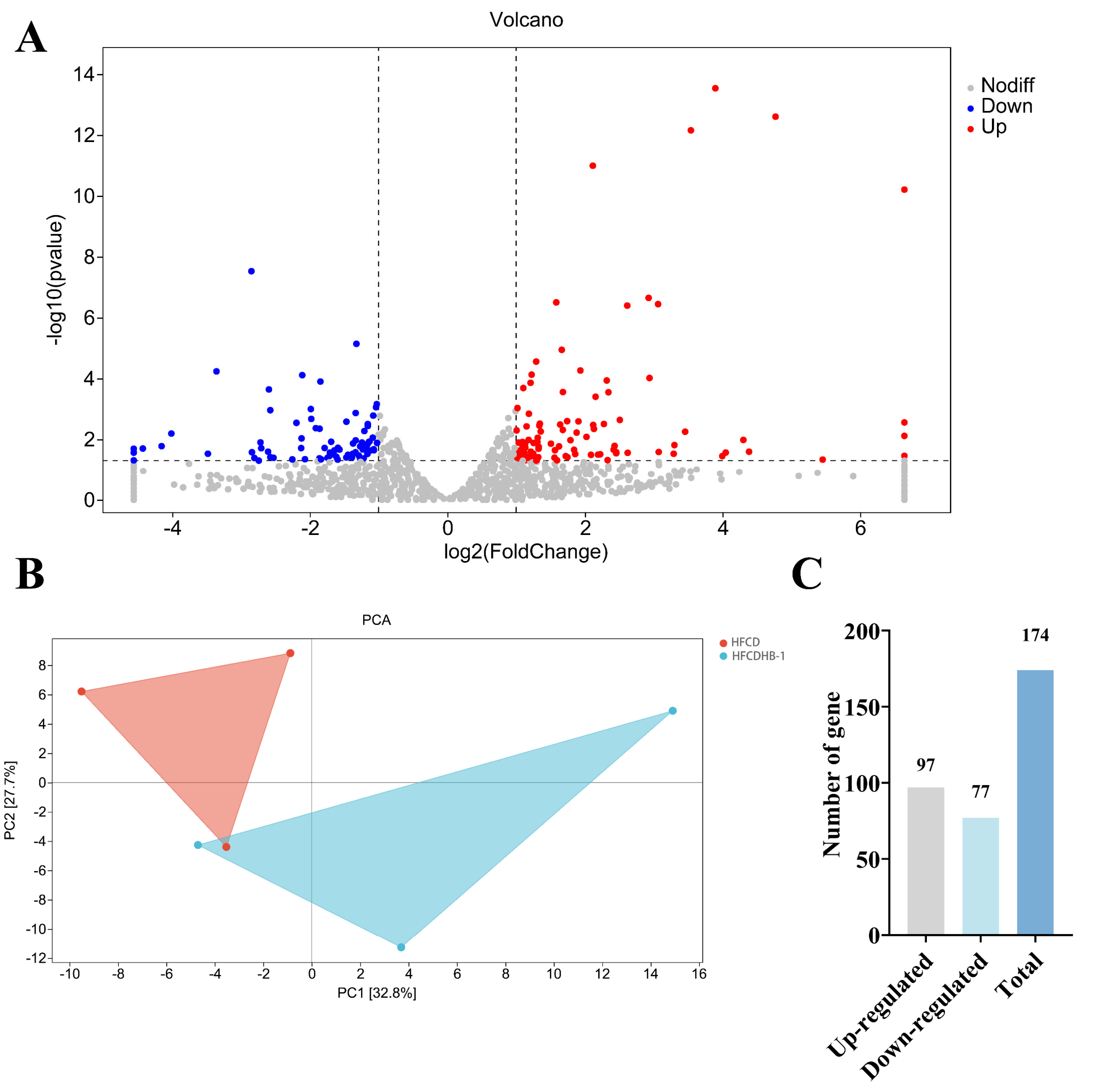

Figure S18 Liver transcriptomic analysis between HFCD and HFCD+HB-1. (A) Volcano plot; (B) HFCD and HFCD+HB-1 PCA plot; (C) Up-regulated and down-regulated differential genes.
