# Supplementary material for: Chemical Composition Analysis of Highland Barley (Hordeum vulgare L.) with Different Modification Methods and Lipid Metabolism Mechanism Analysis of Highland Barley with Microwave Fluidization Modification
Source: Foods. 2026 Apr 17;15(8):1396. doi: 10.3390/foods15081396 (PMC13114515; doi:10.3390/foods15081396)
Supplement: Supplementary file 1 [file foods-15-01396-s001.zip › Figure S2.pdf]

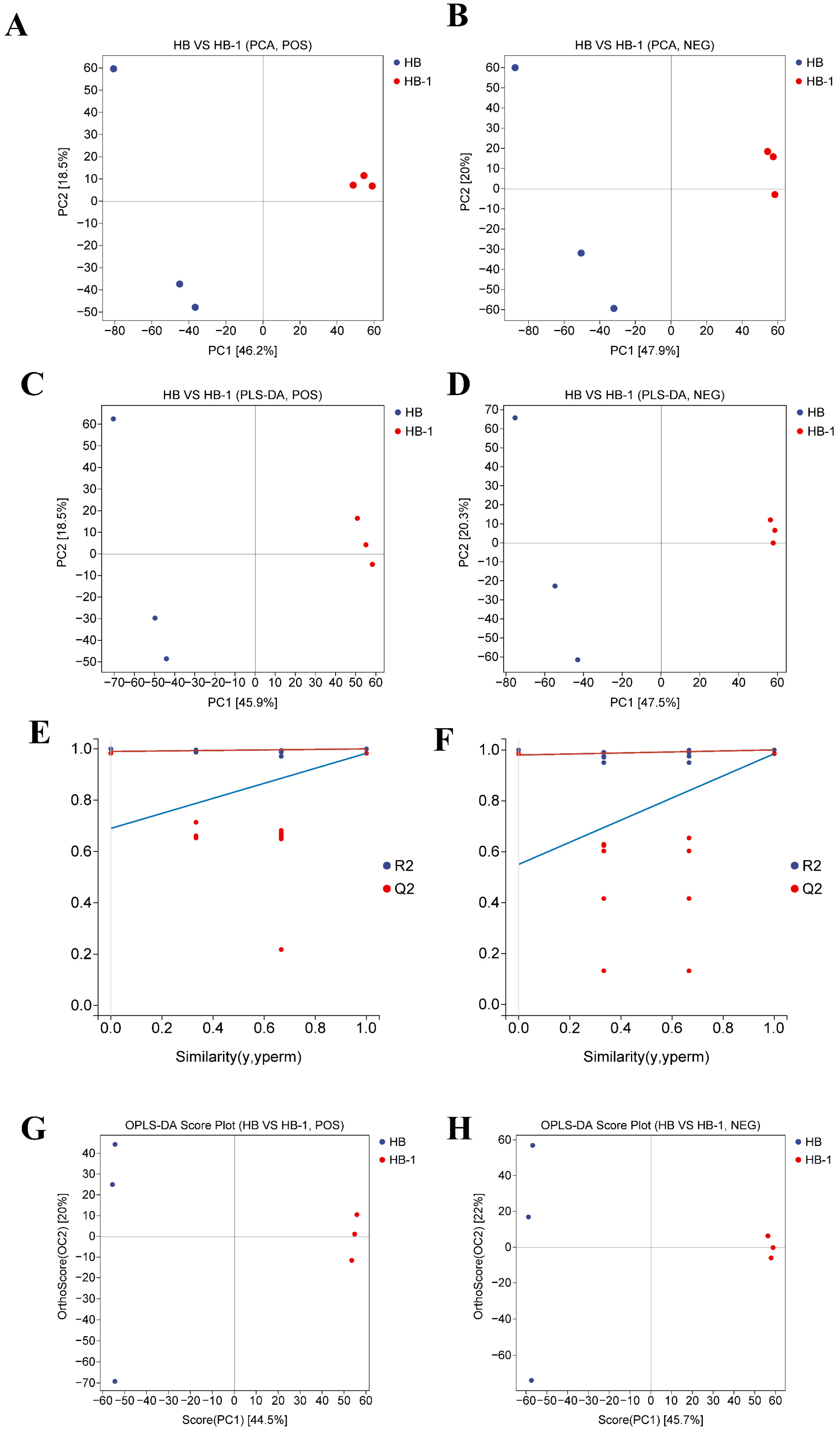

A
HB VS HB-1 (PCA, POS)
HB
HB-1
PC2 [18.5%]
PC1 [46.2%]
B
HB VS HB-1 (PCA, NEG)
PC2 [20%]
PC1 [47.9%]
C
HB VS HB-1 (PLS-DA, POS)
PC2 [18.5%]
PC1 [45.9%]
D
HB VS HB-1 (PLS-DA, NEG)
PC2 [20.3%]
PC1 [47.5%]
E
R2
Q2
Similarity(y,yperm)
F
R2
Q2
Similarity(y,yperm)
G
OPLS-DA Score Plot (HB VS HB-1, POS)
OrthoScore(OC2) [20%]
Score(PC1) [44.5%]
H
OPLS-DA Score Plot (HB VS HB-1, NEG)
OrthoScore(OC2) [22%]
Score(PC1) [45.7%]

Figure S2 Multivariate statistical analysis of HB VS HB-1. (A) PCA plot in LC-ESI (+)-MS; (B) PCA plot in LC-ESI (-)-MS; (C and E) PLS-DA plot in LC-ESI (+)-MS; (D and F) PLS-DA plot in LC-ESI (-)-MS; (G) OPLS-DA plot in LC-ESI (+)-MS; (H) OPLS-DA plot in LC-ESI (-)-MS.
