# Supplementary material for: Chemical Composition Analysis of Highland Barley (Hordeum vulgare L.) with Different Modification Methods and Lipid Metabolism Mechanism Analysis of Highland Barley with Microwave Fluidization Modification
Source: Foods. 2026 Apr 17;15(8):1396. doi: 10.3390/foods15081396 (PMC13114515; doi:10.3390/foods15081396)
Supplement: Supplementary file 1 [file foods-15-01396-s001.zip › Figure S3.pdf]

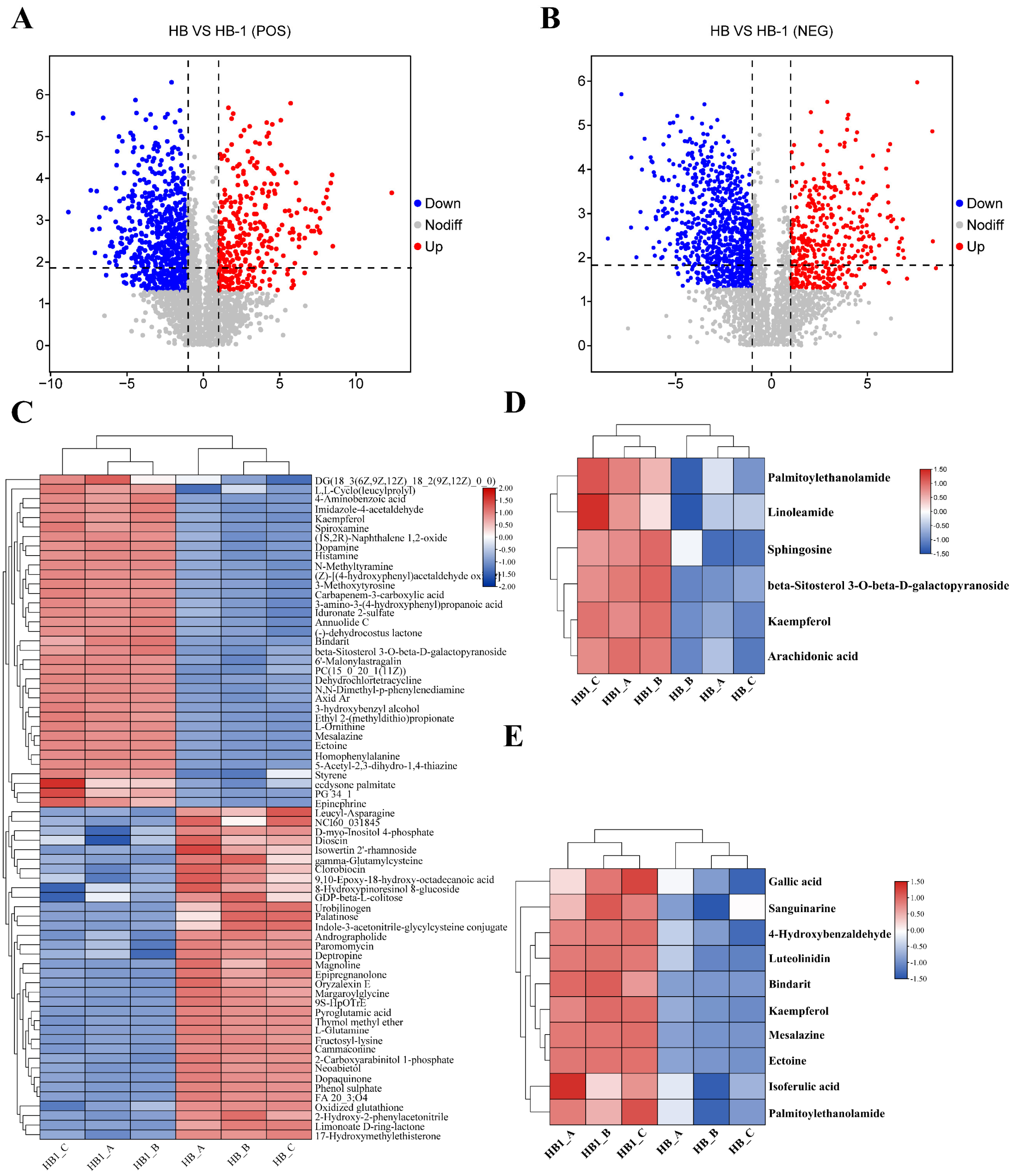

Figure S3 Differential metabolites result between HB and HB-1. (A) Volcano plot in positive mode; (B) Volcano plot in negative mode; (C) Significantly differential metabolites between HB and HB-1; (D) Main metabolites related to lipid metabolism; (E) Main metabolites related to anti-inflammation.
