# Supplementary material for: Chemical Composition Analysis of Highland Barley (Hordeum vulgare L.) with Different Modification Methods and Lipid Metabolism Mechanism Analysis of Highland Barley with Microwave Fluidization Modification
Source: Foods. 2026 Apr 17;15(8):1396. doi: 10.3390/foods15081396 (PMC13114515; doi:10.3390/foods15081396)
Supplement: Supplementary file 1 [file foods-15-01396-s001.zip › Figure S4.pdf]

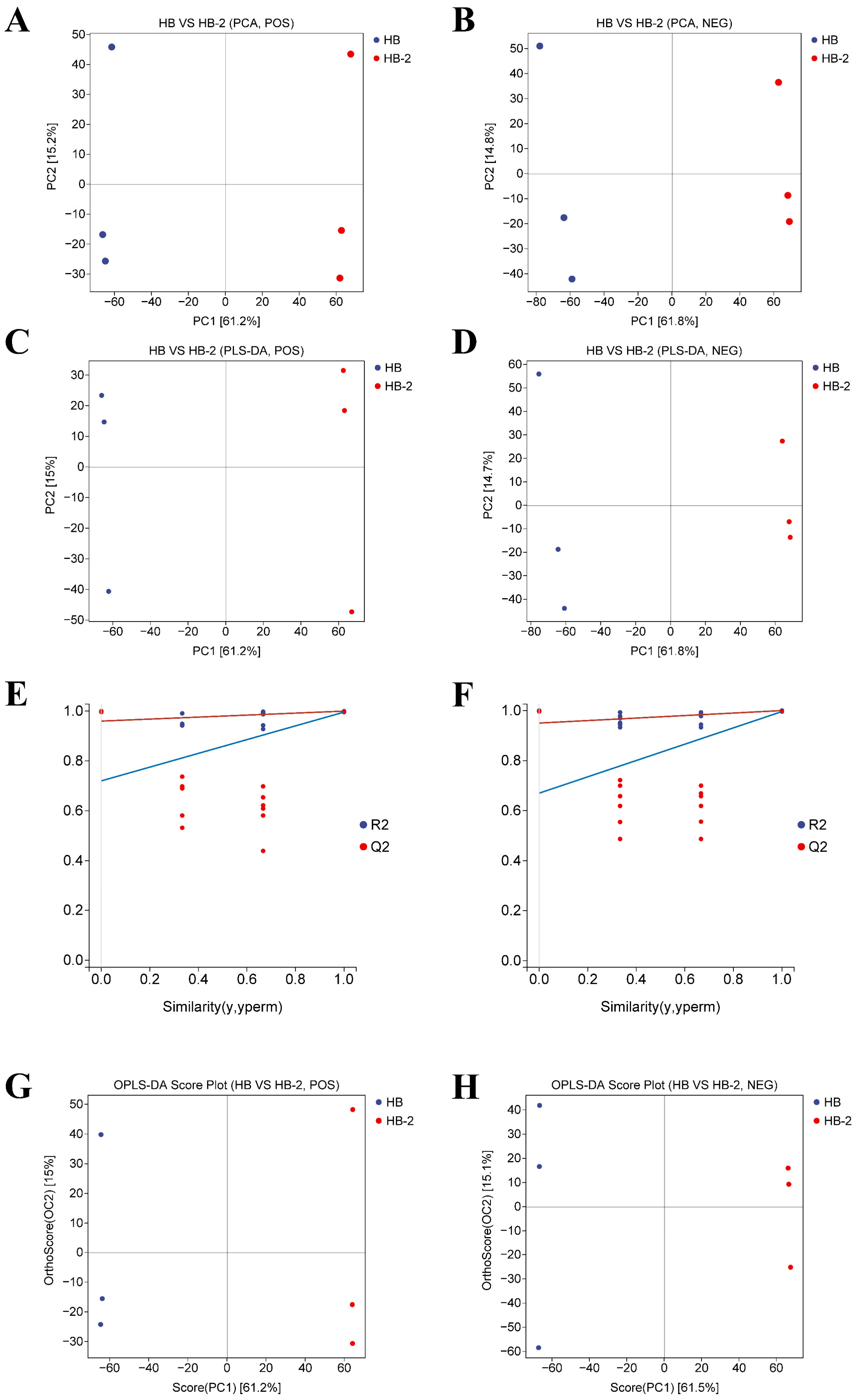
A
HB VS HB-2 (PCA, POS)
HB
HB-2
PC2 [15.2%]
PC1 [61.2%]
B
HB VS HB-2 (PCA, NEG)
HB
HB-2
PC2 [14.8%]
PC1 [61.8%]
C
HB VS HB-2 (PLS-DA, POS)
HB
HB-2
PC2 [15%]
PC1 [61.2%]
D
HB VS HB-2 (PLS-DA, NEG)
HB
HB-2
PC2 [14.7%]
PC1 [61.8%]
E
R2
Q2
Similarity(y,yperm)
F
R2
Q2
Similarity(y,yperm)
G
OPLS-DA Score Plot (HB VS HB-2, POS)
HB
HB-2
OrthoScore(OC2) [15%]
Score(PC1) [61.2%]
H
OPLS-DA Score Plot (HB VS HB-2, NEG)
HB
HB-2
OrthoScore(OC2) [15.1%]
Score(PC1) [61.5%]

Figure S4 Multivariate statistical analysis of HB VS HB-2. (A) PCA plot in LC-ESI (+)-MS; (B) PCA plot in LC-ESI (-)-MS; (C and E) PLS-DA plot in LC-ESI (+)-MS; (D and F) PLS-DA plot in LC-ESI (-)-MS; (G) OPLS-DA plot in LC-ESI (+)-MS; (H) OPLS-DA plot in LC-ESI (-)-MS.
