# Supplementary material for: Chemical Composition Analysis of Highland Barley (Hordeum vulgare L.) with Different Modification Methods and Lipid Metabolism Mechanism Analysis of Highland Barley with Microwave Fluidization Modification
Source: Foods. 2026 Apr 17;15(8):1396. doi: 10.3390/foods15081396 (PMC13114515; doi:10.3390/foods15081396)
Supplement: Supplementary file 1 [file foods-15-01396-s001.zip › Figure S5.pdf]

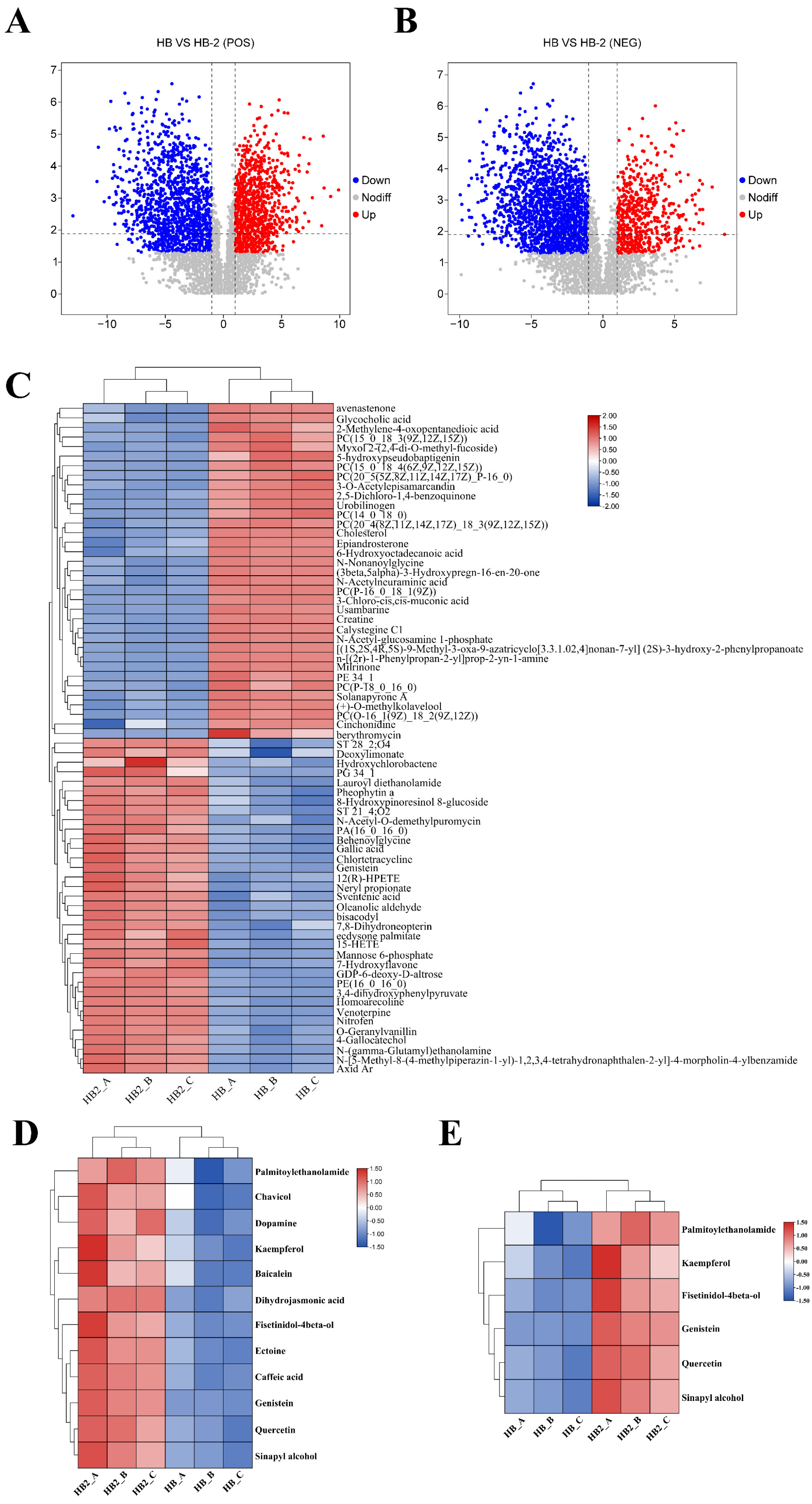
A
HB VS HB-2 (POS)
Down
Nodiff
Up
B
HB VS HB-2 (NEG)
Down
Nodiff
Up
C
avenastenone
Glycocholic acid
2-Methylene-4-oxopentanedioic acid
PC(15_0_18_3(9Z,12Z,15Z))
Myxol 2-(2,4-di-O-methyl-fucoside)
5-hydroxypseudobaptigenin
PC(15_0_18_4(6Z,9Z,12Z,15Z))
PC(20_5(5Z,8Z,11Z,14Z,17Z)_P-16_0)
3-O-Acetylepisamarcandin
2,5-Dichloro-1,4-benzoquinone
Urobilinogen
PC(14_0_18_0)
PC(20_4(8Z,11Z,14Z,17Z)_18_3(9Z,12Z,15Z))
Cholesterol
Epiandrosterone
6-Hydroxyoctadecanoic acid
N-Nonanoylglycine
(3beta,5alpha)-3-Hydroxypregn-16-en-20-one
N-Acetylneuraminic acid
PC(P-16_0_18_1(9Z))
3-Chloro-cis,cis-muconic acid
Usambarine
Creatine
Calystegine C1
N-Acetyl-glucosamine 1-phosphate
[(1S,2S,4R,5S)-9-Methyl-3-oxa-9-azatricyclo[3.3.1.02,4]nonan-7-yl] (2S)-3-hydroxy-2-phenylpropanoate
n-[(2r)-1-Phenylpropan-2-yl]prop-2-yn-1-amine
Milrinone
PE 34_1
PC(P-18_0_16_0)
Solanapyrone A
(+)-O-methylkolavelool
PC(O-16_1(9Z)_18_2(9Z,12Z))
Cinchonidine
berythromycin
ST 28_2;O4
Deoxylimonate
Hydroxychlorobactene
PG 34_1
Lauroyl diethanolamide
Pheophytin a
8-Hydroxypinoresinol 8-glucoside
ST 21_4;O2
N-Acetyl-O-demethylpuromycin
PA(16_0_16_0)
Behenoylglycine
Gallic acid
Chlortetracycline
Genistein
12(R)-HPETE
Neryl propionate
Sventenic acid
Oleanolic aldehyde
bisacodyl
7,8-Dihydroneopterin
ecdysone palmitate
15-HETE
Mannose 6-phosphate
7-Hydroxyflavone
GDP-6-deoxy-D-altrose
PE(16_0_16_0)
3,4-dihydroxyphenylpyruvate
Homoarecoline
Venoterpine
Nitrofen
O-Geranylvanillin
4-Gallocatechol
N-(gamma-Glutamyl)ethanolamine
N-[5-Methyl-8-(4-methylpiperazin-1-yl)-1,2,3,4-tetrahydronaphthalen-2-yl]-4-morpholin-4-ylbenzamide
Axid Ar
HB2_A
HB2_B
HB2_C
HB_A
HB_B
HB_C
D
Palmitoylethanolamide
Chavicol
Dopamine
Kaempferol
Baicalein
Dihydrojasmonic acid
Fisetinidol-4beta-ol
Ectoine
Caffeic acid
Genistein
Quercetin
Sinapyl alcohol
HB2_A
HB2_B
HB2_C
HB_A
HB_B
HB_C
E
Palmitoylethanolamide
Kaempferol
Fisetinidol-4beta-ol
Genistein
Quercetin
Sinapyl alcohol
HB_A
HB_B
HB_C
HB2_A
HB2_B
HB2_C

Figure S5 Differential metabolites result between HB and HB-2. (A) Volcano plot in positive mode; (B) Volcano plot in negative mode; (C) Significantly differential metabolites between HB and HB-1; (D) Main metabolites related to anti-inflammation; (E) Main metabolites related to lipid metabolism.
