# Supplementary material for: Chemical Composition Analysis of Highland Barley (Hordeum vulgare L.) with Different Modification Methods and Lipid Metabolism Mechanism Analysis of Highland Barley with Microwave Fluidization Modification
Source: Foods. 2026 Apr 17;15(8):1396. doi: 10.3390/foods15081396 (PMC13114515; doi:10.3390/foods15081396)
Supplement: Supplementary file 1 [file foods-15-01396-s001.zip › Figure S6.pdf]

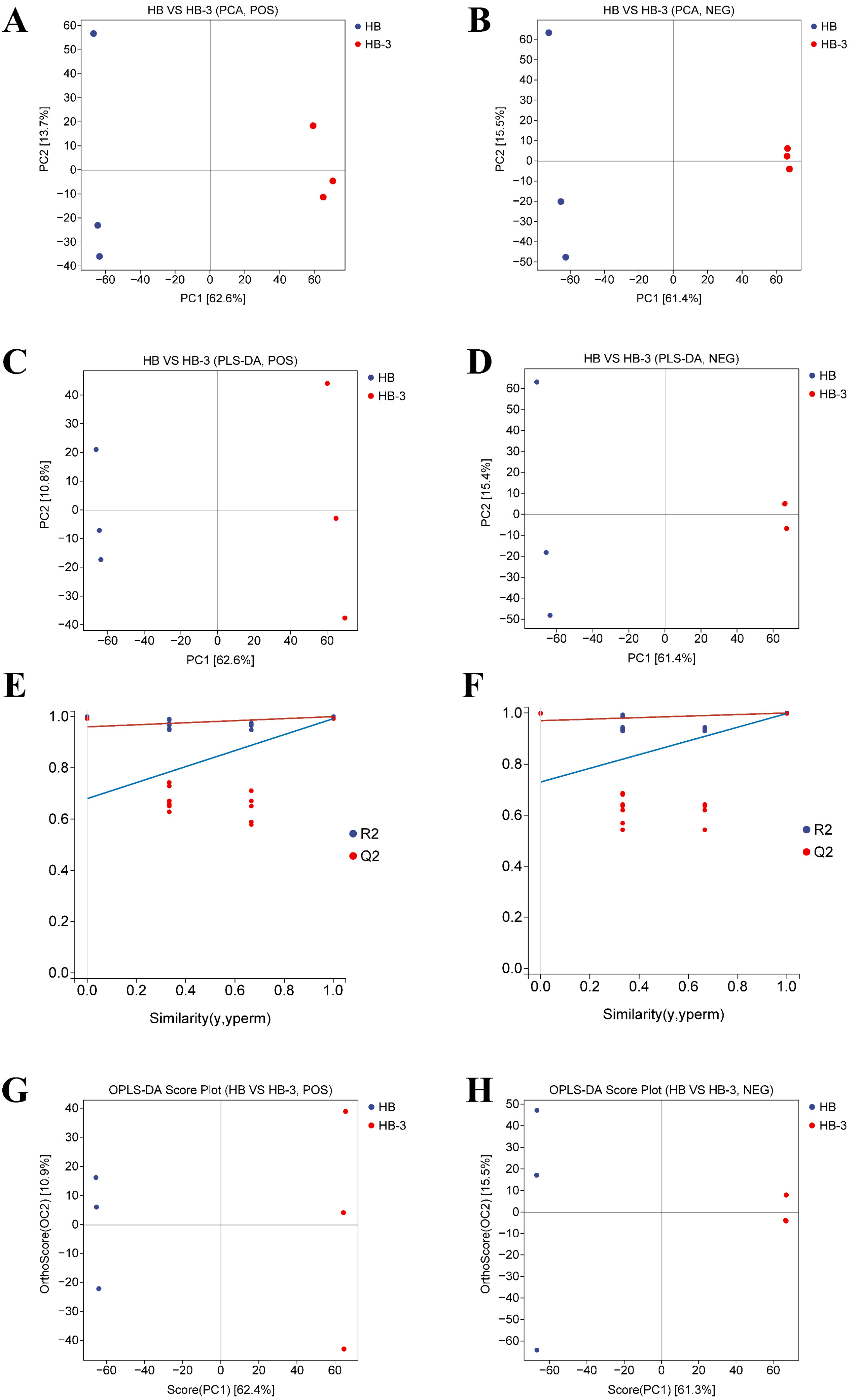
A
HB VS HB-3 (PCA, POS)
PC1 [62.6%]
PC2 [13.7%]
HB
HB-3
B
HB VS HB-3 (PCA, NEG)
PC1 [61.4%]
PC2 [15.5%]
HB
HB-3
C
HB VS HB-3 (PLS-DA, POS)
PC1 [62.6%]
PC2 [10.8%]
HB
HB-3
D
HB VS HB-3 (PLS-DA, NEG)
PC1 [61.4%]
PC2 [15.4%]
HB
HB-3
E
Similarity(y,yperm)
R2
Q2
F
Similarity(y,yperm)
R2
Q2
G
OPLS-DA Score Plot (HB VS HB-3, POS)
Score(PC1) [62.4%]
OrthoScore(OC2) [10.9%]
HB
HB-3
H
OPLS-DA Score Plot (HB VS HB-3, NEG)
Score(PC1) [61.3%]
OrthoScore(OC2) [15.5%]
HB
HB-3

Figure S6 Multivariate statistical analysis of HB VS HB-3. (A) PCA plot in LC-ESI (+)-MS; (B) PCA plot in LC-ESI (-)-MS; (C and E) PLS-DA plot in LC-ESI (+)-MS; (D and F) PLS-DA plot in LC-ESI (-)-MS; (G) OPLS-DA plot in LC-ESI (+)-MS; (H) OPLS-DA plot in LC-ESI (-)-MS.
