# Supplementary material for: Chemical Composition Analysis of Highland Barley (Hordeum vulgare L.) with Different Modification Methods and Lipid Metabolism Mechanism Analysis of Highland Barley with Microwave Fluidization Modification
Source: Foods. 2026 Apr 17;15(8):1396. doi: 10.3390/foods15081396 (PMC13114515; doi:10.3390/foods15081396)
Supplement: Supplementary file 1 [file foods-15-01396-s001.zip › Table S1.pdf]

Table S1 Mouse basic diet composition and nutrition level.

Compositions	NCD		HFCD		HFCD+HB-1		HFCD+HB-2		HFCD+HB-3	
	g%	kcal%	g%	kcal%	g%	kcal%	g%	kcal%	g%	kcal%
Protein	19	20	22.62	19.97	22.62	19.97	22.62	19.97	22.62	19.97
Carbohydrate	67	70	45.51	40.18	45.51	40.18	45.51	40.18	45.51	40.18
Fat	4	10	20.06	39.85	20.06	39.85	20.06	39.85	20.06	39.85
Total	/	100	/	100	/	100	/	100	/	100
kcal/g	3.85	/	4.53	/	4.53	/	4.53	/	4.53	/
**Ingredients**	gm	kcal	gm	kcal	gm	kcal	gm	kcal	gm	kcal
Casein, 80 Mesh	200	800	200	800	148	592	120	480	87	348
L-Cystine	3	12	3	12	3	12	3	12	3	12
Corn Starch	386.15	1544.6	212	848	0	0	0	0	0	0
Maltodextrin	125	500	71	284	0	0	0	0	0	0
Sucrose	200	800	124.41	497.64	124.41	497.64	124.41	497.64	124.41	497.64
Sample Fat	0	0	0	0	11	99	12	108	28	252
Sample Protein	0	0	0	0	52	208	80	320	113	452
Sample starch	0	0	0	0	283	1132	283	1132	283	1132
Cellulose	50	0	50	0	50	0	50	0	50	0
Soybean Oil	25	225	25	225	14	126	13	117	0	0
Lard	20	180	155	1395	155	1395	155	1395	152	1368
Mineral Mix S10020	5	0	5	0	5	0	5	0	5	0
Calcium Phosphate	13	0	13	0	13	0	13	0	13	0
Calcium Carbonate	5.5	0	5.5	0	5.5	0	5.5	0	5.5	0
Potassium Citrate,1H_2O	16.5	0	16.5	0	16.5	0	16.5	0	16.5	0
Sodium Chloride	2.59	0	2.59	0	2.59	0	2.59	0	2.59	0

Vitamin Mix V10001C	1	4	1	4	1	4	1	4	1	4
Choline Bitartrate	2	0	2	0	2	0	2	0	2	0
Cholesterol	0	0	11.25	0	11.25	0	11.25	0	11.25	0
Total	1053.84	4065.6	896.35	4065.64	896.35	4065.64	896.35	4065.64	896.35	4065.64

HB-1 accounted for 43% of the total; HB-2 accounted for 50% of the total; and HB-3 accounted for 54% of the total.

Normal control group (NCD, AIN-93M diet).

XT108C HFCD group (HFCD).

Highland barley group (HFCD+HB, XT108C HFCD + Highland barley).

Fluidized highland barley group (HFCD+HB-1, XT108C HFCD + Microwave fluidized highland barley).

Extruded and puffed highland barley group (HFCD+HB-2, XT108C HFCD + Extruded and puffed highland barley).

Ultrafine pulverized highland barley group (HFCD+HB-3, XT108C HFCD + Ultrafine pulverized highland barley).
