# Supplementary material for: Chemical Composition Analysis of Highland Barley (Hordeum vulgare L.) with Different Modification Methods and Lipid Metabolism Mechanism Analysis of Highland Barley with Microwave Fluidization Modification
Source: Foods. 2026 Apr 17;15(8):1396. doi: 10.3390/foods15081396 (PMC13114515; doi:10.3390/foods15081396)
Supplement: Supplementary file 1 [file foods-15-01396-s001.zip › Table S10.pdf]

Table S10 The top 50 significant KEGG pathways among HB, HB-1, HB-2 and HB-3.

PathwayID	Pathway	level1	level2	DEM	Total	Pvalue	FDR
map00591	Linoleic acid metabolism	Metabolism	Lipid metabolism	12	28	2.44E-06	0.000254
map01230	Biosynthesis of amino acids	Metabolism	Global and overview maps	28	128	9.16E-06	0.000407
map00073	Cutin, suberine and wax biosynthesis	Metabolism	Lipid metabolism	11	27	1.17E-05	0.000407
map00592	alpha-Linolenic acid metabolism	Metabolism	Lipid metabolism	13	44	0.000102	0.002654
map00220	Arginine biosynthesis	Metabolism	Amino acid metabolism	8	23	0.000678	0.0141
map00970	Aminoacyl-tRNA biosynthesis	Genetic Information Processing	Translation	12	52	0.002136	0.032616
map01100	Metabolic pathways	Metabolism	Global and overview maps	310	3063	0.002195	0.032616
map02010	ABC transporters	Environmental Information Processing	Membrane transport	23	138	0.003312	0.03966
map00470	D-Amino acid metabolism	Metabolism	Metabolism of other amino acids	14	69	0.003432	0.03966
map00944	Flavone and flavonol biosynthesis	Metabolism	Biosynthesis of other secondary metabolites	11	51	0.005625	0.058504
map00410	beta-Alanine metabolism	Metabolism	Metabolism of other amino acids	8	32	0.00695	0.065709
map00380	Tryptophan metabolism	Metabolism	Amino acid metabolism	15	83	0.007775	0.067383
map00340	Histidine metabolism	Metabolism	Amino acid metabolism	10	47	0.008989	0.071913
map00350	Tyrosine metabolism	Metabolism	Amino acid metabolism	14	78	0.010575	0.078557
map00590	Arachidonic acid metabolism	Metabolism	Lipid metabolism	14	79	0.011809	0.081873
map00940	Phenylpropanoid	Metabolism	Biosynthesis of other	11	58	0.01499	0.097434

	biosynthesis		secondary metabolites				
map00997	Biosynthesis of various other secondary metabolites	Metabolism	Biosynthesis of other secondary metabolites	12	67	0.017565	0.107455
map04075	Plant hormone signal transduction	Environmental Information Processing	Signal transduction	4	12	0.019181	0.110825
map00330	Arginine and proline metabolism	Metabolism	Amino acid metabolism	12	69	0.021846	0.119578
map00240	Pyrimidine metabolism	Metabolism	Nucleotide metabolism	11	64	0.02976	0.154373
map00020	Citrate cycle (TCA cycle)	Metabolism	Carbohydrate metabolism	5	20	0.031172	0.154373
map00941	Flavonoid biosynthesis	Metabolism	Biosynthesis of other secondary metabolites	12	74	0.035865	0.160278
map00300	Lysine biosynthesis	Metabolism	Amino acid metabolism	7	35	0.036987	0.160278
map00400	Phenylalanine, tyrosine and tryptophan biosynthesis	Metabolism	Amino acid metabolism	7	35	0.036987	0.160278
map01210	2-Oxocarboxylic acid metabolism	Metabolism	Global and overview maps	20	144	0.038979	0.162152
map00760	Nicotinate and nicotinamide metabolism	Metabolism	Metabolism of cofactors and vitamins	9	55	0.061185	0.244741
map00310	Lysine degradation	Metabolism	Amino acid metabolism	9	56	0.067269	0.25911
map00360	Phenylalanine metabolism	Metabolism	Amino acid metabolism	8	49	0.075929	0.275387
map00230	Purine metabolism	Metabolism	Nucleotide metabolism	14	101	0.076791	0.275387
map00950	Isoquinoline alkaloid biosynthesis	Metabolism	Biosynthesis of other secondary metabolites	17	129	0.080681	0.279695
map00660	C5-Branched dibasic acid metabolism	Metabolism	Carbohydrate metabolism	6	35	0.096552	0.323916
map00904	Diterpenoid biosynthesis	Metabolism	Metabolism of terpenoids and	16	124	0.101921	0.331242

			polyketides				
map00250	Alanine, aspartate and glutamate metabolism	Metabolism	Amino acid metabolism	5	28	0.108448	0.341774
map00460	Cyanoamino acid metabolism	Metabolism	Metabolism of other amino acids	7	45	0.114188	0.34928
map00750	Vitamin B6 metabolism	Metabolism	Metabolism of cofactors and vitamins	5	29	0.121709	0.351604
map00905	Brassinosteroid biosynthesis	Metabolism	Metabolism of terpenoids and polyketides	5	29	0.121709	0.351604
map00480	Glutathione metabolism	Metabolism	Metabolism of other amino acids	6	38	0.130798	0.36765
map00770	Pantothenate and CoA biosynthesis	Metabolism	Metabolism of cofactors and vitamins	5	30	0.135686	0.368098
map01040	Biosynthesis of unsaturated fatty acids	Metabolism	Lipid metabolism	10	74	0.138037	0.368098
map00261	Monobactam biosynthesis	Metabolism	Biosynthesis of other secondary metabolites	6	39	0.143353	0.372719
map00260	Glycine, serine and threonine metabolism	Metabolism	Amino acid metabolism	7	48	0.147049	0.373003
map00965	Betalain biosynthesis	Metabolism	Biosynthesis of other secondary metabolites	4	23	0.155102	0.382683
map00270	Cysteine and methionine metabolism	Metabolism	Amino acid metabolism	9	67	0.158225	0.382683
map00332	Carbapenem biosynthesis	Metabolism	Biosynthesis of other secondary metabolites	5	32	0.165621	0.391468
map01110	Biosynthesis of secondary metabolites	Metabolism	Global and overview maps	218	2273	0.198903	0.459688
map00943	Isoflavonoid biosynthesis	Metabolism	Biosynthesis of other	8	64	0.231385	0.523132

			secondary metabolites				
map00052	Galactose metabolism	Metabolism	Carbohydrate metabolism	6	46	0.244374	0.540662
map01240	Biosynthesis of cofactors	Metabolism	Global and overview maps	34	328	0.249536	0.540662
map00903	Limonene degradation	Metabolism	Metabolism of terpenoids and polyketides	6	47	0.260288	0.552447
map00100	Steroid biosynthesis	Metabolism	Lipid metabolism	7	57	0.266921	0.555196

Total, the total number of metabolites in the target metabolic pathway;

Pvalue, the p value of the hypergeometric distribution test;

FDR, corrected for false positives;

Pathway, metabolite metabolism pathway ID.
