# Supplementary material for: Chemical Composition Analysis of Highland Barley (Hordeum vulgare L.) with Different Modification Methods and Lipid Metabolism Mechanism Analysis of Highland Barley with Microwave Fluidization Modification
Source: Foods. 2026 Apr 17;15(8):1396. doi: 10.3390/foods15081396 (PMC13114515; doi:10.3390/foods15081396)
Supplement: Supplementary file 1 [file foods-15-01396-s001.zip › Table S11.pdf]

Table S11 The top 50 differential genes analysis between HFCD and HFCD+HB-1.

**Name**	**baseMean**	**baseMean_HFCD**	**baseMean_HFCD+HB-1**	**foldChange(HFCD+HB-1/HFCD)(HFCD+HB-1/HFCD)**	**log2FoldChange**	**pval**	**padj**	**KEGG**
Themis	45.706144	5.7548869	85.657402	14.884289	3.8957184	2.91E-14	4.79E-10	-
Cyp2b13	41.301234	2.9191009	79.683367	27.297229	4.7706826	2.49E-13	2.05E-09	K07412
Cyp2a22	55.470547	8.7817248	102.15937	11.633178	3.5401734	7.03E-13	3.85E-09	-
Apoa4	486.12085	182.37563	789.86607	4.3309846	2.1146951	1.02E-11	4.20E-08	K08760
Padi4	16.832078	0.3333229	33.330833	99.995626	6.6437931	6.23E-11	2.05E-07	K24669
Dnai1	44.945972	78.932065	10.959878	0.138852	-2.84838	2.99E-08	8.18E-05	K10409
Bmp7	27.967355	6.5048477	49.429863	7.598927	2.9257957	2.25E-07	0.0005282	K16621
Gpat3	340.97142	170.63115	511.31169	2.9965905	1.5833219	3.15E-07	0.0006483	K13506
Mogat1	25.349853	5.4174191	45.282287	8.3586456	3.0632692	3.60E-07	0.000658	K14458
Lrtm1	57.207043	16.055873	98.358212	6.125996	2.6149444	4.03E-07	0.0006629	-
Cyp2c68	1162.4647	1660.7391	664.19034	0.3999366	-1.322157	7.27E-06	0.0108651	K07413
Stap1	88.39448	42.427041	134.36193	3.1668938	1.6630685	1.14E-05	0.015674	K24025

	7						2	
Cyp2d40	405.12559	235.03692	575.21427	2.4473358	1.2912121	2.78E-05	0.0352284	K07414
Tlr12	56.894075	23.604451	90.1837	3.8206227	1.9338078	5.47E-05	0.064055	K18807
Cfap53	42.938958	78.237863	7.6400534	0.0976516	-3.356212	5.84E-05	0.064055	K24225
Wwtr1	365.88899	219.24574	512.53223	2.3377066	1.2250939	7.47E-05	0.0751243	K16820
E2f7	30.20028	49.036429	11.364131	0.2317488	-2.109367	7.77E-05	0.0751243	K09391
Cyp2b9	260.62511	60.139791	461.11043	7.6673102	2.9387205	9.63E-05	0.0879785	K07412
Serpine1	27.071182	9.0494371	45.092926	4.9829537	2.3170012	0.0001162	0.1006081	K03982
Wnk4	60.640364	94.876547	26.404182	0.2783004	-1.845285	0.0001262	0.1037676	K08867
Mpeg1	1072.2595	647.33453	1497.1845	2.3128451	1.2096686	0.0001389	0.108802	-
Gda	727.20471	461.7381	992.67133	2.149858	1.1042413	0.0002059	0.1539469	K01487
Adgrf1	14.392318	24.694889	4.0897472	0.1656111	-2.594129	0.0002291	0.1637832	K08453
Mfsd2a	39.475716	18.780715	60.170718	3.2038567	1.6798096	0.0002784	0.1866833	K23894
Slc35f2	18.138763	5.9784004	30.299125	5.0680989	2.3414447	0.0002838	0.1866833	K15287
Morc4	19.712409	7.2308868	32.193931	4.4522797	2.1545442	0.0003989	0.2523004	K24135

Slco2a1	592.84955	795.23672	390.46237	0.4910014	-1.026201	0.0006961	0.4239994	K14345
Stard4	477.9972	642.76577	313.22863	0.4873138	-1.037077	0.0008788	0.5161966	-
Tspan4	368.82066	243.6895	493.95181	2.0269721	1.0193262	0.0009362	0.5309087	K17294
Hectd2	41.023737	65.493495	16.553979	0.2527576	-1.984174	0.0010093	0.5532776	K12232
Cplane1	466.11974	798.19608	134.0434	0.1679329	-2.574043	0.0011008	0.5840058	K22859
Ermp1	613.22705	410.27945	816.17466	1.989314	0.992271	0.0011872	0.6101268	-
Grm8	137.95016	197.49777	78.402552	0.3969794	-1.332864	0.0013621	0.6788113	K04610
Tenm3	183.85822	112.32876	255.38768	2.2735735	1.1849616	0.0014401	0.6965909	K24473
Elovl3	2512.0444	3408.7055	1615.3833	0.4738993	-1.077347	0.0016563	0.7731953	K10248
Rint1	291.95995	387.61149	196.30841	0.5064566	-0.981489	0.0016925	0.7731953	K20474
Ddah1	1592.3999	1118.1999	2066.6	1.8481489	0.886081	0.0019945	0.886521	K01482
Clic3	16.956385	27.048177	6.8645937	0.2537914	-1.978285	0.0021293	0.9215597	K05023
Obp2a	677.16696	202.59573	1151.7382	5.6849086	2.5071372	0.0023017	0.9705948	K25352
Wdr62	24.216076	11.150894	37.281258	3.3433424	1.7412911	0.0025215	1	K21762
Tox	32.51839	13.702836	51.33396	3.746229	1.9054391	0.002564	1	-

	8					2		
Rpl35a	366.34617	538.10964	194.58269	0.3616042	-1.467517	0.0026308	1	K02917
Ren1	3.4491953	0	6.8983905	Inf	Inf	0.0027829	1	K01380
Pitx3	12.628732	20.727226	4.5302375	0.2185646	-2.193868	0.0028685	1	K09357
Tnfrsf19	65.691578	37.125202	94.257955	2.538921	1.3442155	0.0030528	1	K05155
Ptk7	11.628018	3.9805354	19.2755	4.842439	2.2757339	0.0031247	1	K05127
Wsb1	886.73695	1224.3141	549.15977	0.4485448	-1.156676	0.0031489	1	K10341
Slc22a3	62.441085	30.335822	94.546347	3.1166569	1.6399993	0.0032626	1	K08200
Tchh	16.170982	6.0488434	26.29312	4.3468012	2.1199541	0.0033647	1	K18626
Hpgd	868.9576	492.8438	1245.0714	2.5263002	1.3370261	0.0035145	1	K00069
