# Supplementary material for: Chemical Composition Analysis of Highland Barley (Hordeum vulgare L.) with Different Modification Methods and Lipid Metabolism Mechanism Analysis of Highland Barley with Microwave Fluidization Modification
Source: Foods. 2026 Apr 17;15(8):1396. doi: 10.3390/foods15081396 (PMC13114515; doi:10.3390/foods15081396)
Supplement: Supplementary file 1 [file foods-15-01396-s001.zip › Table S12.pdf]

Table S12 The top 50 significant GO pathways between HFCD and HFCD+HB-1.

GO.ID	Category	Term	Up	Down	DEG	Total	Pvalue	adjustPvalue
GO:1901700	BP	response to oxygen-containing compound	28	12	40	1845	6.70E-09	1.15E-05
GO:0019217	BP	regulation of fatty acid metabolic process	7	3	10	102	1.09E-08	1.15E-05
GO:0032787	BP	monocarboxylic acid metabolic process	15	8	23	696	1.18E-08	1.15E-05
GO:1901701	BP	cellular response to oxygen-containing compound	22	10	32	1289	1.32E-08	1.15E-05
GO:0042180	BP	cellular ketone metabolic process	10	4	14	245	1.40E-08	1.15E-05
GO:0070887	BP	cellular response to chemical stimulus	32	23	55	3205	2.05E-08	1.40E-05
GO:0006631	BP	fatty acid metabolic process	12	6	18	463	4.64E-08	2.72E-05
GO:0010565	BP	regulation of cellular ketone metabolic process	7	4	11	157	6.68E-08	3.43E-05
GO:0019752	BP	carboxylic acid metabolic process	16	11	27	1035	7.91E-08	3.61E-05
GO:0009605	BP	response to external stimulus	33	17	50	2917	1.25E-07	5.12E-05
GO:0009410	BP	response to xenobiotic stimulus	11	9	20	625	1.92E-07	7.15E-05
GO:0044281	BP	small molecule metabolic process	22	16	38	1934	2.30E-07	7.71E-05
GO:004343	BP	oxoacid metabolic process	16	11	27	1095	2.44E-	7.71E-05

6						07	
GO:0035634	BP	response to stilbenoid	4	1	5	18	2.64E-07	7.73E-05
GO:0006082	BP	organic acid metabolic process	16	11	27	1115	3.49E-07	9.55E-05
GO:0046942	BP	carboxylic acid transport	10	5	15	385	6.32E-07	0.0001619
GO:0006629	BP	lipid metabolic process	18	13	31	1458	7.19E-07	0.0001639
GO:0015849	BP	organic acid transport	10	5	15	389	7.19E-07	0.0001639
GO:0044255	BP	cellular lipid metabolic process	15	11	26	1090	7.87E-07	0.0001699
GO:0045923	BP	positive regulation of fatty acid metabolic process	4	2	6	48	2.45E-06	0.0005027
GO:0006820	BP	anion transport	12	7	19	682	3.09E-06	0.0005611
GO:0048518	BP	positive regulation of biological process	48	33	81	6453	3.11E-06	0.0005611
GO:0062013	BP	positive regulation of small molecule metabolic process	7	3	10	188	3.34E-06	0.0005611
GO:1901568	BP	fatty acid derivative metabolic process	5	5	10	188	3.34E-06	0.0005611
GO:0009719	BP	response to endogenous stimulus	21	12	33	1734	3.42E-06	0.0005611
GO:0045834	BP	positive regulation of lipid metabolic process	7	3	10	190	3.67E-06	0.0005626
GO:0062012	BP	regulation of small molecule metabolic process	10	4	14	388	3.70E-06	0.0005626

GO:0048731	BP	system development	42	25	67	4996	4.62E-06	0.0006759
GO:0010876	BP	lipid localization	11	5	16	513	4.78E-06	0.0006759
GO:0035902	BP	response to immobilization stress	3	2	5	32	5.66E-06	0.0007416
GO:0032502	BP	developmental process	53	29	82	6664	5.93E-06	0.0007416
GO:0007275	BP	multicellular organism development	44	26	70	5349	5.94E-06	0.0007416
GO:0007399	BP	nervous system development	27	14	41	2477	5.97E-06	0.0007416
GO:0050766	BP	positive regulation of phagocytosis	6	1	7	87	7.04E-06	0.0008491
GO:0010942	BP	positive regulation of cell death	12	7	19	725	7.42E-06	0.0008696
GO:0005215	MF	transporter activity	15	10	25	1186	7.96E-06	0.0032783
GO:0045597	BP	positive regulation of cell differentiation	20	5	25	1162	8.11E-06	0.0009242
GO:0019216	BP	regulation of lipid metabolic process	10	4	14	418	8.66E-06	0.000959
GO:0015711	BP	organic anion transport	11	5	16	539	8.89E-06	0.000959
GO:0010243	BP	response to organonitrogen compound	15	9	24	1116	1.27E-05	0.0013307
GO:0046943	MF	carboxylic acid transmembrane transporter activity	6	3	9	179	1.39E-05	0.0032783
GO:000534	MF	organic acid transmembrane transporter activity	6	3	9	180	1.45E-	0.0032783

2							05	
GO:0051239	BP	regulation of multicellular organismal process	34	16	50	3424	1.46E-05	0.0014828
GO:0009991	BP	response to extracellular stimulus	10	7	17	627	1.50E-05	0.0014828
GO:0048856	BP	anatomical structure development	46	29	75	6033	1.52E-05	0.0014828
GO:0050896	BP	response to stimulus	62	46	108	9920	1.60E-05	0.0015234
GO:0051094	BP	positive regulation of developmental process	23	7	30	1619	1.73E-05	0.0016109
GO:0051046	BP	regulation of secretion	9	10	19	774	1.85E-05	0.0016879
GO:0044283	BP	small molecule biosynthetic process	9	9	18	711	2.08E-05	0.0018508
GO:0005576	CC	extracellular region	26	15	41	2598	2.21E-05	0.0088729
