# Supplementary material for: Chemical Composition Analysis of Highland Barley (Hordeum vulgare L.) with Different Modification Methods and Lipid Metabolism Mechanism Analysis of Highland Barley with Microwave Fluidization Modification
Source: Foods. 2026 Apr 17;15(8):1396. doi: 10.3390/foods15081396 (PMC13114515; doi:10.3390/foods15081396)
Supplement: Supplementary file 1 [file foods-15-01396-s001.zip › Table S13.pdf]

Table S13 The top 50 significant KEGG pathways between HFCD and HFCD+HB-1.

PathwayID	Pathway	level1	level2	Up	Down	DEG	Total	Pvalue	adjustPvalue
mmu05216	Thyroid cancer	Human Diseases	Cancer: specific types	1	3	4	37	0.0006213	0.1298542
mmu00140	Steroid hormone biosynthesis	Metabolism	Lipid metabolism	3	2	5	91	0.0028483	0.2615437
mmu00830	Retinol metabolism	Metabolism	Metabolism of cofactors and vitamins	3	2	5	97	0.0037542	0.2615437
mmu05171	Coronavirus disease - COVID-19	Human Diseases	Infectious disease: viral	2	5	7	235	0.0128262	0.5249984
mmu00590	Arachidonic acid metabolism	Metabolism	Lipid metabolism	2	2	4	86	0.0134284	0.5249984
mmu03320	PPAR signaling pathway	Organismal Systems	Endocrine system	4	0	4	89	0.0150717	0.5249984
mmu00770	Pantothenate and CoA biosynthesis	Metabolism	Metabolism of cofactors and vitamins	1	1	2	21	0.0208821	0.5983164
mmu05213	Endometrial cancer	Human Diseases	Cancer: specific types	0	3	3	58	0.0240206	0.5983164
mmu04390	Hippo signaling pathway	Environmental Information Processing	Signal transduction	3	2	5	156	0.0257648	0.5983164
mmu04066	HIF-1 signaling pathway	Environmental Information Processing	Signal transduction	3	1	4	113	0.0327973	0.6779714
mmu04924	Renin secretion	Organismal Systems	Endocrine system	3	0	3	75	0.0462148	0.6779714
mmu0052	Neomycin, kanamycin and	Metabolism	Biosynthesis of other	1	0	1	5	0.052433	0.6779714

4	gentamicin biosynthesis		secondary metabolites					7	
mmu04614	Renin-angiotensin system	Organismal Systems	Endocrine system	2	0	2	36	0.0565178	0.6779714
mmu04012	ErbB signaling pathway	Environmental Information Processing	Signal transduction	2	1	3	84	0.060944	0.6779714
mmu05204	Chemical carcinogenesis - DNA adducts	Human Diseases	Cancer: overview	0	3	3	84	0.060944	0.6779714
mmu04512	ECM-receptor interaction	Environmental Information Processing	Signaling molecules and interaction	2	1	3	88	0.068114	0.6779714
mmu05210	Colorectal cancer	Human Diseases	Cancer: specific types	0	3	3	88	0.068114	0.6779714
mmu05219	Bladder cancer	Human Diseases	Cancer: specific types	0	2	2	41	0.0711023	0.6779714
mmu04610	Complement and coagulation cascades	Organismal Systems	Immune system	2	1	3	91	0.0737329	0.6779714
mmu05202	Transcriptional misregulation in cancer	Human Diseases	Cancer: overview	3	2	5	212	0.0768284	0.6779714
mmu04975	Fat digestion and absorption	Organismal Systems	Digestive system	2	0	2	43	0.0772441	0.6779714
mmu05222	Small cell lung cancer	Human Diseases	Cancer: specific types	1	2	3	93	0.0775907	0.6779714
mmu04921	Oxytocin signaling pathway	Organismal Systems	Endocrine system	0	4	4	152	0.0799515	0.6779714
mmu05231	Choline metabolism in cancer	Human Diseases	Cancer: overview	1	2	3	98	0.0876139	0.6779714
mmu04930	Type II diabetes mellitus	Human Diseases	Endocrine and metabolic disease	1	1	2	48	0.0932831	0.6779714

mmu04979	Cholesterol metabolism	Organismal Systems	Digestive system	2	0	2	49	0.0965989	0.6779714
mmu04514	Cell adhesion molecules	Environmental Information Processing	Signaling molecules and interaction	0	4	4	163	0.0972552	0.6779714
mmu05142	Chagas disease	Human Diseases	Infectious disease: parasitic	2	1	3	103	0.0981555	0.6779714
mmu00591	Linoleic acid metabolism	Metabolism	Lipid metabolism	0	2	2	50	0.0999481	0.6779714
mmu00920	Sulfur metabolism	Metabolism	Energy metabolism	0	1	1	11	0.1117749	0.6779714
mmu04923	Regulation of lipolysis in adipocytes	Organismal Systems	Endocrine system	1	1	2	56	0.120686	0.6779714
mmu05200	Pathways in cancer	Human Diseases	Cancer: overview	3	6	9	542	0.1252168	0.6779714
mmu00430	Taurine and hypotaurine metabolism	Metabolism	Metabolism of other amino acids	0	1	1	13	0.1307265	0.6779714
mmu00561	Glycerolipid metabolism	Metabolism	Lipid metabolism	2	0	2	62	0.1423709	0.6779714
mmu04152	AMPK signaling pathway	Environmental Information Processing	Signal transduction	2	1	3	123	0.1449046	0.6779714
mmu05217	Basal cell carcinoma	Human Diseases	Cancer: specific types	0	2	2	63	0.146063	0.6779714
mmu04380	Osteoclast differentiation	Organismal Systems	Development and regeneration	2	1	3	124	0.1474118	0.6779714
mmu04110	Cell cycle	Cellular Processes	Cell growth and death	2	1	3	125	0.1499333	0.6779714
mmu0001	Glycolysis /	Metabolism	Carbohydrate	2	0	2	67	0.161022	0.6779714

0	Gluconeogenesis		metabolism					6	
mmu0301<br>0	Ribosome	Genetic Information Processing	Translation	0	3	3	130	0.162746<br>7	0.6779714
mmu0503<br>1	Amphetamine addiction	Human Diseases	Substance dependence	0	2	2	68	0.164806<br>2	0.6779714
mmu0472<br>6	Serotonergic synapse	Organismal Systems	Nervous system	1	2	3	131	0.165348<br>7	0.6779714
mmu0516<br>5	Human papillomavirus infection	Human Diseases	Infectious disease: viral	2	4	6	352	0.175262<br>4	0.6779714
mmu0452<br>0	Adherens junction	Cellular Processes	Cellular community - eukaryotes	0	2	2	71	0.176249<br>6	0.6779714
mmu0492<br>0	Adipocytokine signaling pathway	Organismal Systems	Endocrine system	1	1	2	71	0.176249<br>6	0.6779714
mmu0411<br>5	p53 signaling pathway	Cellular Processes	Cell growth and death	1	1	2	72	0.180092<br>3	0.6779714
mmu0521<br>8	Melanoma	Human Diseases	Cancer: specific types	0	2	2	72	0.180092<br>3	0.6779714
mmu0067<br>0	One carbon pool by folate	Metabolism	Metabolism of cofactors and vitamins	0	1	1	19	0.185214	0.6779714
mmu0520<br>7	Chemical carcinogenesis - receptor activation	Human Diseases	Cancer: overview	2	2	4	209	0.185561<br>4	0.6779714
mmu0491<br>7	Prolactin signaling pathway	Organismal Systems	Endocrine system	1	1	2	74	0.187816	0.6779714
