# Supplementary material for: Chemical Composition Analysis of Highland Barley (Hordeum vulgare L.) with Different Modification Methods and Lipid Metabolism Mechanism Analysis of Highland Barley with Microwave Fluidization Modification
Source: Foods. 2026 Apr 17;15(8):1396. doi: 10.3390/foods15081396 (PMC13114515; doi:10.3390/foods15081396)
Supplement: Supplementary file 1 [file foods-15-01396-s001.zip › Table S2.pdf]

Table S2 The selected genes and sequences of primers.

Target gene	Upstream primer (5'-3')	Downstream primer (3'-5')
Cyp2b9	ATCACAGCCAACATCATC	TTATACATCAGATTCAGCAGAT
Cyp2b13	TTGCTGAATCTGATGTAT	GGTAATGAAGTTGAGGAT
Cyp2c38	CAACCAATCCTTAACCAAT	TTCCTTCACTGCTTCATA
Pparg	CAATGGTTGCTGATTACA	CAGAATAATAAGGTGGAGATG
Fabp4	AAACACCGAGATTTCCTT	TTATGATGCTCTTCACCTT
Cd36	AGAATCCAGATAACCATT	GATGTAAGCCTTCAATAG
Plin4	GTATTCATAAGAACACAGACA	CATTGGTGGCTACATTAC
TLR4	GCCCTACCAAGTCTCAGCTA	CTGCAGCTCTTCTAGACCCA
IKKβ	TCAGTCACCCTGGTCAGCTA	CACTTTCTAGCCGGGAGCAG
p-P65	CGAAATCCAACGCAGGGGTCA	GATGCCAAGGCGATGGGTTC
GAPDH	AGTGGCAAAGTGGAGATT	GTGGAGTCATACTGGAACA
