# Supplementary material for: Chemical Composition Analysis of Highland Barley (Hordeum vulgare L.) with Different Modification Methods and Lipid Metabolism Mechanism Analysis of Highland Barley with Microwave Fluidization Modification
Source: Foods. 2026 Apr 17;15(8):1396. doi: 10.3390/foods15081396 (PMC13114515; doi:10.3390/foods15081396)
Supplement: Supplementary file 1 [file foods-15-01396-s001.zip › Table S3.pdf]

**Table S3** The top 30 differential metabolites analysis between HB and HB-1.

| Name | foldChange | log2FoldChange | pvalue | FDR | HB-A | HB-B | HB-C | HB-1-A | HB-1-B | HB-1-C | vip |
|---|---|---|---|---|---|---|---|---|---|---|---|
| Bindarit | 111.35568 | 6.7990314 | 0.003657 | 0.0434391 | 131088.1 | 109062.32 | 108463.01 | 14633142 | 17893943 | 6293001.5 | 1.4641896 |
| N,N-Dimethyl-p-phenylenediamine | 102.87184 | 6.6847042 | 0.0005262 | 0.0167013 | 53182.081 | 43987.446 | 67485.06 | 5794006.1 | 5419321.4 | 5724992.2 | 1.4977886 |
| 6'-Malonylastragalin | 99.442426 | 6.6357896 | 0.002659 | 0.0355274 | 20940.161 | 10006.745 | 25916.975 | 1906694.8 | 1670992.5 | 2076995.1 | 1.4888005 |
| Annuolide C | 76.486295 | 6.2571294 | 0.000329 | 0.0126044 | 201729.36 | 139511.44 | 91667.405 | 9308491 | 14073648 | 9729404.7 | 1.489809 |
| Dopamine | 68.228638 | 6.0923055 | 0.0005598 | 0.0166801 | 16192.244 | 8968.4483 | 11276.753 | 905651.29 | 843854.42 | 736571.54 | 1.4735783 |
| beta-Sitosterol 3-O-beta-D-galactopyranoside | 63.057422 | 5.9785943 | 9.546E-05 | 0.0080628 | 250578.05 | 193485.37 | 361636.75 | 16298262 | 22724827 | 11782286 | 1.4686515 |
| Homophenylalanine | 44.73316 | 5.4832728 | 7.098E-05 | 0.0060386 | 571147.31 | 489431.48 | 463362.85 | 23590052 | 22614701 | 21965973 | 1.4986961 |
| N-Methyltyramine | 26.976044 | 4.7536069 | 0.0013324 | 0.0266687 | 108537.09 | 89262.272 | 68477.994 | 2301962.2 | 2439596.9 | 2441550.8 | 1.4733899 |
| Axid Ar | 25.079923 | 4.648461 | 0.0001966 | 0.0100044 | 100558.89 | 79273.215 | 107154.89 | 2350196.5 | 2284256.8 | 2563158.3 | 1.497407 |
| Dehydrochlortetracycline | 22.798568 | 4.5108713 | 5.119E-06 | 0.0023018 | 301306.42 | 356460.85 | 362169.15 | 7448321.7 | 7400157.9 | 8404610.3 | 1.4979171 |
| (-)-dehydrocostus lactone | 22.042741 | 4.4622318 | 0.0029245 | 0.0375085 | 247502.07 | 198292.59 | 129788.39 | 4126268.4 | 4505028.3 | 4056131.6 | 1.488461 |
| Mesalazine | 22.036497 | 4.461823 | 1.443E-05 | 0.0055665 | 180413.24 | 149146.96 | 148128.46 | 3319657.1 | 3427861.2 | 3779066.2 | 1.4770731 |
| 3-hydroxybenzyl alcohol | 21.6278 | 4.4348157 | 0.0001 | 0.0082 | 615180 | 571028 | 550220 | 117000 | 112545 | 146006 | 1.4971 |

| | | | | | | | | | | | |
|---|---|---|---|---|---|---|---|---|---|---|---|
| | 1 | | 348 | 49 | .23 | .33 | .39 | 17 | 25 | 14 | 824 |
| 5-Acetyl-2,3-dihydro-1,4-thiazine | 17.697405 | 4.1454659 | 4.64E-06 | 0.0022091 | 1402125.7 | 1271123.8 | 1389989.8 | 24310422 | 23217205 | 24381164 | 1.4992623 |
| (Z)-[(4-hydroxyphenyl)acetaldehyde oxime] | 16.594174 | 4.0526049 | 0.0004981 | 0.0163874 | 927465.32 | 793733.12 | 679685.83 | 12738443 | 13255099 | 13847148 | 1.4965417 |
| Ectoine | 14.746705 | 3.8823208 | 0.0002204 | 0.0103959 | 1516448.3 | 1277244.6 | 1234199.8 | 19209337 | 19711975 | 20476836 | 1.4981119 |
| PC(15_0_20_1(11Z)) | 14.382965 | 3.8462892 | 0.0036865 | 0.0430517 | 975800.73 | 891709.72 | 1582673.4 | 17340919 | 15817449 | 16465505 | 1.486985 |
| 4-Aminobenzoic acid | 10.390561 | 3.3772016 | 0.0002883 | 0.0115818 | 116593.9 | 103391.81 | 102894.14 | 936747.21 | 1281057.9 | 1137097.7 | 1.4746597 |
| L,L-Cyclo(leucylprolyl) | 9.8631344 | 3.3020462 | 0.0232616 | 0.1259274 | 803914.51 | 3649147.9 | 1525523.7 | 16888625 | 17242592 | 24836382 | 1.4034195 |
| 3-amino-3-(4-hydroxyphenyl)propanoic acid | 9.6620168 | 3.2723244 | 0.0017395 | 0.0296081 | 192757.98 | 145586.58 | 117058.62 | 1325206.4 | 1551940.7 | 1522966.1 | 1.4658575 |
| Imidazole-4-acetaldehyde | 9.1201687 | 3.1890605 | 3.311E-05 | 0.0042606 | 673934.12 | 517463.98 | 560047.11 | 4737373.1 | 6030448.9 | 5205653.8 | 1.4945857 |
| 3-Methoxytyrosine | 8.7190043 | 3.1241634 | 0.0010409 | 0.0231736 | 1681992.7 | 1480624.5 | 1185620.8 | 12254959 | 12228793 | 13428553 | 1.4709052 |
| Ethyl 2-(methyldithio)propionate | 8.3257281 | 3.0575765 | 4.038E-05 | 0.004669 | 1076498.7 | 917263.95 | 905033.45 | 8090321.4 | 6996982.7 | 9047284.5 | 1.493464 |
| L-Ornithine | 8.1740173 | 3.0310453 | 5.712E-06 | 0.0024331 | 527209.05 | 480517.64 | 447288.6 | 3728642.7 | 3866212.7 | 4298464.8 | 1.4971332 |
| PG 34_1 | 7.7826172 | 2.9602554 | 0.0236987 | 0.1183812 | 29456359 | 25711540 | 26922584 | 126496168 | 156570308 | 355812334 | 1.4078988 |
| ecdysone palmitate | 7.5037044 | 2.907603 | 0.0466821 | 0.171267 | 10941510 | 8078794 | 16295686 | 41836947 | 43938617 | 179225183 | 1.2874242 |
| Epinephrine | 6.75633 | 2.7562419 | 0.0086 | 0.0695 | 225900 | 190570 | 190606 | 127128 | 922561 | 190777 | 1.4593 |

| | | | | | | | | | | | |
|---|---|---|---|---|---|---|---|---|---|---|---|
| | 97 | | 766 | 936 | 1.8 | 1.3 | 1.6 | 20 | 3.6 | 15 | 047 |
| Kaempferol | 6.7550837 | 2.7559736 | 0.0001609 | 0.0096982 | 1583200.4 | 1259960.8 | 1166805.9 | 7956260.4 | 9649324 | 9482079.1 | 1.469892 |
| Carbapenem-3-carboxylic acid | 6.7404221 | 2.7528389 | 0.0001215 | 0.007838 | 902454.31 | 818479.24 | 710010.49 | 5266558.1 | 5230018.8 | 5889012 | 1.4943852 |
| Histamine | 6.5371475 | 2.7086613 | 0.0010577 | 0.0230717 | 665323.34 | 506862.59 | 538862.9 | 3799576.2 | 3816083.8 | 3569718.7 | 1.4941415 |
| Styrene | 6.516423 | 2.7040803 | 0.0371596 | 0.1657823 | 497578.28 | 442192.59 | 1777590.2 | 4986963.6 | 5619266.9 | 7101243.7 | 1.375606 |
| (1S,2R)-Naphthalene 1,2-oxide | 6.166029 | 2.6243417 | 0.0029204 | 0.0375085 | 1091818.6 | 765140.28 | 828227.85 | 5590876.3 | 5346073.4 | 5619989.6 | 1.4896844 |
| Spiroxamine | 6.157539 | 2.6223539 | 7.705E-05 | 0.0060386 | 9456433.2 | 7855379.2 | 8048887.1 | 45121256 | 52230960 | 58807281 | 1.4926219 |
| Iduronate 2-sulfate | 5.9786983 | 2.5798314 | 0.0053255 | 0.0529581 | 736563.85 | 564585.52 | 461805.2 | 3440654.3 | 3535293.4 | 3564225.8 | 1.4618353 |
| DG(18_3(6Z,9Z,12Z)_18_2(9Z,12Z)_0_0) | 5.9608828 | 2.575526 | 0.0398274 | 0.1726678 | 25114730 | 9767056.2 | 5351863.5 | 124628651 | 31166607 | 84032812 | 1.243197 |
| Phenol sulphate | 0.0044234 | -7.82063 | 1.975E-06 | 0.0055665 | 177493298 | 150734799 | 129396007 | 623427.14 | 620476.16 | 780349.3 | 1.4771786 |
| Paromomycin | 0.0117004 | -6.417294 | 0.007152 | 0.0627779 | 9879032 | 6327527.2 | 7235503.7 | 161277.51 | 30743.43 | 82261.457 | 1.4694087 |
| Palatinose | 0.0299118 | -5.063143 | 0.0322749 | 0.1526596 | 7110779.5 | 44286130 | 41094838 | 955762.08 | 925159.25 | 885670.59 | 1.4086563 |
| (卤)-2-Hydroxy-2-phenylacetonitrile | 0.0377802 | -4.726227 | 0.0030461 | 0.0382598 | 15852796 | 25766751 | 11346614 | 576840.97 | 698516.67 | 725712.6 | 1.4828941 |
| 8-Hydroxypinoresinol 8-glucoside | 0.0470156 | -4.410715 | 0.0226802 | 0.1158695 | 2023294.2 | 663385.22 | 362723.61 | 90949.651 | 43223.981 | 9196.0256 | 1.3093489 |
| Limonoate D-ring-lactone | 0.0501358 | -4.318016 | 0.0010919 | 0.0240143 | 11809284 | 19234611 | 18393028 | 905026.9 | 750762.83 | 822768.98 | 1.4706668 |

| | | | | | | | | | | | |
|---|---|---|---|---|---|---|---|---|---|---|---|
| FA 20_3;O4 | 0.0532388 | -4.231377 | 4.817E-05 | 0.0050833 | 4396347.9 | 3623809.8 | 3525488.4 | 196246.57 | 199189.39 | 219240.7 | 1.497278 |
| Magnoline | 0.054332 | -4.202055 | 0.009136 | 0.0697262 | 181818118 | 71360301 | 140324644 | 6832167.8 | 7387320.6 | 7160302 | 1.4479105 |
| Andrographolide | 0.0564669 | -4.146451 | 0.0030051 | 0.0381196 | 2463772.3 | 3082831.6 | 2197536 | 223434.6 | 98927.711 | 114925.26 | 1.4785906 |
| Indole-3-acetonitrile-glycylcysteine conjugate | 0.0665923 | -3.9085 | 0.0215016 | 0.1116437 | 89656561 | 295220308 | 286119707 | 13950333 | 15891571 | 14841332 | 1.414694 |
| Oxidized glutathione | 0.070991 | -3.81622 | 0.0106104 | 0.0739342 | 926406.72 | 942385.56 | 974130.07 | 64495.75 | 99984.356 | 37341.775 | 1.4459062 |
| 17-Hydroxymethylethisterone | 0.0730198 | -3.775569 | 0.0005918 | 0.0175671 | 1193038.8 | 1092833.3 | 1341627.6 | 67112.867 | 93082.927 | 104683.5 | 1.4887237 |
| Clorobiocin | 0.0766121 | -3.706284 | 0.0042716 | 0.0475121 | 14551098 | 9157731.6 | 5013982.4 | 481178.56 | 856582.39 | 862753.55 | 1.4188776 |
| D-myo-Inositol 4-phosphate | 0.0800842 | -3.642339 | 0.0216724 | 0.1202 | 1693472 | 1357560.9 | 1481109.7 | 45363.974 | 169474.57 | 148114.4 | 1.4237297 |
| Dopaquinone | 0.0854146 | -3.549373 | 1.56E-05 | 0.0055665 | 9873800.8 | 8455875.7 | 7835463.7 | 720842.54 | 691511.03 | 822531.74 | 1.4750745 |
| Leucyl-Asparagine | 0.088982 | -3.490342 | 0.0115017 | 0.0818671 | 10687417 | 7109514.2 | 20543130 | 1307198.8 | 944574.32 | 1159803.3 | 1.4443521 |
| gamma-Glutamylcysteine | 0.0952806 | -3.391674 | 0.0205733 | 0.1174547 | 1915894.5 | 2487064.1 | 714919.68 | 154760.84 | 134960.53 | 197912.9 | 1.4166229 |
| Dioscin | 0.099447 | -3.329929 | 0.0365442 | 0.1497817 | 2332731.1 | 763311.46 | 957535.61 | 29698.318 | 154532.96 | 218884.86 | 1.2772627 |
| 2-Carboxyarabinitol 1-phosphate | 0.1000863 | -3.320683 | 5.261E-05 | 0.0067851 | 725113.75 | 593628.89 | 577511.99 | 64181.05 | 53180.422 | 72427.663 | 1.4717753 |
| Fructosyl-lysine | 0.1007691 | -3.310874 | 3.905E-05 | 0.0045805 | 984307.12 | 952696.72 | 809363.32 | 98165.396 | 91426.619 | 87157.043 | 1.4974607 |
| Deptropine | 0.10638 | -3.232701 | 0.0163 | 0.1025 | 225243 | 185774 | 156716 | 291532 | 893507 | 223072 | 1.4339 |

| | | | | | | | | | | | |
|---|---|---|---|---|---|---|---|---|---|---|---|
| | | | 74 | 655 | 02 | 32 | 78 | 7.9 | .47 | 2.5 | 804 |
| 9,10-Epoxy-18-hydroxy-octadecanoic acid | 0.107795 | -3.213637 | 0.0116709 | 0.0828532 | 2619558.9 | 1685178.9 | 727535.84 | 97461.208 | 166147.35 | 278845.56 | 1.3700257 |
| Thymol methyl ether | 0.1096108 | -3.189538 | 1.638E-05 | 0.0033142 | 24173238 | 20223044 | 20148365 | 2509590.2 | 2403762.8 | 2161436.3 | 1.4962277 |
| Cammaconine | 0.1100089 | -3.184308 | 0.0001779 | 0.010041 | 4628672.4 | 4669440.4 | 4448119.1 | 554863.47 | 477806.45 | 479538.06 | 1.4778596 |
| L-Glutamine | 0.1127132 | -3.149271 | 7.691E-05 | 0.0060386 | 217930728 | 184014156 | 179763528 | 22670224 | 22333124 | 20562880 | 1.4966468 |
| Pyroglutamic acid | 0.1181987 | -3.080714 | 0.0001984 | 0.0100044 | 199374313 | 176086601 | 179185660 | 22186574 | 21770929 | 21600992 | 1.4984946 |
| Margaroylglycine | 0.1196443 | -3.063177 | 0.0021804 | 0.0319667 | 126144865 | 103301826 | 84474388 | 12657502 | 11964485 | 12936873 | 1.4906518 |
| Neoabietol | 0.1255272 | -2.993928 | 1.503E-05 | 0.0032017 | 3704167.8 | 3022005.5 | 3261628.3 | 389626.53 | 404742.66 | 459371.9 | 1.4947682 |
| Isowertin 2'-rhamnoside | 0.1344161 | -2.895222 | 0.0267995 | 0.136331 | 121527245 | 54229469 | 39544347 | 10582223 | 9707761.9 | 8649953.2 | 1.4115146 |
| Urobilinogen | 0.1462032 | -2.773954 | 0.013562 | 0.0851259 | 164122325 | 318744975 | 325558463 | 39956961 | 37924798 | 40312651 | 1.4385064 |
| GDP-beta-L-colitose | 0.1481012 | -2.755345 | 0.0354289 | 0.1619923 | 84122246 | 130830535 | 39799472 | 23671399 | 9977497.7 | 4080215.5 | 1.2910326 |
| Epipregnanolone | 0.1528904 | -2.70943 | 0.000921 | 0.0216709 | 8033923.2 | 5517660.3 | 6823175.7 | 1012832.3 | 967542.7 | 1134730.1 | 1.4868281 |
| Oryzalexin E | 0.1530275 | -2.708137 | 0.0028586 | 0.0374608 | 15906147 | 10753949 | 10485020 | 1914703.4 | 1752549.4 | 2016971.8 | 1.4618267 |
| NCI60_031845 | 0.155443 | -2.685543 | 0.0408413 | 0.1752511 | 2731027.6 | 849058.48 | 2664010.6 | 285896.79 | 311093.01 | 373611.2 | 1.3619013 |
| 9S-HpOTrE | 0.1557944 | -2.682285 | 0.0011822 | 0.0251967 | 60056746 | 49976205 | 44613618 | 7713871.2 | 8012251.5 | 8366946.8 | 1.4715773 |

vip: OPLS-DA first principal component variable importance value projection, vip≥ 1.

foldChange: Ploidy change between two groups, foldChange ≥ 1.

log2 (foldChange): log2 value of ploidy change.

*p*-value: Statistically significant difference, *p*-value ≤ 0.05.

FDR: *P* value Correction value.
