# Supplementary material for: Chemical Composition Analysis of Highland Barley (Hordeum vulgare L.) with Different Modification Methods and Lipid Metabolism Mechanism Analysis of Highland Barley with Microwave Fluidization Modification
Source: Foods. 2026 Apr 17;15(8):1396. doi: 10.3390/foods15081396 (PMC13114515; doi:10.3390/foods15081396)
Supplement: Supplementary file 1 [file foods-15-01396-s001.zip › Table S4.pdf]

Table S4 The top 30 differential metabolites analysis between HB and HB-2.

Name	foldChange	log2FoldChange	pvalue	FDR	HB-A	HB-B	HB-C	HB-2-A	HB-2-B	HB-2-C	vip
N-[5-Methyl-8-(4-methylpiperazin-1-yl)-1,2,3,4-tetrahydronaphthalen-2-yl]-4-morpholin-4-ylbenzamide	198.58802	7.6336348	0.0003845	0.0055391	110736.1	73468.847	116867.83	31034411	16328425	12426610	1.2674478
Lauroyl diethanolamide	161.49341	7.3353315	0.005354	0.0227452	10839567	3048756.5	2747808.9	743853389	968677493	974094930	1.2599371
PA(16_0_16_0)	88.020571	6.4597688	0.0006356	0.0070882	14049174	7539789.9	6342106.1	1.027E+09	1.045E+09	385795415	1.2548964
N-(gamma-Glutamyl)ethanolamine	87.772216	6.4556924	9.495E-05	0.0025111	68432.376	39221.849	48297.853	6399247.1	4095971.4	3193041	1.2698773
Pheophytin a	76.904606	6.2649981	0.0056642	0.0236292	29306925	11888331	5316443.8	1.26E+09	795603390	1.522E+09	1.2443134
ST 28_2;O4	66.909399	6.064137	0.0200853	0.0554931	26049016	2659907.6	10741001	807549944	926984124	905036633	1.2240077
Chlortetracycline	49.130751	5.6185544	0.0028845	0.0157468	438025.08	388669.84	273044.02	30755491	12983468	10292042	1.2539927
Venoterpine	46.293286	5.5327311	0.0001451	0.0030826	854169.99	632038.39	645961.43	35283718	31242442	32178987	1.2765181
8-Hydroxypinoresinol 8-glucoside	34.563341	5.1111708	0.0143389	0.0441288	2023294.2	663385.22	362723.61	42587141	31960287	30850132	1.2306424
ecdysone palmitate	33.309954	5.0578815	0.0021144	0.0133292	10941510	8078794	16295686	465189125	182705117	528479761	1.2441256
Sventenic acid	29.601786	4.8876123	0.0059961	0.0243484	607626.17	2109597	1055703.5	46187191	34334395	31163783	1.2492424
15-HETE	27.8705	4.8006717	0.0005	0.0060	198175	138000	178248	405858	354041	673830	1.2670

	91		587	287	01	81	39	405	859	403	518
PE(16_0_16_0)	27.59345	4.7862539	3.959E-05	0.0023354	156620764	124366699	115493911	4.039E+09	4.06E+09	2.841E+09	1.270525
12(R)-HPETE	26.809385	4.7446662	0.0027025	0.0152127	102036769	152561223	190018259	6.178E+09	3.731E+09	2.011E+09	1.2444072
Genistein	26.088962	4.7053676	0.0019966	0.0122877	901573.53	871016.31	747124.72	30726470	19176266	15834002	1.2680919
Oleanolic aldehyde	24.748131	4.6292477	0.0003445	0.004766	1458518.7	2625719.7	2723168.9	72117817	53317097	43035691	1.2633883
Nitrofen	23.265873	4.5401434	9.879E-06	0.0011765	1362428.9	1037591.5	1177189.4	30944890	24954244	27327774	1.2759145
ST 21_4;O2	22.382289	4.4842857	0.0029168	0.0158797	6465814	3483342.8	2627239.4	113666887	87483190	80338458	1.2552471
Hydroxychlorobactene	20.783819	4.3773889	0.0377114	0.0847067	1234481	1669130.8	828453.65	9325554.7	58783775	9457245.8	1.150489
Mannose 6-phosphate	18.730094	4.2272863	0.0001574	0.0031604	8261211.3	8114475.3	9121725.4	187591281	146188206	143789443	1.2759962
Axid Ar	17.836642	4.1567721	0.0007306	0.0070223	100558.89	79273.215	107154.89	2271333.8	1615158.2	1232392.2	1.2664942
Behenoylglycine	16.714896	4.0630625	0.0004514	0.0055353	187754.69	153495.08	100342.22	3458143.7	1887798.7	2035221.7	1.2557874
7,8-Dihydroneopterin	16.111259	4.0099974	0.0118363	0.0380074	417272.09	286742.1	1055428.2	11577592	8905424.3	7863816.1	1.2336138
7-Hydroxyflavone	16.024224	4.0021825	0.0002173	0.0039171	838410.95	996573.44	1108211.9	17744422	11838336	17579678	1.2706262
N-Acetyl-O-demethylpuromycin	15.091502	3.9156645	0.0048661	0.0213754	1300707.4	2431190.7	840813.37	26417042	25501441	17090600	1.2414108
PG 34_1	14.850123	3.892403	0.0343857	0.0803216	29456359	25711540	26922584	572617750	518366022	128070027	1.1915884

3,4-dihydroxyphenylpyruvate	13.4185	3.7461515	0.0002696	0.0047155	4276763	4032254.8	3898314.7	61439620	55298078	47066391	1.2732925
Gallic acid	13.266789	3.7297473	0.0002003	0.0043757	1238015.3	945300.49	800771.48	17219760	11521205	10848291	1.2620045
GDP-6-deoxy-D-altrose	12.700352	3.6667966	5.59E-06	0.0010054	3019988.9	2507787.9	2625936.1	30800588	35890965	36863469	1.2758971
O-Geranylvanillin	12.252559	3.6150112	0.003304	0.0165467	306083.41	159461.43	257388.23	3305769.8	2739597.7	2812413	1.2626736
Deoxylimonate	11.951657	3.5791387	0.043202	0.0943935	8134329.2	1077698.2	8086205	86490197	42443131	77809209	1.1352784
bisacodyl	11.938143	3.5775066	0.0001478	0.0030826	4547882.6	6378320.2	5704715.2	83603073	55738618	59200593	1.2678385
4-Gallocatechol	11.784352	3.5588005	0.0001591	0.0031686	8064790.6	5307301.1	7594406.7	96427157	85710844	64938586	1.2660241
Neryl propionate	11.342658	3.5036869	0.0027254	0.0146942	5646902.6	7887153.1	7072905	110918742	69635764	53183204	1.2542808
Homoarecoline	11.108307	3.4735671	0.0006823	0.0067427	1525521.6	1053309.2	1125884.7	14880996	13460593	12811529	1.2718955
N-Nonanoylglycine	0.0008201	-10.25197	0.0012933	0.0095291	91716466	73525356	74493378	108977.64	36865.126	50756.656	1.2730413
n-[(2r)-1-Phenylpropan-2-yl]prop-2-yn-1-amine	0.0012181	-9.681132	9.405E-07	0.0007186	422720522	378733292	376196185	539098.76	444428.21	450988.37	1.2781209
PC(15_0_18_3(9Z,12Z,15Z))	0.0024214	-8.68993	0.0006066	0.0063673	57672259	96417908	24788011	197074.76	150213.85	85850.78	1.2636382
(3beta,5alpha)-3-Hydroxypregn-16-en-20-one	0.0024681	-8.662396	0.0002711	0.004344	55895669	42507976	46973970	153501.63	77801.387	127500.42	1.2750108
Epiandrosterone	0.0029539	-8.403147	0.0075976	0.0285192	39179082	29978343	28138306	25036.194	71857.756	190511.08	1.255163
avenastenone	0.00306	-8.350213	0.0031	0.0163	244504	191983	178272	118072	36215.	34094.	1.2624

	43		118	669	72	18	74	.86	229	749	194
6-Hydroxyoctadecanoic acid	0.003086	-8.340063	0.0065112	0.0257678	21244833	14991662	16387792	14497.584	69863.813	78035.247	1.257377
PC(20_4(8Z,11Z,14Z,17Z)_18_3(9Z,12Z,15Z))	0.0030993	-8.333839	0.0011772	0.0089707	35084743	43738201	27900773	51052.886	126015.19	153701.35	1.2690206
Solanapyrone A	0.0031167	-8.325783	0.0001602	0.0031757	181895127	125707581	94176762	281127.73	671610.84	299471.9	1.2701675
Creatine	0.0035682	-8.130585	1.298E-06	0.0011332	127750675	147746305	112250149	539906.8	400362.45	443292.43	1.2742109
[(1S,2S,4R,5S)-9-Methyl-3-oxa-9-azatricyclo[3.3.1.02,4]nonan-7-yl] (2S)-3-hydroxy-2-phenylpropanoate	0.0045583	-7.777284	2.26E-06	0.0007568	57796149	47612072	45830645	258744.6	191051.23	239598.37	1.2772604
PC(P-16_0_18_1(9Z))	0.0045672	-7.774464	7.009E-06	0.0010505	14545025	16480079	15479379	66696.518	64760.975	80939.292	1.27788
PC(15_0_18_4(6Z,9Z,12Z,15Z))	0.0056156	-7.476336	0.001243	0.009297	751498234	1.716E+09	948327775	7218961.2	6222557.9	5740436.4	1.2723442
PE 34_1	0.006429	-7.281181	0.0022588	0.0133336	370992897	153773072	213527364	1484530.6	1580635.9	1681350.7	1.2715068
(+)-O-methylkolavelool	0.0065372	-7.25711	0.0012522	0.0093229	84155904	66590877	77679298	293357	675830.35	524081.36	1.2720153
Milrinone	0.0075725	-7.045014	2.145E-06	0.0007568	83503569	69706369	70266720	605669.55	543760.02	542847.87	1.2779248
Glycocholic acid	0.0093284	-6.744161	0.0096137	0.033221	64006387	48396067	47389284	1058225.5	191173.27	241196.31	1.248674
PC(20_5(5Z,8Z,11Z,14Z,17Z)_P-16_0)	0.0096906	-6.689202	0.0028297	0.0150171	235543310	427275546	705822143	4967882.7	4607088.7	3687949.5	1.2646879
2,5-Dichloro-1,4-benzoquinone	0.00969	-6.688474	0.0001	0.0027	372762	683599	743838	46706.	49185.	78645.	1.2693

	55		149	318	5.2	4	8.2	456	609	843	489
Calystegine C1	0.0099337	-6.653452	4.114E-06	0.0009249	54217617	42504827	42267651	551039.57	406737.81	422909.83	1.2765392
5-hydroxypseudobaptigenin	0.0102459	-6.608804	0.0115633	0.0379403	3793900.1	17602509	16356265	137378.4	137617.75	111815.41	1.2422095
3-Chloro-cis,cis-muconic acid	0.0107151	-6.544212	1.64E-06	0.0007568	202381197	252130397	247754279	2640944.5	2647490.3	2236409.2	1.2773787
Cinchonidine	0.0122322	-6.353196	0.0372192	0.0837172	60147411	78841086	75850089	55979.363	2160095.4	411831.13	1.1863602
PC(P-18_0_16_0)	0.0133556	-6.226413	0.0021062	0.012768	52031607	17098732	40081656	563475.44	539830.57	355284.02	1.259274
Myxol 2-(2,4-di-O-methyl-fucoside)	0.0137491	-6.184515	0.0046481	0.0207487	100056176	155877005	46405258	1139029.7	1519925.9	1497938	1.2601291
PC(O-16_1(9Z)_18_2(9Z,12Z))	0.0141962	-6.138355	2.893E-05	0.0016535	112416783	75671397	110383510	1170364.8	1549118	1517669.9	1.2747585
Urobilinogen	0.0150745	-6.051746	0.0026709	0.0151724	164122325	318744975	325558463	3952109.8	4258144.6	3976356.3	1.2676981
3-O-Acetylepisamarcandin	0.0152796	-6.032248	0.0001907	0.00358	22205820	33858523	45579203	666678.32	466383.43	420012.98	1.2687995
N-Acetylneuraminic acid	0.0155195	-6.009777	0.0007408	0.0075035	8725561.9	7347298.3	6547315.1	155785.05	75991.811	119276.2	1.2679113
berythromycin	0.015707	-5.992448	0.0361927	0.0823355	428170498	76288319	38671546	2660025.1	2816528.2	3054400.1	1.1905377
Usambarine	0.0161426	-5.952984	0.0001603	0.0039393	105642246	85586502	75213877	1337168.6	1478679.6	1485227	1.2741177
PC(14_0_18_0)	0.0161721	-5.95035	0.0037398	0.0179196	128898044	224448182	328904364	3445831.1	3603609	3983980.1	1.2663421
Cholesterol	0.0164657	-5.92439	0.0010167	0.0087194	14403629	13078418	13757792	171474.02	215857.23	291712.89	1.2724023

N-Acetyl-glucosamine 1-phosphate	0.0167877	-5.896456	1.816E-05	0.0015108	129456578	109477474	113297636	2328399.1	1816486.7	1768256.7	1.276829
2-Methylene-4-oxopentanedioic acid	0.018688	-5.74174	0.0091205	0.0321342	846478462	559733157	226098832	10964670	10380405	9159617.1	1.2533647

vip: OPLS-DA first principal component variable importance value projection, vip≥ 1.

foldChange: Ploidy change between two groups, foldChange ≥ 1.

log2 (foldChange): log2 value of ploidy change.

p-value: Statistically significant difference, *p*-value ≤ 0.05.

FDR: *P* value Correction value.
