# Supplementary material for: Chemical Composition Analysis of Highland Barley (Hordeum vulgare L.) with Different Modification Methods and Lipid Metabolism Mechanism Analysis of Highland Barley with Microwave Fluidization Modification
Source: Foods. 2026 Apr 17;15(8):1396. doi: 10.3390/foods15081396 (PMC13114515; doi:10.3390/foods15081396)
Supplement: Supplementary file 1 [file foods-15-01396-s001.zip › Table S5.pdf]

Table S5 The top 30 differential metabolites analysis between HB and HB-3.

Name	foldChange	log2FoldChange	pvalue	FDR	HB-A	HB-B	HB-C	HB-3-A	HB-3-B	HB-3-C	vip
6'-Malonylastragalin	2496.4311	11.285651	0.0011204	0.0096981	20940.161	10006.745	25916.975	49043152	43736670	49176940	1.2621113
Luteolinidin	692.463	9.4355932	0.0061568	0.0259515	7100556.9	5423061.8	5336176.1	1.101E+09	5.278E+09	5.988E+09	1.2471304
Dehydrochlortetracycline	292.41805	8.1918885	6.24E-05	0.0024525	301306.42	356460.85	362169.15	75538386	112833435	109875996	1.2636896
Annuolide C	217.46896	7.7646657	0.0012849	0.010451	201729.36	139511.44	91667.405	33001686	28790264	32352144	1.2615762
Mesalazine	209.5644	7.7112498	2.48E-05	0.0030437	180413.24	149146.96	148128.46	33745273	34493158	31868103	1.2763147
Homophenylalanine	184.14209	7.5246756	1.43E-05	0.0011703	571147.31	489431.48	463362.85	98424820	90226193	91970790	1.2651822
N,N-Dimethyl-p-phenylenediamine	144.69115	7.1768328	0.0002433	0.0045598	53182.081	43987.446	67485.06	8519335.7	7737711.6	7567013.9	1.263559
Axid Ar	118.52032	6.8889906	7.49E-05	0.0026234	100558.89	79273.215	107154.89	11572362	10590895	11850533	1.2644673
3-hydroxybenzyl alcohol	111.76379	6.8043091	1.17E-05	0.0011414	615180.23	571028.33	550220.39	65968254	64112387	63989246	1.2653816
2-Pyrone-4,6-dicarboxylate	109.20269	6.7708646	6.45E-05	0.0045404	1574722.8	1328497.4	1391254.5	159176528	156214766	153576907	1.2764435
Carbapenem-3-carboxylic acid	101.22274	6.6613897	1.09E-05	0.0011414	902454.31	818479.24	710010.49	82144246	76869010	87053565	1.2650138
2-Hydroxybenzalpyruvate	82.954076	6.374241	0.0041594	0.0200968	144186.42	97809.979	51524.014	8557679.7	7945868.4	7845166.9	1.2552433
N-(gamma-	80.2240	6.325963	0.0003	0.0053	68432.3	39221.8	48297.8	420826	369215	461069	1.2621

Glutamyl)ethanolamine	56		39	499	76	49	53	0.1	0.9	7.3	835
Ectoine	71.596303	6.1618132	0.0011459	0.0098658	1516448.3	1277244.6	1234199.8	113720951	60837193	113824089	1.259443
(-)-dehydrocostus lactone	69.791963	6.124989	0.0013686	0.0108441	247502.07	198292.59	129788.39	12951812	12789372	14429886	1.2610772
N-Methyltyramine	65.021016	6.0228342	0.0006711	0.0096217	108537.09	89262.272	68477.994	5966857.7	5879860.7	5466906.2	1.2740295
beta-Sitosterol 3-O-beta-D-galactopyranoside	61.374477	5.9395669	0.0033982	0.0213453	250578.05	193485.37	361636.75	16139681	6894389.3	26415355	1.2485166
7,8-Dihydroneopterin	57.203096	5.8380213	0.0077395	0.0301568	417272.09	286742.1	1055428.2	36175274	33546409	30923868	1.243373
Tryptophol	56.745987	5.8264465	0.0093878	0.0374144	102895.7	89088.702	328473.31	9968510.4	9959735.8	9605640.6	1.2530807
Dopamine	55.548152	5.795667	0.000156	0.0056187	16192.244	8968.4483	11276.753	773383.44	702088.06	548561.23	1.2705269
(Z)-[(4-hydroxyphenyl)acetaldehyde oxime]	46.410359	5.536375	6.47E-06	0.0010569	927465.32	793733.12	679685.83	43118061	32609134	35698707	1.2632667
PC(15_0_20_1(11Z))	36.518409	5.190552	0.0019296	0.0130477	975800.73	891709.72	1582673.4	41150440	40436008	44408774	1.2588305
O-Geranylvanillin	34.7288	5.1180607	0.003669	0.0186884	306083.41	159461.43	257388.23	3557584.3	11945834	9603180.4	1.2303683
3-amino-3-(4-hydroxyphenyl)propanoic acid	31.509705	4.9777244	0.0016408	0.0152282	192757.98	145586.58	117058.62	4873701.8	4779645.2	4696273.1	1.2723206
Tetraprenol	30.942162	4.9515021	2.05E-05	0.0014671	191460.56	233744.61	222105.24	6475022.2	6477111.4	7077050	1.2642664
Kaempferol	30.620683	4.9364346	0.0004459	0.0082095	1583200.4	1259960.8	1166805.9	40368025	42494422	39925486	1.274832

5-Acetyl-2,3-dihydro-1,4-thiazine	30.286752	4.920615	1.72E-07	0.0004671	1402125.7	1271123.8	1389989.8	43195399	38747614	41119311	1.2650738
7,8-dihydromonapterin	26.197982	4.7113838	0.0004057	0.0059047	754248.48	781529.77	430261.15	21461957	14965722	15078586	1.2540017
(1S,2R)-Naphthalene 1,2-oxide	25.760218	4.6870729	0.0004683	0.0063375	1091818.6	765140.28	828227.85	24447635	21913241	22810121	1.2629639
3,4-Dihydroxyhydrocinnamic acid	25.593666	4.6777149	0.0002051	0.0060448	315837.83	290829.32	267517.28	6362546.1	6890813	9120224.8	1.273355
Histamine	25.249521	4.6581841	0.0015988	0.0117949	665323.34	506862.59	538862.9	20949099	11195402	11058663	1.2529889
4-Aminobenzoic acid	24.714954	4.6273123	5.98E-07	0.0007118	116593.9	103391.81	102894.14	2564860.7	2541779.5	2873320.6	1.2759897
PS(14_0_14_0)	24.348277	4.6057478	0.0185677	0.0521223	7113550.3	2271326.8	1604623.1	19873911	89381676	158319805	1.138124
Coniferaldehyde	23.981915	4.583875	0.0003899	0.0077745	833358.55	790639.9	639144.58	18725844	17849945	17698715	1.2749922
Tilmicosin	23.292739	4.5418084	0.0336813	0.0806117	2910643.7	2202664.2	844589.25	61061869	69683091	8030782.7	1.1208126
Phenol sulphate	0.0037113	-8.07386	7.20E-05	0.0045404	177493298	150734799	129396007	569042.28	593620.17	535717.86	1.2760122
2,5-Dichloro-1,4-benzoquinone	0.0052987	-7.560141	0.0072634	0.0289284	3727625.2	6835994	7438388.2	63262.609	24946.223	7178.6952	1.2321262
Methacholine	0.0115839	-6.431736	7.82E-07	0.000575	318305995	276079567	278359150	3253561.5	3275245.4	3580972.4	1.2647376
PC(15_0_18_4(6Z,9Z,12Z,15Z))	0.0135763	-6.202768	0.0256024	0.0643278	751498234	1.716E+09	948327775	21263446	23507896	1602708.1	1.1837854
Imazamox	0.0187061	-5.74035	0.0020123	0.0166364	27524614	12864638	8833877.8	349083.61	358659.78	213027.91	1.2538079

6-Oxoneomycin C	0.0365198	-4.775177	0.0002377	0.0063049	13614299	18021538	14119615	637718.54	633683.98	399578.34	1.2696622
Linuron	0.0534529	-4.225588	0.0104012	0.0396737	103836151	85547250	77092937	3029751.6	8491050.1	2723135.5	1.2409164
Solanapyrone A	0.0575273	-4.11961	0.001022	0.0091805	181895127	125707581	94176762	10890517	7576829.4	4645929.1	1.2364337
Deptropine	0.0611048	-4.032571	0.0036189	0.0185601	22524302	18577432	15671678	1023092.3	704220.13	1741814.5	1.2394672
PC(20_5(5Z,8Z,11Z,14Z,17Z)_16_0)	0.0681226	-3.875723	0.0478777	0.0986754	19732222	101908338	136469397	4135434.2	6466804.4	6980877.7	1.1295276
GDP-beta-L-colitose	0.0710155	-3.815721	0.0049669	0.0227232	84122246	130830535	39799472	10091679	3956092.8	4043599.3	1.1930689
Dopaquinone	0.0777421	-3.68516	0.0002843	0.0069344	9873800.8	8455875.7	7835463.7	678129.05	654370.85	701632.91	1.2747352
PE 34_1	0.0781058	-3.678426	0.0020416	0.0134644	370992897	153773072	213527364	29267343	13024329	15373331	1.2171927
2-Oxazolidinone	0.0783984	-3.673032	0.0004076	0.0059047	5.083E+09	3.5E+09	2.825E+09	380638598	282364912	231343410	1.2453229
Heptadecanoic acid	0.0789015	-3.663804	1.11E-05	0.0011414	1.197E+09	1.031E+09	1.031E+09	96938015	78678510	81507804	1.2625008
Vinblastine	0.0792943	-3.65664	0.0123921	0.0435746	15356636	11421837	5105359.2	1009718.5	762227.08	756258.98	1.2295078
Cinchonidine	0.0794607	-3.653614	0.0258565	0.0647454	60147411	78841086	75850089	10773606	2072233.4	4225387.9	1.1935347
PC(20_5(5Z,8Z,11Z,14Z,17Z)_P-16_0)	0.0871055	-3.521093	0.0048152	0.0221944	235543310	427275546	705822143	32583053	59147191	27485851	1.2056748
3-Chloro-cis,cis-muconic acid	0.0887374	-3.494314	5.02E-05	0.0022019	202381197	252130397	247754279	24634117	19495304	18187802	1.26043
Acetol phosphate	0.09373	-3.415313	0.0120	0.0397	542226	245413	231974	349500	309060	297144	1.2275

	21		226	804	074	032	186	23	67	15	954
Paromomycin	0.0965327	-3.372839	0.0024751	0.0150496	9879032	6327527.2	7235503.7	740167.43	791045.84	731711.94	1.2562164
Phosphocreatinine	0.0978515	-3.353263	0.0313727	0.0739138	41841043	10845914	17757568	2354773	2221608.5	2316718	1.1862009
Rhodomycinone	0.1020351	-3.292863	0.0010311	0.0092413	164403954	94911476	170590613	14843495	9972754.7	19049242	1.2307054
Pinostilbenoside	0.1032046	-3.27642	0.001999	0.0133044	125574129	88207736	70175825	14570766	8036567.1	6698417	1.2265273
2,3,7,8-Tetrachlorodibenzo-p-dioxin	0.104024	-3.265011	0.0155547	0.0468843	8917818.8	7300006.8	7058984.7	1387877.8	553320.32	480149.07	1.2165753
7,8-diaminopelargonate	0.107884	-3.212447	0.0021328	0.0137538	215083816	104358758	166343451	20883447	17930244	13594855	1.234344
Mitraphylline	0.1098309	-3.186644	0.0004386	0.0081572	44279808	30902537	31524605	4325445.4	3901544.2	3492732.1	1.2676166
2-Methylene-4-oxopentanedioic acid	0.1104921	-3.177985	0.0291374	0.0703907	846478462	559733157	226098832	69955068	57341434	53060865	1.1787597
Isowertin 2'-rhamnoside	0.1119581	-3.158969	0.0225342	0.0590767	121527245	54229469	39544347	8829939.1	7302585	7972171.3	1.2001472
PC(15_0_18_3(9Z,12Z,15Z))	0.1206922	-3.050595	0.018471	0.0519409	57672259	96417908	24788011	10131110	8401266.5	3056829.7	1.1237496
Cyclic ADP-欽媟 ibose	0.1211268	-3.04541	0.0206125	0.0558289	23232837	36119584	29170168	6161255.7	2966735.2	1594466.3	1.1904954
N-Methyl-4-dimethylallyltryptophan	0.1248401	-3.001846	5.92E-06	0.0010569	7319142.7	6713844.3	6341688.8	769464.48	889574.51	884537.98	1.262647
3b,9b-Dihydroxy-6b-angeloyloxy-7(11)-eremophilen-12,8b-olide	0.1316507	-2.925212	0.0002367	0.0045144	61742829	46748616	46417063	7117399.1	6093029	7183391.3	1.2571549

(卤)-2-Hydroxy-2-phenylacetonitrile	0.1319329	-2.922124	0.0233154	0.0602555	15852796	25766751	11346614	845174.24	3911469.9	2231332.5	1.1443571
Prostaglandin F1a	0.1320956	-2.920345	0.0275172	0.0705587	9449674.2	5289430	5302239.5	1751826.4	507775.74	387772.09	1.162403

vip: OPLS-DA first principal component variable importance value projection, vip≥ 1.

foldChange: Ploidy change between two groups, foldChange ≥ 1.

log2 (foldChange): log2 value of ploidy change.

p-value: Statistically significant difference, *p*-value ≤ 0.05.

FDR: *P* value Correction value.
