# Supplementary material for: Chemical Composition Analysis of Highland Barley (Hordeum vulgare L.) with Different Modification Methods and Lipid Metabolism Mechanism Analysis of Highland Barley with Microwave Fluidization Modification
Source: Foods. 2026 Apr 17;15(8):1396. doi: 10.3390/foods15081396 (PMC13114515; doi:10.3390/foods15081396)
Supplement: Supplementary file 1 [file foods-15-01396-s001.zip › Table S6.pdf]

Table S6 The top 50 significant KEGG pathways between HB and HB-1.

Pathway	level1	level2	Up	Down	DEM	Total	Pvalue	FDR
Aminoacyl-tRNA biosynthesis	Genetic Information Processing	Translation	0	9	9	52	8.02E-05	0.0050539
D-Amino acid metabolism	Metabolism	Metabolism of other amino acids	1	9	10	69	0.0001528	0.0050539
Biosynthesis of amino acids	Metabolism	Global and overview maps	3	11	14	128	0.0001763	0.0050539
Cutin, suberine and wax biosynthesis	Metabolism	Lipid metabolism	0	6	6	27	0.0003153	0.0067002
ABC transporters	Environmental Information Processing	Membrane transport	1	13	14	138	0.0003895	0.0067002
Arginine biosynthesis	Metabolism	Amino acid metabolism	1	4	5	23	0.0011345	0.0162617
Tyrosine metabolism	Metabolism	Amino acid metabolism	7	2	9	78	0.0017775	0.019732
Metabolic pathways	Metabolism	Global and overview maps	38	91	129	3063	0.0018355	0.019732
Glutathione metabolism	Metabolism	Metabolism of other amino acids	1	5	6	38	0.0021162	0.0202218
Linoleic acid metabolism	Metabolism	Lipid metabolism	1	4	5	28	0.0028639	0.0246292
Steroid biosynthesis	Metabolism	Lipid metabolism	0	7	7	57	0.0040226	0.0314498
alpha-Linolenic acid metabolism	Metabolism	Lipid metabolism	0	6	6	44	0.0045269	0.0324424

2-Oxocarboxylic acid metabolism	Metabolism	Global and overview maps	3	8	11	144	0.0141077	0.093328
Alanine, aspartate and glutamate metabolism	Metabolism	Amino acid metabolism	0	4	4	28	0.0169152	0.1039079
Cyanoamino acid metabolism	Metabolism	Metabolism of other amino acids	0	5	5	45	0.0217831	0.1248898
Histidine metabolism	Metabolism	Amino acid metabolism	2	3	5	47	0.0258266	0.1345057
Carbapenem biosynthesis	Metabolism	Biosynthesis of other secondary metabolites	1	3	4	32	0.0265883	0.1345057
Cysteine and methionine metabolism	Metabolism	Amino acid metabolism	1	5	6	67	0.0325138	0.1459427
Biosynthesis of various other secondary metabolites	Metabolism	Biosynthesis of other secondary metabolites	1	5	6	67	0.0325138	0.1459427
Lysine biosynthesis	Metabolism	Amino acid metabolism	1	3	4	35	0.0356372	0.1459427
Phenylalanine, tyrosine and tryptophan biosynthesis	Metabolism	Amino acid metabolism	2	2	4	35	0.0356372	0.1459427
Nicotinate and nicotinamide metabolism	Metabolism	Metabolism of cofactors and vitamins	2	3	5	55	0.0466322	0.1822896
Phenylpropanoid biosynthesis	Metabolism	Biosynthesis of other secondary metabolites	3	2	5	58	0.0564182	0.2109548
Diterpenoid biosynthesis	Metabolism	Metabolism of terpenoids and polyketides	1	7	8	124	0.0773389	0.2622042
Pyrimidine metabolism	Metabolism	Nucleotide metabolism	0	5	5	64	0.079271	0.2622042
Glyoxylate and dicarboxylate metabolism	Metabolism	Carbohydrate metabolism	1	4	5	64	0.079271	0.2622042
Isoquinoline alkaloid	Metabolism	Biosynthesis of other	5	3	8	129	0.092322	0.294063

biosynthesis		secondary metabolites					2	3
Phenylalanine metabolism	Metabolism	Amino acid metabolism	2	2	4	49	0.098434	0.3012239
Arginine and proline metabolism	Metabolism	Amino acid metabolism	1	4	5	69	0.1015755	0.3012239
beta-Alanine metabolism	Metabolism	Metabolism of other amino acids	0	3	3	32	0.10637	0.3049274
C5-Branched dibasic acid metabolism	Metabolism	Carbohydrate metabolism	0	3	3	35	0.1300554	0.3607988
Lysine degradation	Metabolism	Amino acid metabolism	1	3	4	56	0.1414047	0.3726065
Glucosinolate biosynthesis	Metabolism	Biosynthesis of other secondary metabolites	1	4	5	77	0.1429769	0.3726065
Nitrogen metabolism	Metabolism	Energy metabolism	0	2	2	19	0.1479636	0.3742609
Citrate cycle (TCA cycle)	Metabolism	Carbohydrate metabolism	0	2	2	20	0.1607009	0.3921872
Monobactam biosynthesis	Metabolism	Biosynthesis of other secondary metabolites	1	2	3	39	0.1641714	0.3921872
Monoterpenoid biosynthesis	Metabolism	Metabolism of terpenoids and polyketides	2	2	4	61	0.1759053	0.408861
Biosynthesis of secondary metabolites	Metabolism	Global and overview maps	22	66	88	2273	0.1930744	0.4369578
Biosynthesis of cofactors	Metabolism	Global and overview maps	5	10	15	328	0.2005796	0.4423037
Taurine and hypotaurine metabolism	Metabolism	Metabolism of other amino acids	0	2	2	24	0.2132704	0.4585313
Galactose metabolism	Metabolism	Carbohydrate metabolism	0	3	3	46	0.2290562	0.4804593

Limonene degradation	Metabolism	Metabolism of terpenoids and polyketides	0	3	3	47	0.2387098	0.4887868
Ubiquinone and other terpenoid-quinone biosynthesis	Metabolism	Metabolism of cofactors and vitamins	3	1	4	71	0.251919	0.503838
Flavone and flavonol biosynthesis	Metabolism	Biosynthesis of other secondary metabolites	2	1	3	51	0.2779265	0.5248015
Brassinosteroid biosynthesis	Metabolism	Metabolism of terpenoids and polyketides	0	2	2	29	0.2807078	0.5248015
Phosphatidylinositol signaling system	Environmental Information Processing	Signal transduction	0	2	2	29	0.2807078	0.5248015
Pantothenate and CoA biosynthesis	Metabolism	Metabolism of cofactors and vitamins	0	2	2	30	0.294213	0.5383472
Arachidonic acid metabolism	Metabolism	Lipid metabolism	1	3	4	79	0.3168697	0.563571
Pyruvate metabolism	Metabolism	Carbohydrate metabolism	0	2	2	32	0.3211044	0.563571
Neomycin, kanamycin and gentamicin biosynthesis	Metabolism	Biosynthesis of other secondary metabolites	0	4	4	81	0.3333821	0.5656037

Total, the total number of metabolites in the target metabolic pathway;

Pvalue, the p value of the hypergeometric distribution test;

FDR, corrected for false positives;

Pathway, metabolite metabolism pathway ID.
