# Supplementary material for: Chemical Composition Analysis of Highland Barley (Hordeum vulgare L.) with Different Modification Methods and Lipid Metabolism Mechanism Analysis of Highland Barley with Microwave Fluidization Modification
Source: Foods. 2026 Apr 17;15(8):1396. doi: 10.3390/foods15081396 (PMC13114515; doi:10.3390/foods15081396)
Supplement: Supplementary file 1 [file foods-15-01396-s001.zip › Table S7.pdf]

Table S7 The top 50 significant KEGG pathways between HB and HB-2.

Pathway	level1	level2	Up	Down	DEM	Total	Pvalue	FDR
Arginine biosynthesis	Metabolism	Amino acid metabolism	4	4	8	23	2.19E-05	0.002172
Biosynthesis of amino acids	Metabolism	Global and overview maps	8	10	18	128	0.000265	0.010704
Phenylpropanoid biosynthesis	Metabolism	Biosynthesis of other secondary metabolites	6	5	11	58	0.000324	0.010704
ABC transporters	Environmental Information Processing	Membrane transport	11	7	18	138	0.000675	0.016702
Flavone and flavonol biosynthesis	Metabolism	Biosynthesis of other secondary metabolites	6	3	9	51	0.001927	0.038162
Cutin, suberine and wax biosynthesis	Metabolism	Lipid metabolism	4	2	6	27	0.003335	0.048703
Plant hormone signal transduction	Environmental Information Processing	Signal transduction	0	4	4	12	0.003444	0.048703
Tyrosine metabolism	Metabolism	Amino acid metabolism	9	2	11	78	0.004048	0.050088
Diterpenoid biosynthesis	Metabolism	Metabolism of terpenoids and polyketides	7	7	14	124	0.009656	0.106218
alpha-Linolenic acid metabolism	Metabolism	Lipid metabolism	2	5	7	44	0.010793	0.106846
Arginine and proline metabolism	Metabolism	Amino acid metabolism	5	4	9	69	0.014771	0.121861
D-Amino acid metabolism	Metabolism	Metabolism of other amino acids	5	4	9	69	0.014771	0.121861
Glutathione metabolism	Metabolism	Metabolism of other	2	4	6	38	0.018479	0.125649

		amino acids						
Linoleic acid metabolism	Metabolism	Lipid metabolism	4	1	5	28	0.018713	0.125649
Phenylalanine metabolism	Metabolism	Amino acid metabolism	4	3	7	49	0.019038	0.125649
Aminoacyl-tRNA biosynthesis	Genetic Information Processing	Translation	3	4	7	52	0.025695	0.152993
Purine metabolism	Metabolism	Nucleotide metabolism	7	4	11	101	0.026272	0.152993
Isoquinoline alkaloid biosynthesis	Metabolism	Biosynthesis of other secondary metabolites	7	6	13	129	0.029361	0.161484
Carbapenem biosynthesis	Metabolism	Biosynthesis of other secondary metabolites	4	1	5	32	0.03193	0.166375
Metabolic pathways	Metabolism	Global and overview maps	94	94	188	3063	0.033731	0.166968
Betalain biosynthesis	Metabolism	Biosynthesis of other secondary metabolites	3	1	4	23	0.037771	0.178063
Phenylalanine, tyrosine and tryptophan biosynthesis	Metabolism	Amino acid metabolism	2	3	5	35	0.04491	0.193307
C5-Branched dibasic acid metabolism	Metabolism	Carbohydrate metabolism	1	4	5	35	0.04491	0.193307
Histidine metabolism	Metabolism	Amino acid metabolism	3	3	6	47	0.047203	0.194713
Pyrimidine metabolism	Metabolism	Nucleotide metabolism	2	5	7	64	0.067624	0.267309
Alanine, aspartate and glutamate metabolism	Metabolism	Amino acid metabolism	0	4	4	28	0.070202	0.267309
Brassinosteroid biosynthesis	Metabolism	Metabolism of terpenoids and polyketides	3	1	4	29	0.077979	0.280776
Cysteine and methionine metabolism	Metabolism	Amino acid metabolism	1	6	7	67	0.082248	0.280776
Biosynthesis of various other secondary metabolites	Metabolism	Biosynthesis of other secondary metabolites	1	6	7	67	0.082248	0.280776

Lysine degradation	Metabolism	Amino acid metabolism	3	3	6	56	0.094254	0.311039
Citrate cycle (TCA cycle)	Metabolism	Carbohydrate metabolism	0	3	3	20	0.10002	0.31942
Arachidonic acid metabolism	Metabolism	Lipid metabolism	5	2	7	79	0.157093	0.486006
Monobactam biosynthesis	Metabolism	Biosynthesis of other secondary metabolites	2	2	4	39	0.176103	0.52831
2-Oxocarboxylic acid metabolism	Metabolism	Global and overview maps	7	4	11	144	0.189499	0.543709
Carbon metabolism	Metabolism	Global and overview maps	3	6	9	114	0.19405	0.543709
Nicotinate and nicotinamide metabolism	Metabolism	Metabolism of cofactors and vitamins	3	2	5	55	0.197712	0.543709
Steroid biosynthesis	Metabolism	Lipid metabolism	0	5	5	57	0.218165	0.582194
Vitamin B6 metabolism	Metabolism	Metabolism of cofactors and vitamins	0	3	3	29	0.223567	0.582194
Pantothenate and CoA biosynthesis	Metabolism	Metabolism of cofactors and vitamins	2	1	3	30	0.238833	0.582194
Flavonoid biosynthesis	Metabolism	Biosynthesis of other secondary metabolites	5	1	6	74	0.238887	0.582194
Cyanoamino acid metabolism	Metabolism	Metabolism of other amino acids	1	3	4	45	0.248213	0.582194
Thiamine metabolism	Metabolism	Metabolism of cofactors and vitamins	1	2	3	31	0.25427	0.582194
Galactose metabolism	Metabolism	Carbohydrate metabolism	2	2	4	46	0.26083	0.582194
Biosynthesis of various antibiotics	Metabolism	Biosynthesis of other secondary metabolites	5	3	8	108	0.265178	0.582194
beta-Alanine metabolism	Metabolism	Metabolism of other amino acids	2	1	3	32	0.269843	0.582194

Limonene degradation	Metabolism	Metabolism of terpenoids and polyketides	2	2	4	47	0.273568	0.582194
Glycine, serine and threonine metabolism	Metabolism	Amino acid metabolism	1	3	4	48	0.286409	0.582194
Nitrogen metabolism	Metabolism	Energy metabolism	0	2	2	19	0.292087	0.582194
Glyoxylate and dicarboxylate metabolism	Metabolism	Carbohydrate metabolism	0	5	5	64	0.294038	0.582194
Isoflavonoid biosynthesis	Metabolism	Biosynthesis of other secondary metabolites	3	2	5	64	0.294038	0.582194

Total, the total number of metabolites in the target metabolic pathway;

Pvalue, the p value of the hypergeometric distribution test;

FDR, corrected for false positives;

Pathway, metabolite metabolism pathway ID.
