# Supplementary material for: Chemical Composition Analysis of Highland Barley (Hordeum vulgare L.) with Different Modification Methods and Lipid Metabolism Mechanism Analysis of Highland Barley with Microwave Fluidization Modification
Source: Foods. 2026 Apr 17;15(8):1396. doi: 10.3390/foods15081396 (PMC13114515; doi:10.3390/foods15081396)
Supplement: Supplementary file 1 [file foods-15-01396-s001.zip › Table S8.pdf]

Table S7 The top 50 significant KEGG pathways between HB and HB-3.

Pathway	level1	level2	Up	Down	DEM	Total	Pvalue	FDR
Arginine biosynthesis	Metabolism	Amino acid metabolism	4	3	7	23	0.000179	0.017348
Flavone and flavonol biosynthesis	Metabolism	Biosynthesis of other secondary metabolites	9	1	10	51	0.00042	0.020375
Tyrosine metabolism	Metabolism	Amino acid metabolism	10	2	12	78	0.001155	0.037344
alpha-Linolenic acid metabolism	Metabolism	Lipid metabolism	7	1	8	44	0.002627	0.061304
Cutin, suberine and wax biosynthesis	Metabolism	Lipid metabolism	4	2	6	27	0.00316	0.061304
Linoleic acid metabolism	Metabolism	Lipid metabolism	2	4	6	28	0.003837	0.062028
Biosynthesis of amino acids	Metabolism	Global and overview maps	11	4	15	128	0.004821	0.066803
Arginine and proline metabolism	Metabolism	Amino acid metabolism	9	0	9	69	0.013843	0.140188
D-Amino acid metabolism	Metabolism	Metabolism of other amino acids	6	3	9	69	0.013843	0.140188
Phenylpropanoid biosynthesis	Metabolism	Biosynthesis of other secondary metabolites	7	1	8	58	0.014452	0.140188
Vitamin B6 metabolism	Metabolism	Metabolism of cofactors and vitamins	2	3	5	29	0.020713	0.180868
Citrate cycle (TCA cycle)	Metabolism	Carbohydrate metabolism	1	3	4	20	0.02277	0.180868
Metabolic pathways	Metabolism	Global and overview maps	149	38	187	3063	0.025719	0.180868
Plant hormone signal transduction	Environmental Information Processing	Signal transduction	3	0	3	12	0.026105	0.180868
Nicotinate and nicotinamide	Metabolism	Metabolism of cofactors	5	2	7	55	0.032128	0.207761

metabolism		and vitamins						
Betalain biosynthesis	Metabolism	Biosynthesis of other secondary metabolites	4	0	4	23	0.036511	0.211896
Lysine biosynthesis	Metabolism	Amino acid metabolism	4	1	5	35	0.043194	0.211896
C5-Branched dibasic acid metabolism	Metabolism	Carbohydrate metabolism	2	3	5	35	0.043194	0.211896
Diterpenoid biosynthesis	Metabolism	Metabolism of terpenoids and polyketides	11	1	12	124	0.043265	0.211896
ABC transporters	Environmental Information Processing	Membrane transport	10	3	13	138	0.04369	0.211896
Flavonoid biosynthesis	Metabolism	Biosynthesis of other secondary metabolites	7	1	8	74	0.053005	0.229656
Phenylalanine metabolism	Metabolism	Amino acid metabolism	5	1	6	49	0.053682	0.229656
Purine metabolism	Metabolism	Nucleotide metabolism	8	2	10	101	0.054454	0.229656
2-Oxocarboxylic acid metabolism	Metabolism	Global and overview maps	11	2	13	144	0.05798	0.234337
Arachidonic acid metabolism	Metabolism	Lipid metabolism	6	2	8	79	0.072473	0.281197
Lysine degradation	Metabolism	Amino acid metabolism	5	1	6	56	0.090602	0.338017
Galactose metabolism	Metabolism	Carbohydrate metabolism	5	0	5	46	0.111884	0.401953
Histidine metabolism	Metabolism	Amino acid metabolism	4	1	5	47	0.119825	0.415107
Pentose phosphate pathway	Metabolism	Carbohydrate metabolism	2	2	4	36	0.139235	0.465717
Isoflavonoid biosynthesis	Metabolism	Biosynthesis of other secondary metabolites	4	2	6	64	0.145934	0.471853
Aminoacyl-tRNA biosynthesis	Genetic Information Processing	Translation	3	2	5	52	0.163222	0.510728
Monobactam biosynthesis	Metabolism	Biosynthesis of other secondary metabolites	4	0	4	39	0.171437	0.519669
Alanine, aspartate and	Metabolism	Amino acid metabolism	0	3	3	28	0.204084	0.599884

glutamate metabolism								
Steroid biosynthesis	Metabolism	Lipid metabolism	3	2	5	57	0.211912	0.604572
Biosynthesis of unsaturated fatty acids	Metabolism	Lipid metabolism	4	2	6	74	0.23148	0.632124
Cyanoamino acid metabolism	Metabolism	Metabolism of other amino acids	2	2	4	45	0.242231	0.632124
Thiamine metabolism	Metabolism	Metabolism of cofactors and vitamins	3	0	3	31	0.249167	0.632124
Carbapenem biosynthesis	Metabolism	Biosynthesis of other secondary metabolites	3	0	3	32	0.264528	0.632124
beta-Alanine metabolism	Metabolism	Metabolism of other amino acids	3	0	3	32	0.264528	0.632124
Pyruvate metabolism	Metabolism	Carbohydrate metabolism	1	2	3	32	0.264528	0.632124
Limonene degradation	Metabolism	Metabolism of terpenoids and polyketides	4	0	4	47	0.267186	0.632124
Pyrimidine metabolism	Metabolism	Nucleotide metabolism	4	1	5	64	0.286429	0.649205
Biosynthesis of cofactors	Metabolism	Global and overview maps	15	6	21	328	0.287792	0.649205
Phenylalanine, tyrosine and tryptophan biosynthesis	Metabolism	Amino acid metabolism	3	0	3	35	0.311161	0.685968
Tryptophan metabolism	Metabolism	Amino acid metabolism	6	0	6	83	0.318796	0.687183
Caffeine metabolism	Metabolism	Biosynthesis of other secondary metabolites	2	0	2	22	0.350693	0.738886
Glutathione metabolism	Metabolism	Metabolism of other amino acids	2	1	3	38	0.358017	0.738886
Carbon fixation in photosynthetic organisms	Metabolism	Energy metabolism	0	2	2	23	0.37129	0.750316
Biosynthesis of various antibiotics	Metabolism	Biosynthesis of other secondary metabolites	6	1	7	108	0.399999	0.777515

Pentose and glucuronate interconversions	Metabolism	Carbohydrate metabolism	3	1	4	58	0.408796	0.777515

Total, the total number of metabolites in the target metabolic pathway;

Pvalue, the p value of the hypergeometric distribution test;

FDR, corrected for false positives;

Pathway, metabolite metabolism pathway ID.
