# Supplementary material for: Chemical Composition Analysis of Highland Barley (Hordeum vulgare L.) with Different Modification Methods and Lipid Metabolism Mechanism Analysis of Highland Barley with Microwave Fluidization Modification
Source: Foods. 2026 Apr 17;15(8):1396. doi: 10.3390/foods15081396 (PMC13114515; doi:10.3390/foods15081396)
Supplement: Supplementary file 1 [file foods-15-01396-s001.zip › Table S9.pdf]

Table S9 The top 50 differential metabolites analysis among HB, HB-1, HB-2 and HB-3.

Name	pvalue	FDR	vip	HB-A	HB-B	HB-C	HB-1-A	HB-1-B	HB-1-C	HB-2-A	HB-2-B	HB-2-C	HB-3-A	HB-3-B	HB-3-C
Methylpyrrolidone	0.000173	0.000479	1.292721	1775823	1470100	6268315	8799426	8337097	10369273	4526668	3875693	3687709	19044772	17954933	20646348
Iron	0.000412	0.000941	0.911126	22112478	18451801	16069107	19379423	19529534	20231218	52060535	45674700	48185104	7936057	7828364	1755690
Fe^{2+}	0.000337	0.000798	0.908454	14047095	11551241	39471146	40961132	37579172	12488383	3.39E+08	2.03E+08	79855560	3789304	3680786	3229158
2-Methyl-3-oxopropanoic acid	4.46E-05	0.000154	0.965687	30176992	23480157	30663288	22710721	29800212	38725365	16456028	14727519	13998430	59651455	62699678	58207499
Cyclohexan	0.037892	0.04884	0.812906	10326265	67865292	54277476	18509652	9640681	2.9E+08	2771904	2500057	2343243	1.73E+08	12549264	1.24E+08
SCHEMBL534447	0.001776	0.003322	0.925421	68080477	70603448	86509811	1.14E+08	3.09E+08	39881532	3607704	3192754	5731677	2.48E+08	42360313	46292721
(S)-2-Azetidinecarboxylic acid	8.84E-06	4.32E-05	1.021029	1.34E+08	1.85E+08	2.3E+08	3.62E+08	80156373	2.56E+08	5038337	4427765	4294285	3.78E+08	2.85E+08	2.24E+08
Hexylamine	0.047915	0.060355	0.880639	1.42E+08	4.21E+08	1.23E+09	1.62E+09	8.59E+08	2.16E+09	1.16E+08	2615528	18579287	3.49E+08	3.32E+08	3.65E+08
1-Aminocyclopropanecarboxylic acid	0.00168	0.003165	1.532825	41540550	35421983	34301391	26264114	26916218	27042094	29917637	26753147	25782132	36007319	31347608	33066147
4,5-Dihydro-2-methylthiazole	0.053791	0.06716	1.036482	1620226	1276612	3086373	2716155	2490638	4280519	6225659	5012066	4848453	5270744	2097542	2305276
L-3-Aminodihydro-2(3H)-furanone	0.076493	0.092632	1.223498	3930164	3757646	3208134	4586707	10470203	4806525	7020745	6122152	5704845	3659879	4531953	4887864

Malonate	6.29E-08	1.09E-06	1.022776	10230199	8805565	7985406	6662928	7764925	7456165	7655020	6829432	6977594	29521956	29501086	28505884
alpha-Hydroxyisobutyric acid	0.00841	0.012462	0.885399	4341993	2773569	2371261	2736890	4371093	2013147	2549798	2068899	2207252	5778293	6086571	6306754
(卤)-2-Methylbutanal	0.000707	0.001529	0.83489	3868000	55570700	90545637	97242112	71904640	75269996	13523957	12400625	11699452	6365844	11800979	5007050
2-Pentanone	2.98E-07	3.18E-06	0.975665	3.13E+09	2.66E+09	2.61E+09	1.97E+09	1.91E+09	1.96E+09	7.56E+09	6.79E+09	6.04E+09	3.79E+09	3.56E+09	3.59E+09
2-Aminoisobutyric acid	2.87E-05	0.000111	1.457479	3.63E+08	3.11E+08	2.97E+08	1.78E+08	1.77E+08	1.88E+08	3.77E+08	3.35E+08	3.05E+08	2.39E+08	2.24E+08	2.27E+08
Cyclopropanecarboxylic acid	0.211151	0.234089	0.788943	42440756	2.13E+08	35734289	1.21E+08	1.2E+08	93421758	1.28E+08	5.31E+08	92989933	64108725	63183517	35229409
Styrene	0.000196	0.00053	1.430741	497578.3	442192.6	1777590	4986964	5619267	7101244	1521598	1314548	1204450	10217592	9614162	9870374
Glycerate	0.005366	0.00842	1.013911	13842851	11149760	9944945	12283681	11965615	13148529	15718513	12244513	10726159	19466365	20510650	18422209
FAL 8_1	0.021738	0.029623	0.919622	7394228	5175940	7005462	16631792	8116912	8380554	2323484	1980076	6487079	13793105	7619352	12265540
2-Oxazolidinone	1.07E-07	1.53E-06	1.031144	5.08E+09	3.5E+09	2.82E+09	1.72E+09	2.77E+09	2.13E+09	79212859	72891776	82540410	3.81E+08	2.82E+08	2.31E+08
aminophenol	0.00057	0.001278	1.294083	1586678	1277090	6732839	15403252	16015424	16109238	2477048	2054537	6014382	39065817	36120844	41792900
(R)-Sulcatol	0.004566	0.007477	1.114196	1401231	1276460	3496015	2893943	4357630	3010362	9062644	7739055	8006548	3280186	6138036	5565387
Barbituric acid	0.272213	0.296543	0.702771	7.18E+08	63372951	4.46E+08	2.98E+08	78822613	2.11E+08	1.5E+08	60694003	1.04E+08	37488107	2.04E+08	20620596
1,2-Cyclohexanedione	3.32E-05	0.000122	0.992943	1570436	1268102	1108268	1243925	1405130	1459647	583851.1	495262.2	587367.7	856828.2	914689.5	797080

2-Furoic acid	3.41E-07	3.75E-06	1.145686	10341410	11758564	7604824	3331589	3446674	3482164	11697188	15426584	9589999	29346423	29565899	28924992
Acetol phosphate	7.43E-05	0.000243	1.191245	5.42E+08	2.45E+08	2.32E+08	1.7E+08	69865967	1.52E+08	1.96E+08	1.86E+08	1.75E+08	34950023	30906067	29714415
Uracil	0.00011	0.000315	0.940999	14476675	3025124	2576259	2963855	1636622	1750591	2124728	1932265	1857922	9374691	10494642	8966681
Imidazole-4-acetaldehyde	8.05E-09	2.71E-07	1.530481	673934.1	517464	560047.1	4737373	6030449	5205654	1132771	905611.3	909781.3	6425315	6523694	6780656
Creatine	2.58E-07	3.08E-06	0.969486	1.28E+08	1.48E+08	1.12E+08	1.07E+08	2.8E+08	1.31E+08	539906.8	400362.5	443292.4	43917753	43692553	1.19E+08
3-Chlorophenol	2.50E-05	9.99E-05	0.999944	20135099	15552487	14152587	16638580	15045151	15343483	9360146	9733822	8881334	3384292	5357298	3114866
Dimethyldisulfide	0.001771	0.003313	1.092767	31404511	25212422	32809434	47065429	51353882	49872582	25077782	22677800	22382186	33273609	21495095	19342956
D-2-Amino-hexano-6-lactam	3.22E-06	1.94E-05	1.064648	12145900	9502998	9893238	8878424	8570292	9138359	9356235	8148063	7652212	22667664	20840516	23366549
Histamine	2.84E-07	3.08E-06	1.358055	665323.3	506862.6	538862.9	3799576	3816084	3569719	1217854	1075109	971378.8	20949099	11195402	11058663
1,1-Dichloroethylene epoxide	1.99E-05	8.24E-05	1.029427	10740467	7089521	7845874	12197496	9889767	12021491	1939795	1637466	1530684	26894271	11252780	18731205
Oxopent-4-enoate	1.40E-06	1.04E-05	1.009609	18781894	18447531	13515230	15367441	12896891	14609001	66507053	57522254	56710993	28487019	30960163	27537676
2,5-Dimethylthiazole	0.440682	0.464804	0.535678	25272873	23859972	39100879	25027661	32386842	20746855	33825320	19718810	47386823	26121722	1.02E+08	38136053
Creatinine	2.48E-05	9.91E-05	1.060585	1.41E+08	1.22E+08	1.17E+08	1.33E+08	1.94E+08	1.31E+08	58235670	53364443	78894828	5.03E+08	3.18E+08	5.11E+08
N-Acetyl-L-alanine	0.547856	0.569165	0.67211	83893926	71668800	4999124	6111177	4838204	75208885	5855181	5364162	9809460	8618625	7637856	58273116

Fumaric acid	7.22E-05	0.000226	1.039712	3.25E+08	2.79E+08	2.52E+08	2.74E+08	2.94E+08	2.94E+08	2E+08	1.74E+08	1.73E+08	1.82E+08	1.81E+08	1.77E+08
Glutarate semialdehyde	0.031276	0.040193	0.85236	1727367	1277227	1109672	936177.7	1789761	1710342	966564.9	1844915	839126.5	2615121	3338163	2538279
alpha-Ketoisovaleric acid	0.20428	0.223757	0.69622	31042497	2961102	11290096	8711077	27266912	11308427	1966608	6989913	2076680	1926436	1729686	13116694
Hydroxyisocaproic acid	0.060488	0.074691	1.278318	12380595	9946646	9232518	12541201	12792772	14179724	12362578	10673308	10517890	12829264	12215108	12620964
(卤)-2-Hydroxy-2-phenylacetonitrile	0.002794	0.004899	1.390949	15852796	25766751	11346614	576841	698516.7	725712.6	2420514	65980353	9097215	845174.2	3911470	2231332
Cyclohexanone	0.123337	0.142819	0.751063	2177846	21137367	4102177	1856246	1901060	1935609	6646746	4515109	23271852	2472852	41234356	17737893
3-hexenal	0.000381	0.000916	0.881774	14917102	4468789	8073021	6779220	6347142	6573295	26517822	23542700	22183288	6209018	5927108	11881638
1-Hydroxypyrrole-2,5-diol	0.024591	0.033015	1.170135	16031084	7177886	13717362	2431217	2174878	2243299	9257285	64552170	7720960	2546554	6098712	2448193
Trimethylglycine	0.00322	0.005377	0.968294	16437393	3112325	2813584	13768042	19061428	11719244	13483424	3986623	10734119	2095869	2213207	2094949
Guanidinoacetate	0.180569	0.199412	0.643276	3640150	3106797	11933631	3092845	23583727	11240501	28904973	3226910	2851447	2075283	2210805	2112021
L-2-Amino-3-oxobutanoic acid	0.000822	0.001667	0.930045	3287786	2091448	2247106	2285524	2684523	3179206	1591189	1440706	1414908	3797333	3635423	3274942

vip: OPLS-DA first principal component variable importance value projection, vip≥ 1.

foldChange: Ploidy change between two groups, foldChange ≥ 1.

log2 (foldChange): log2 value of ploidy change.

p-value: Statistically significant difference, *p*-value ≤ 0.05.

FDR: *P* value Correction value.
