# Supplementary material for: Chemical Composition Analysis of Highland Barley (Hordeum vulgare L.) with Different Modification Methods and Lipid Metabolism Mechanism Analysis of Highland Barley with Microwave Fluidization Modification
Source: Foods. 2026 Apr 17;15(8):1396. doi: 10.3390/foods15081396 (PMC13114515; doi:10.3390/foods15081396)
Supplement: Supplementary file 1 [file foods-15-01396-s001.zip › Uncropped, unprocessed and full gel and blot.pdf]

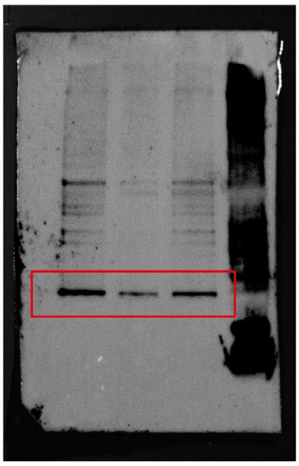
NCD
HFCD
HFCD+HB-1
ADIPOQ
41 kDa

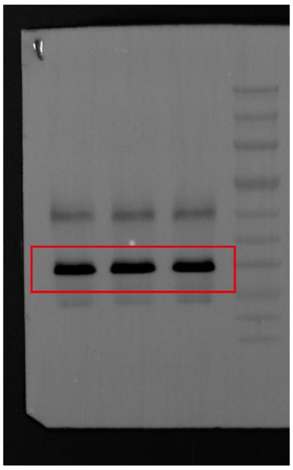
NCD
HFCD
HFCD+HB-1
GAPDH
37 kDa

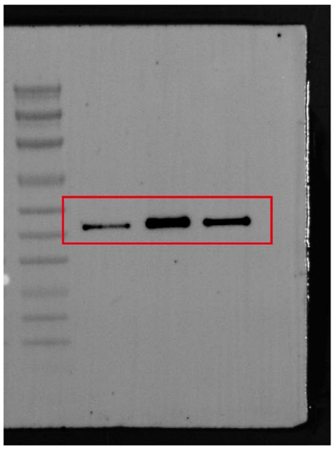
NCD
HFCD
HFCD+HB-1
26 kDa
SCD-1

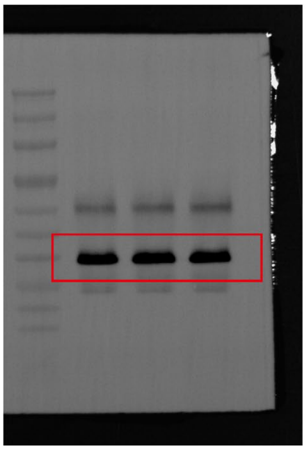
NCD
HFCD
HFCD+HB-1
37 kDa
GAPDH

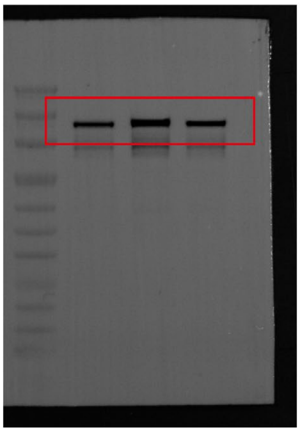
NCD
HFCD
HFCD+HB-1
122 kDa
SREBP-1c

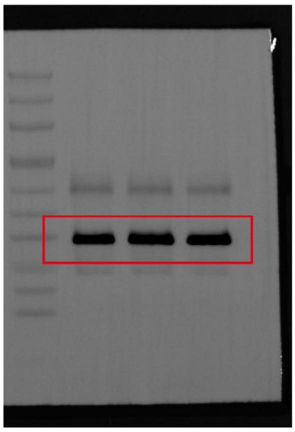
NCD
HFCD
HFCD+HB-1
37 kDa
GAPDH

NCD HFCD HFCD+HB-1

88 kDa

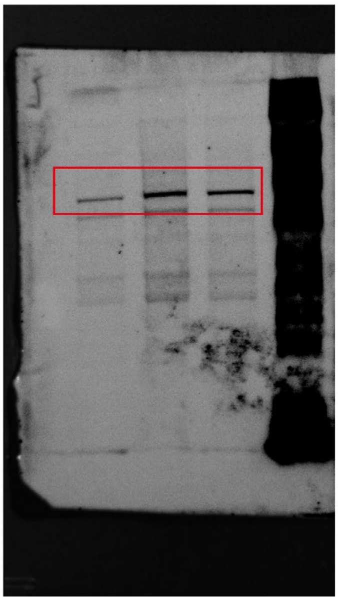

CD36

NCD HFCD HFCD+HB-1

GAPDH

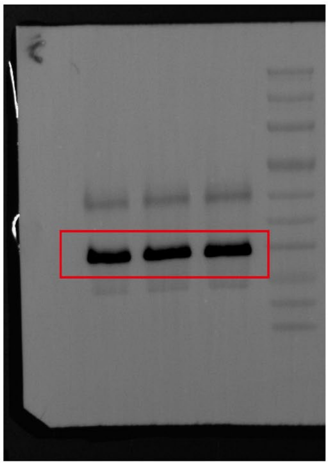

37 kDa

NCD HFCD HFCD+HB-1

15 kDa

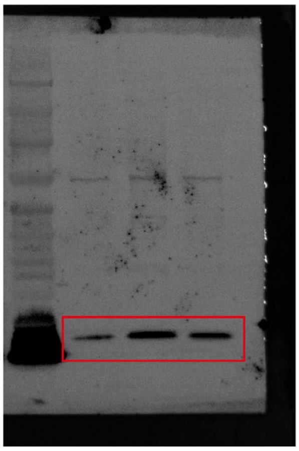

FABP4

NCD HFCD HFCD+HB-1

37 kDa

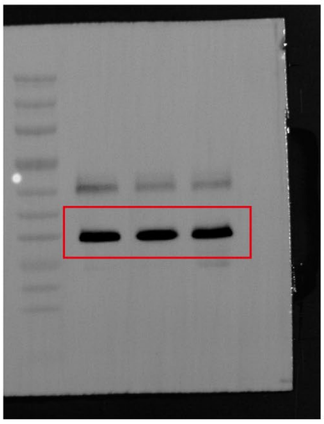

GAPDH

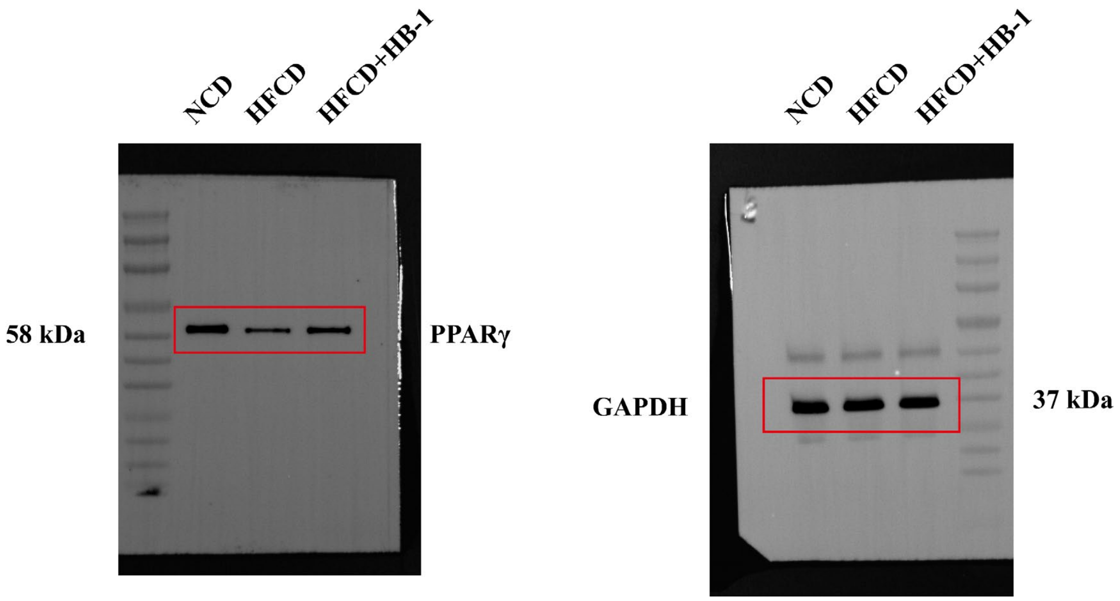

The uncropped, unprocessed and full gel and blot of PPARγ pathway including ADIPOQ, SCD-1, SREBP-1c, CD36 and FABP4.

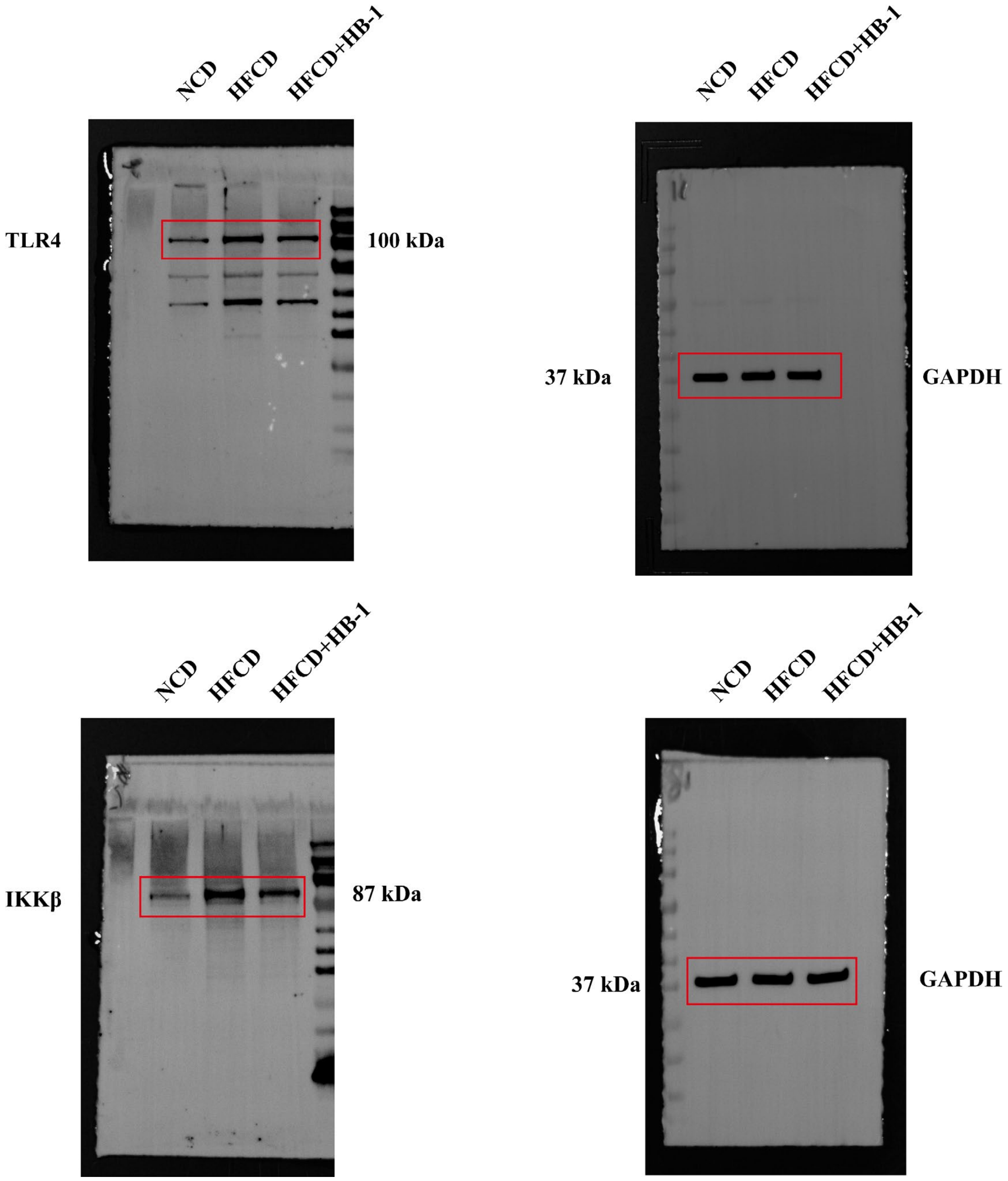
NCD
HFCD
HFCD+HB-1
TLR4
100 kDa
NCD
HFCD
HFCD+HB-1
37 kDa
GAPDH
NCD
HFCD
HFCD+HB-1
IKKβ
87 kDa
NCD
HFCD
HFCD+HB-1
37 kDa
GAPDH

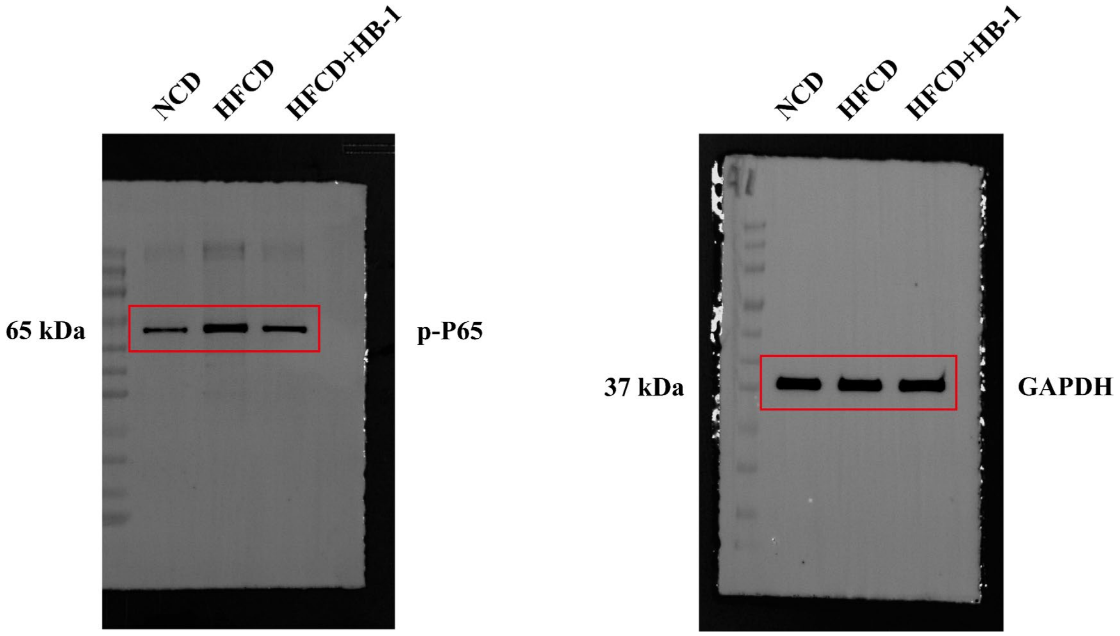

The uncropped, unprocessed and full gel and blot of TLR4/NF-κB pathway including TLR4, IKKβ and p-P65.
