# Supplementary material for: Chemical Composition Analysis of Highland Barley (Hordeum vulgare L.) with Different Modification Methods and Lipid Metabolism Mechanism Analysis of Highland Barley with Microwave Fluidization Modification
Source: Foods. 2026 Apr 17;15(8):1396. doi: 10.3390/foods15081396 (PMC13114515; doi:10.3390/foods15081396)

Western blot images results

PPARγ-1

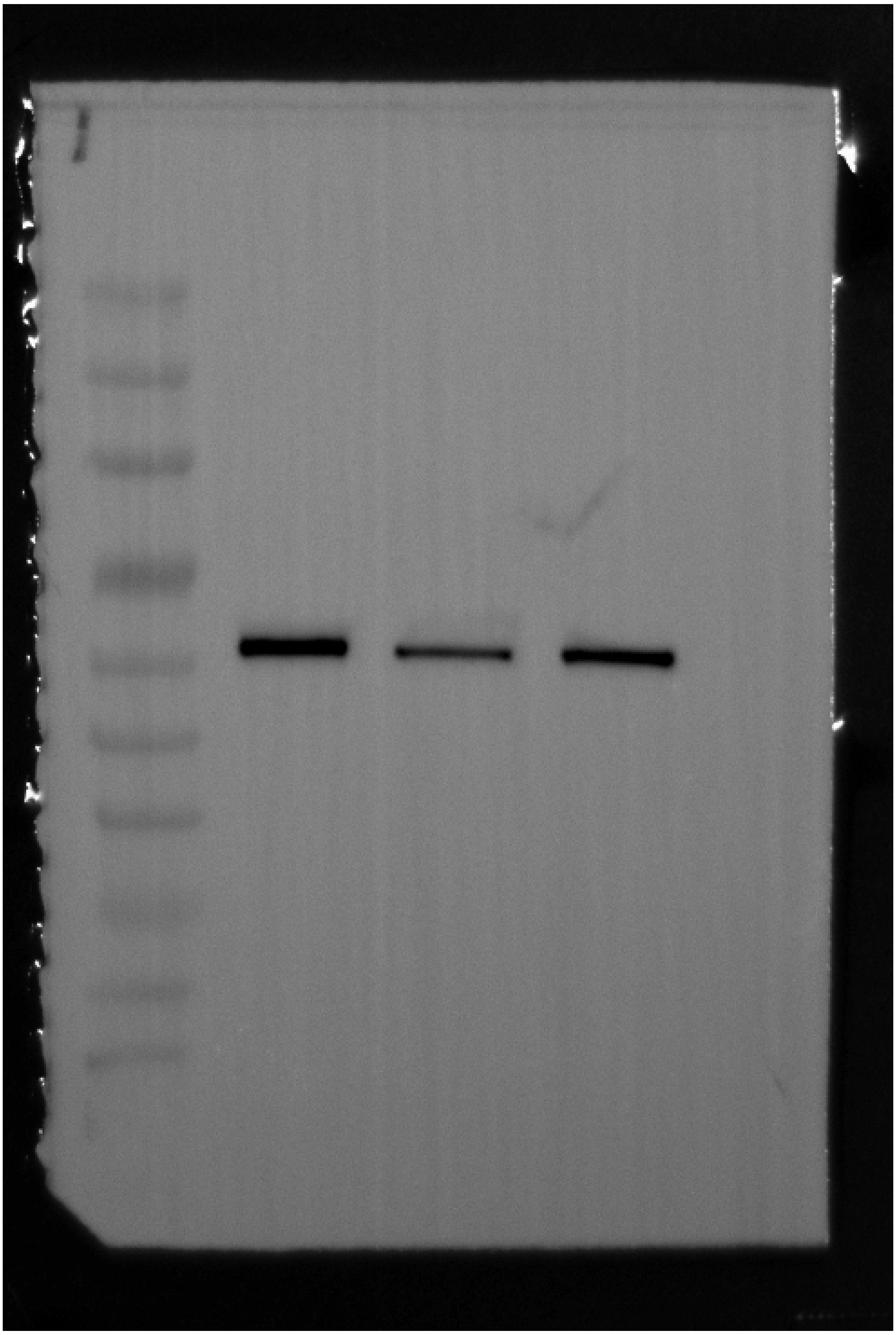

PPARγ-2

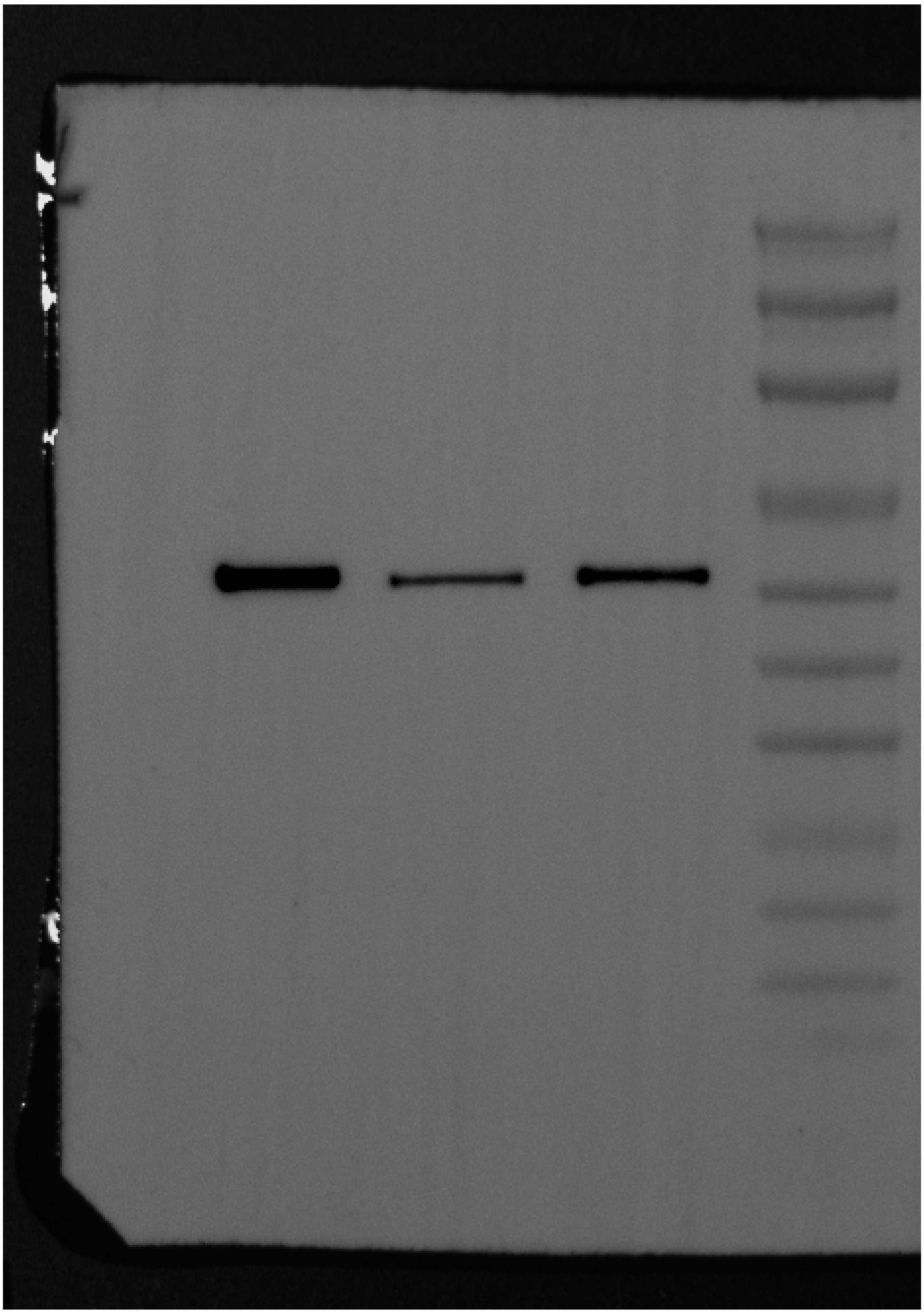

PPARγ-3

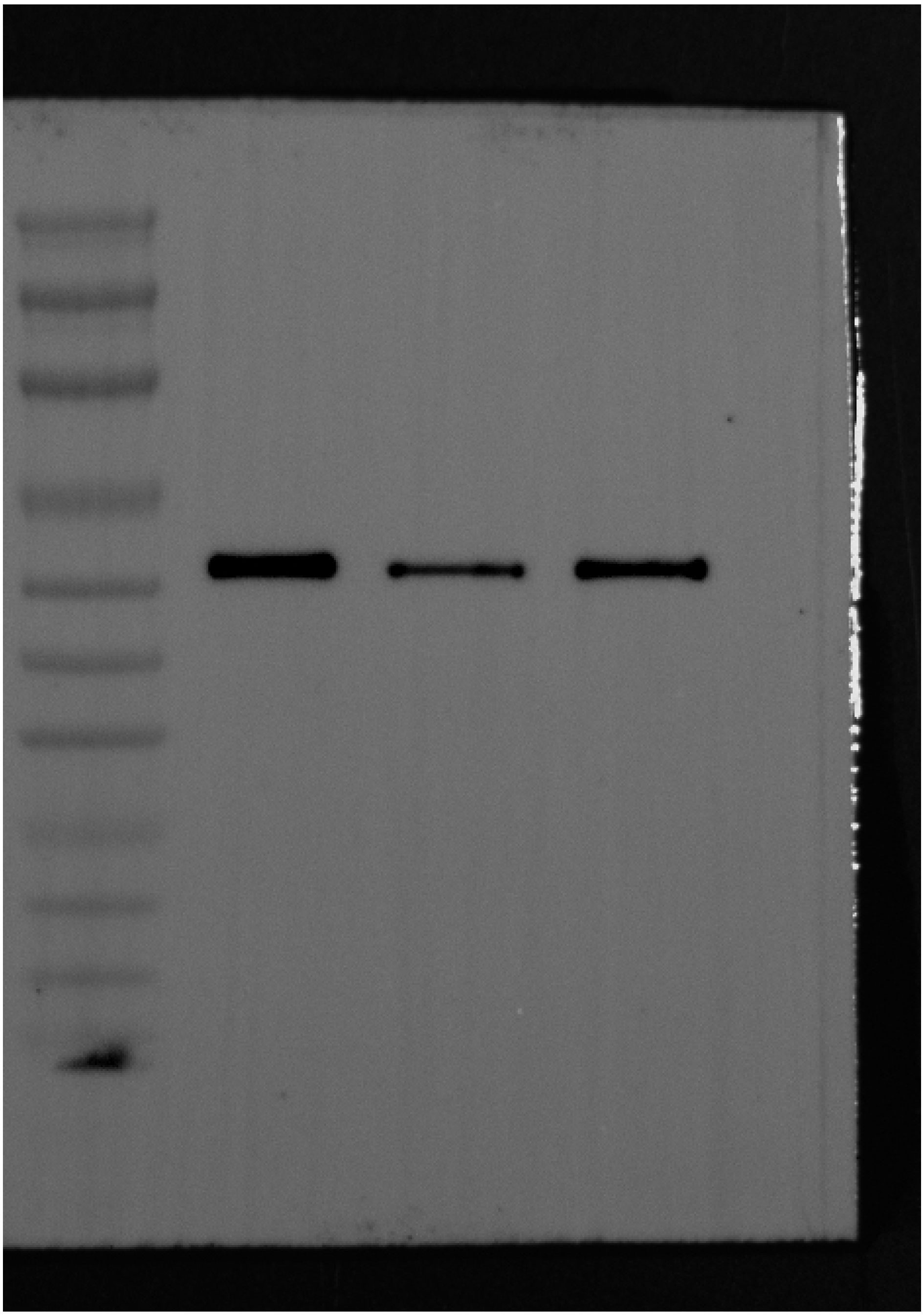

FABP4-1

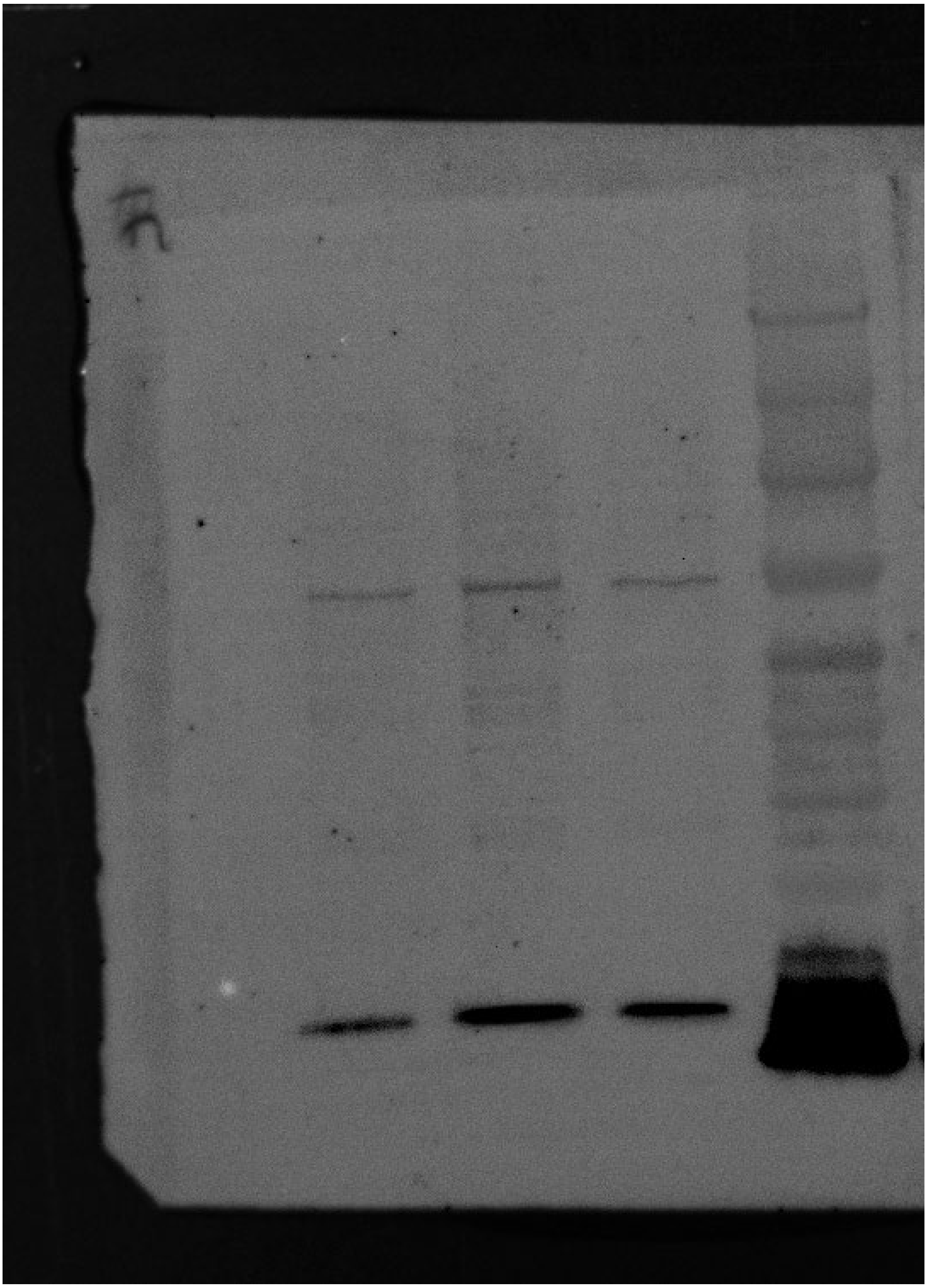

FABP4-2

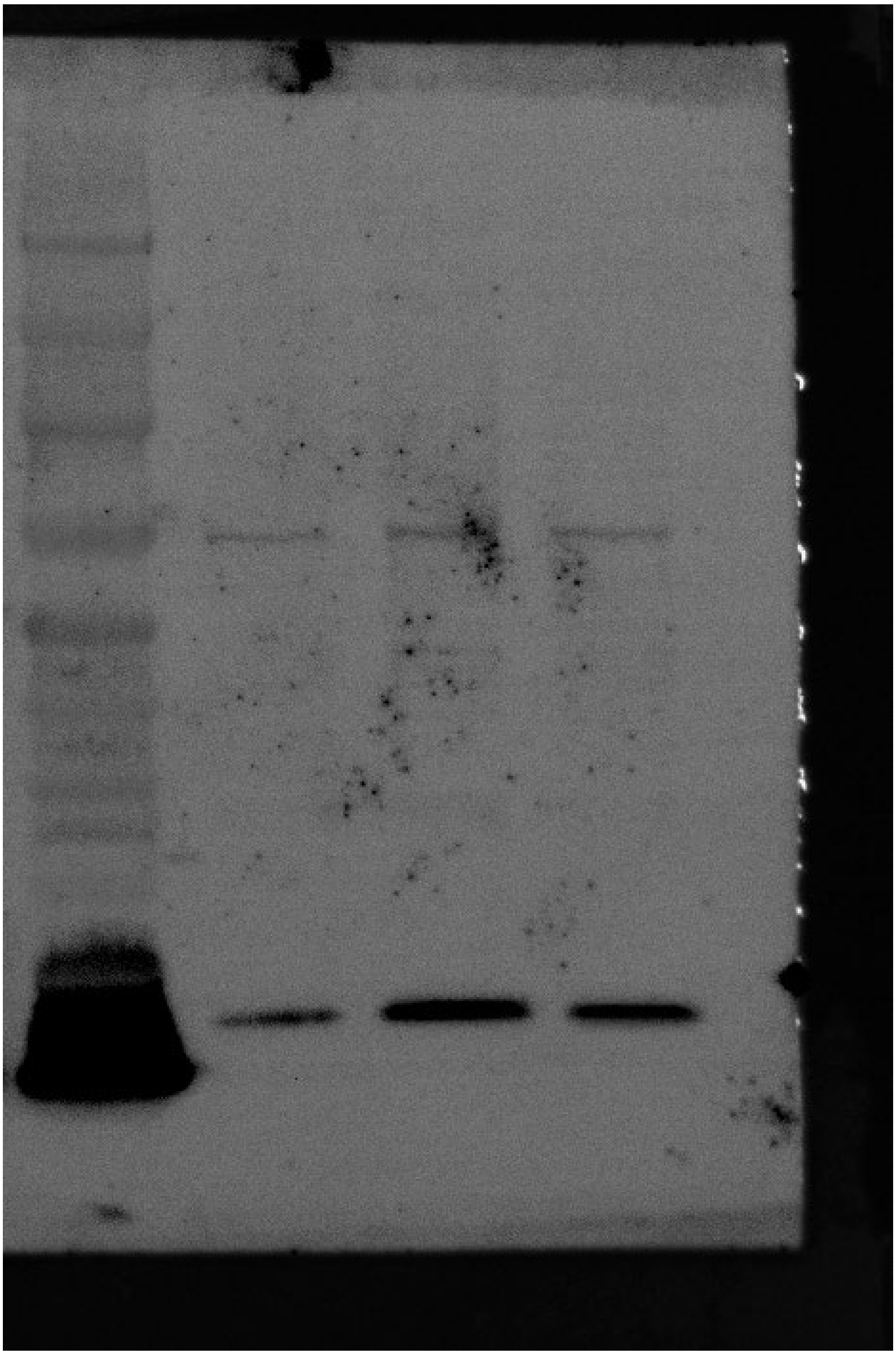

FABP4-3

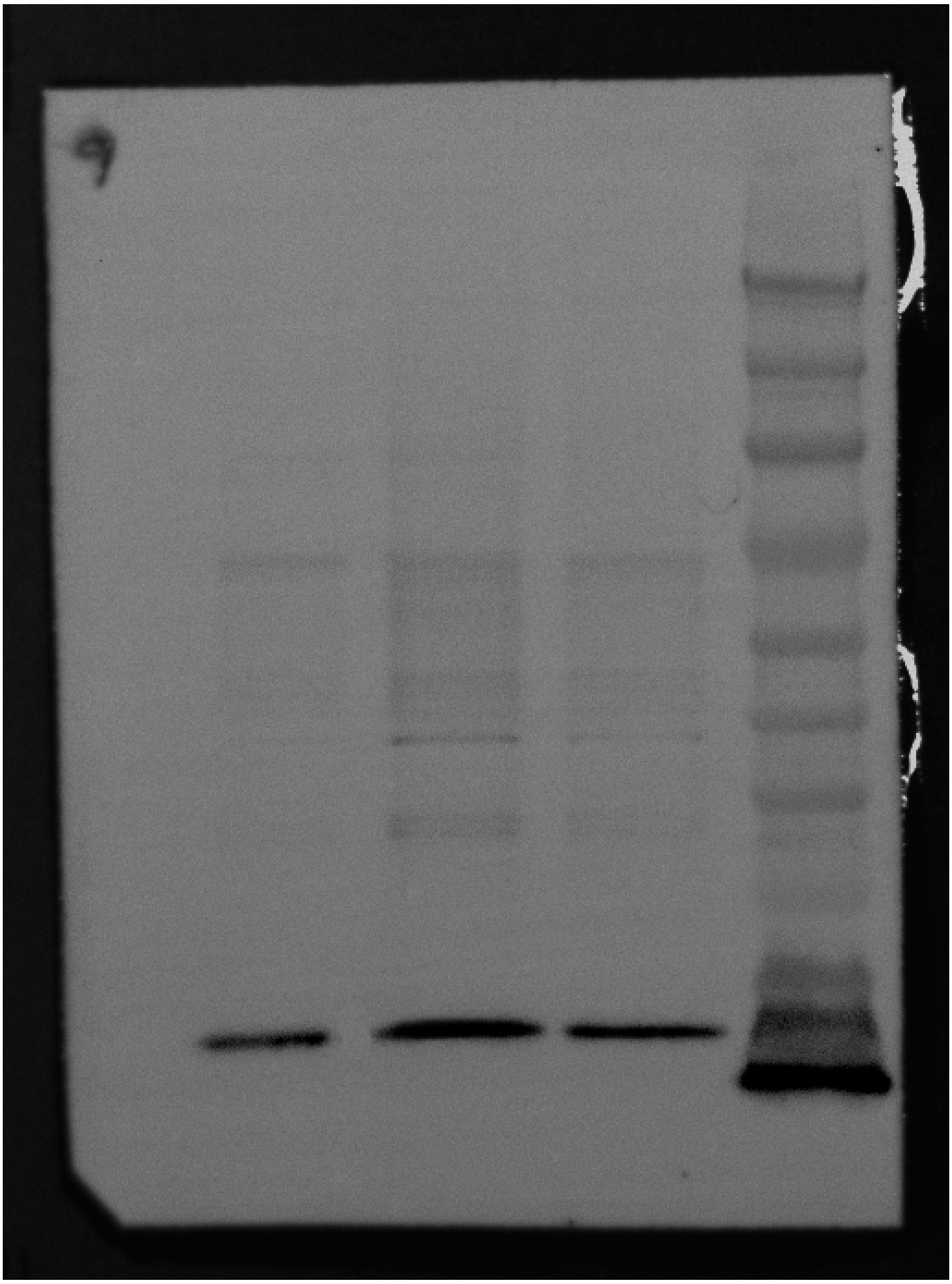

CD36-1

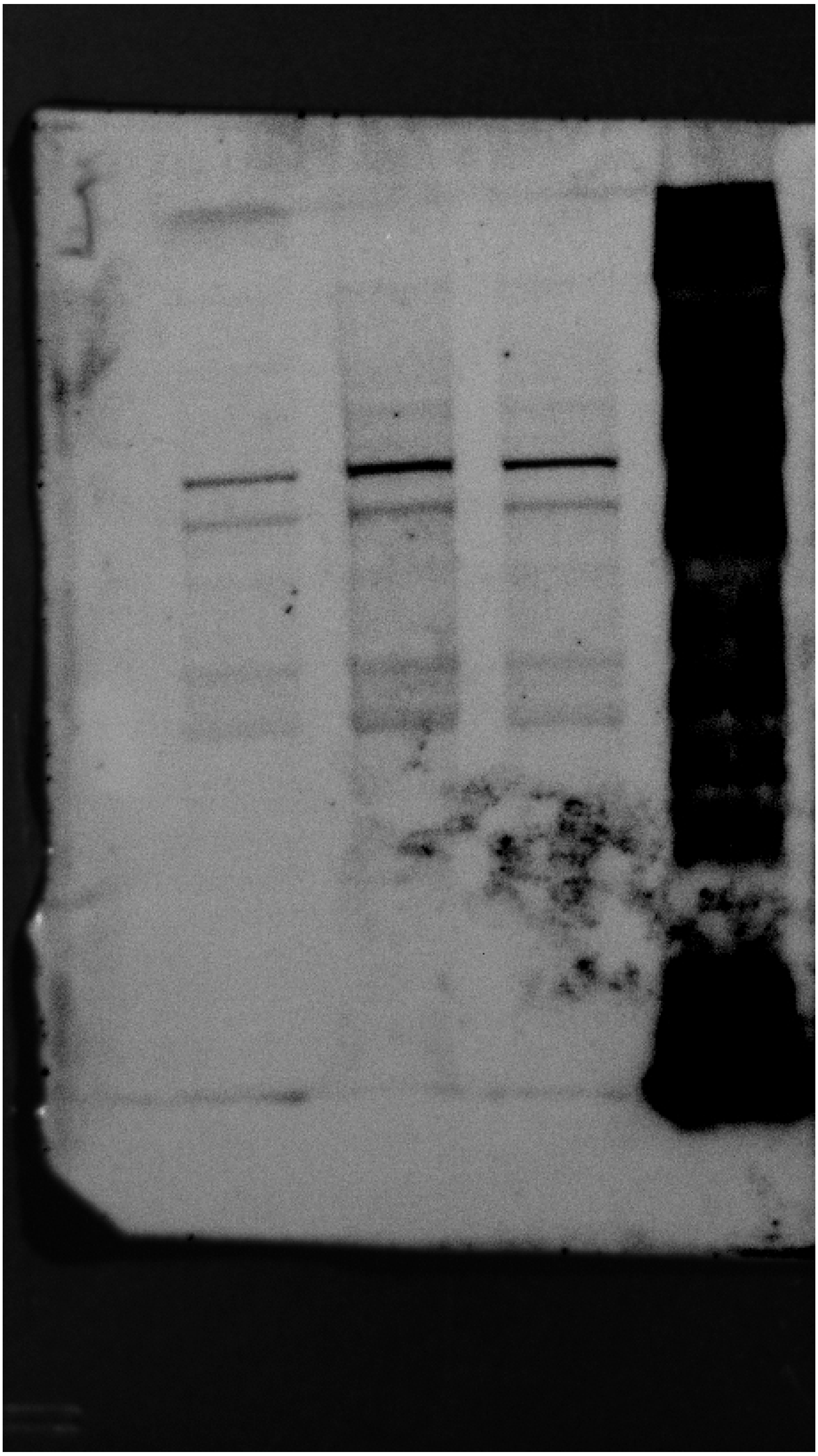

CD36-2

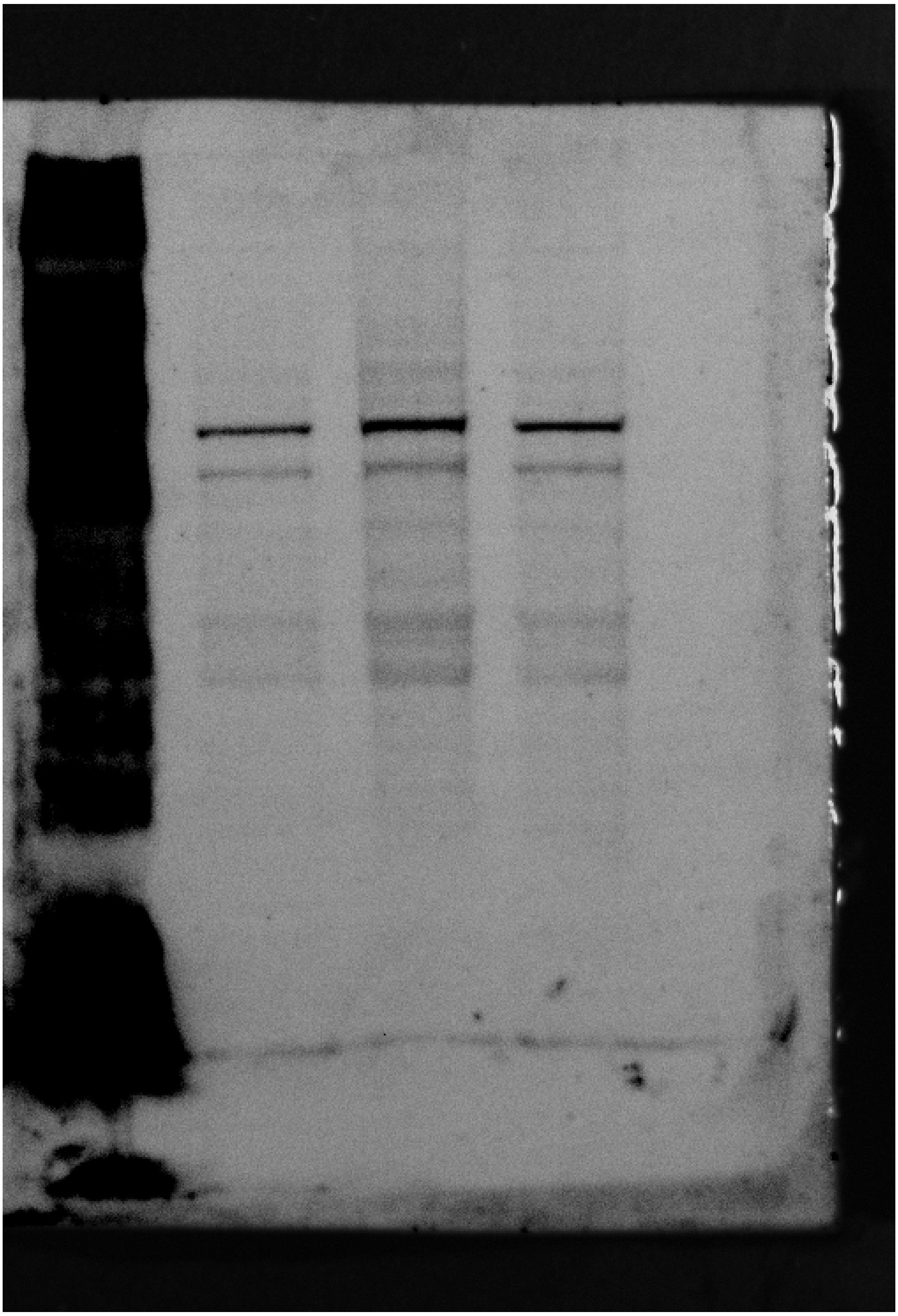

CD36-3

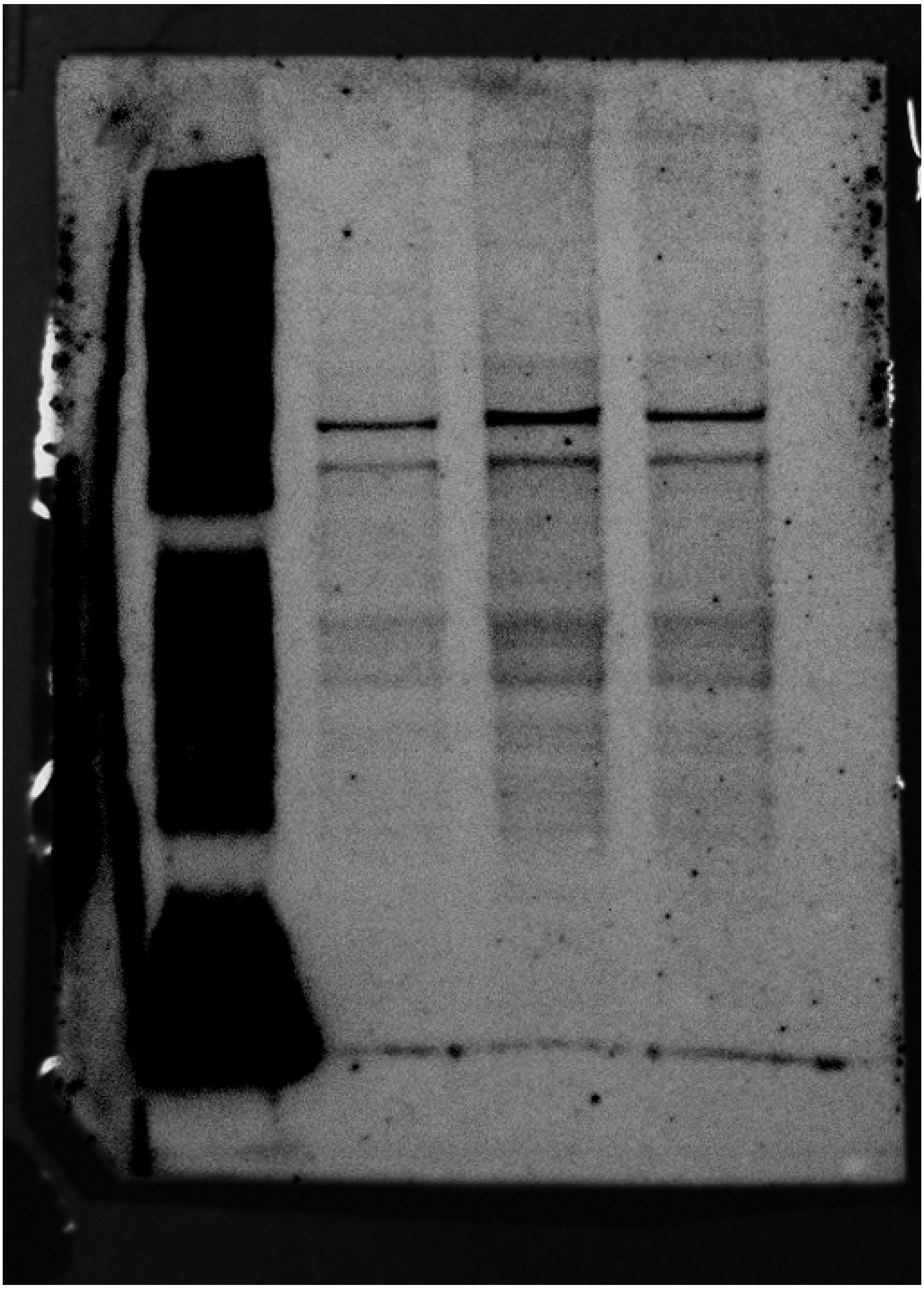

SREBP-1c-1

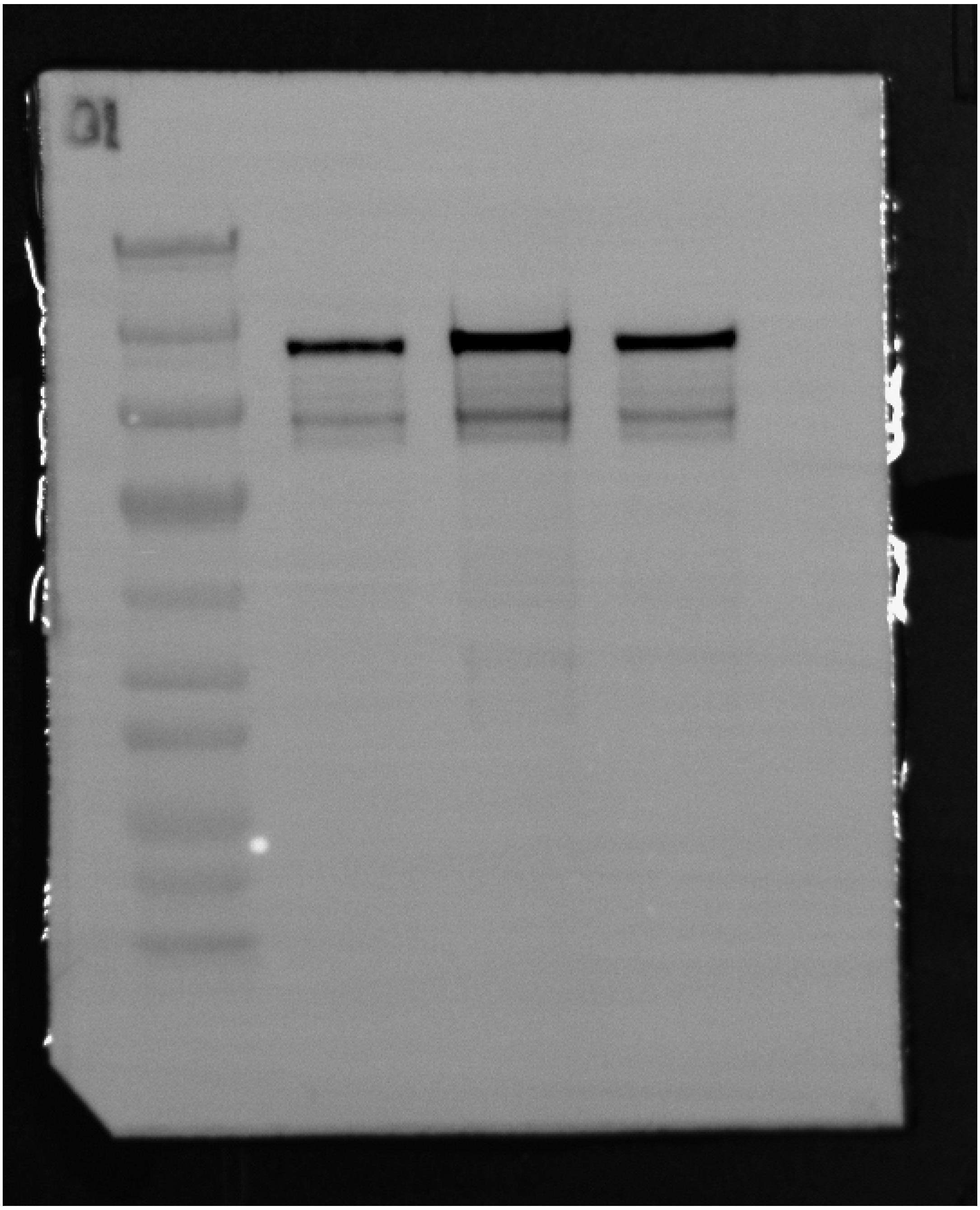

SREBP-1c-2

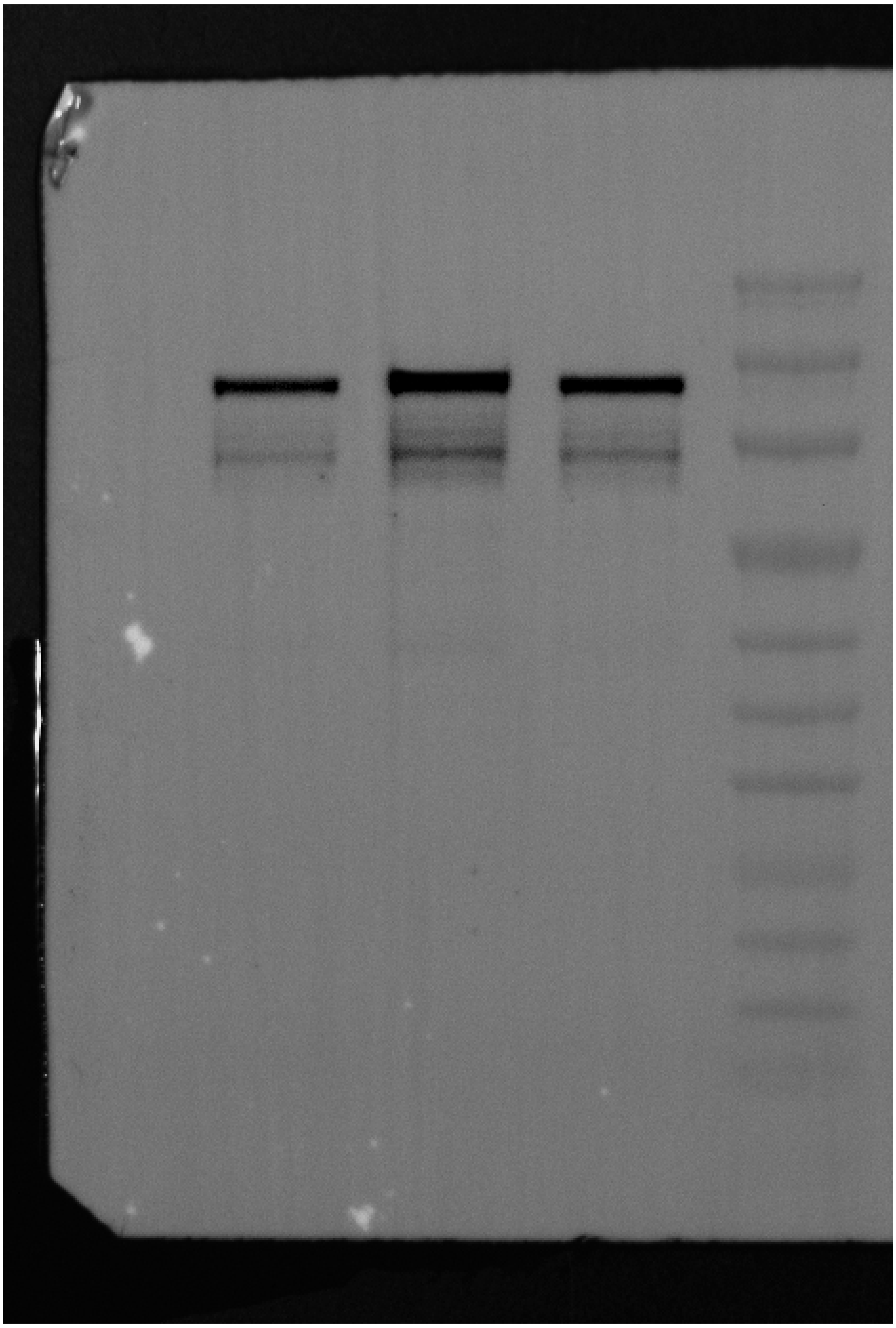

SREBP-1c-3

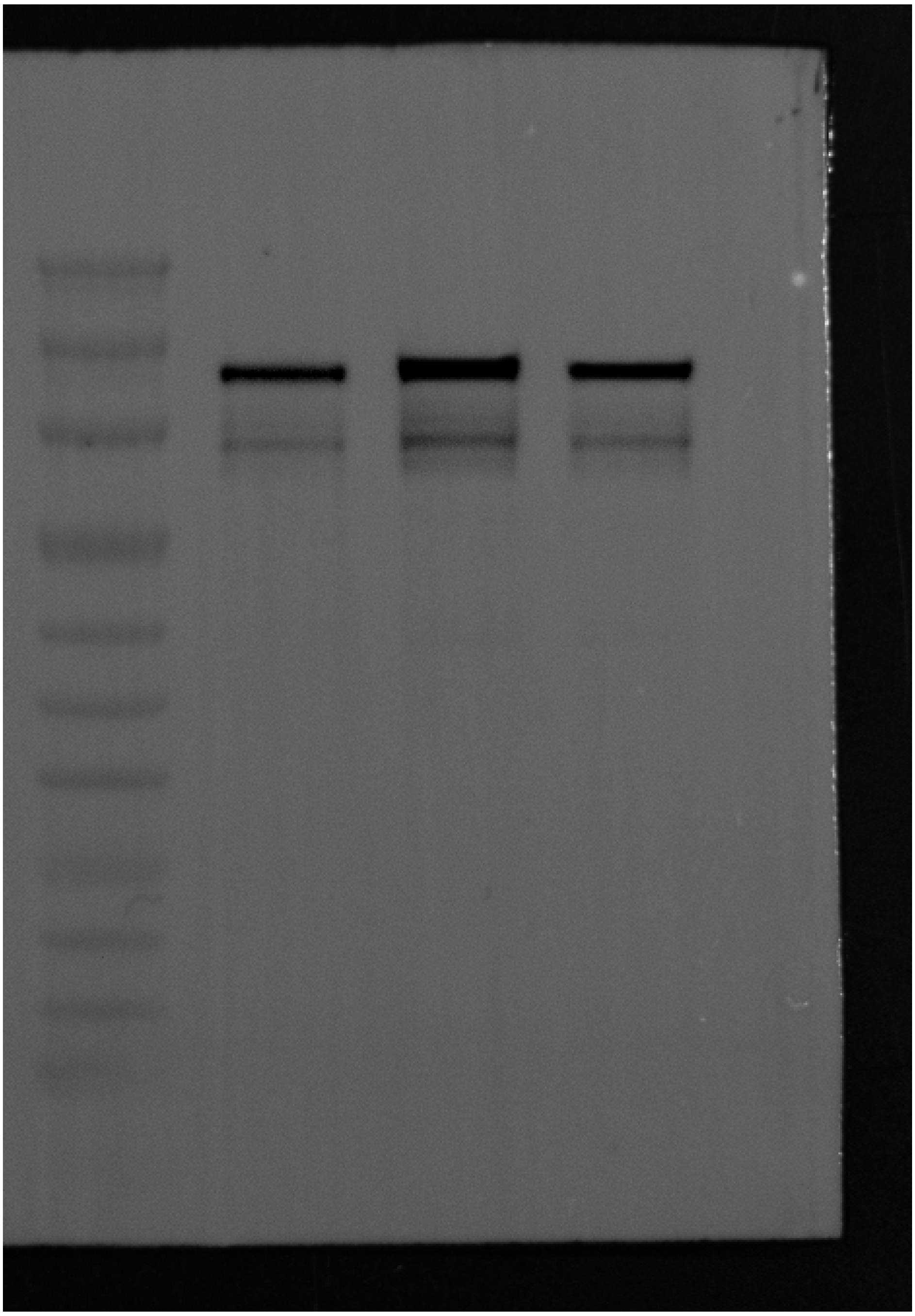

SCD-1-1

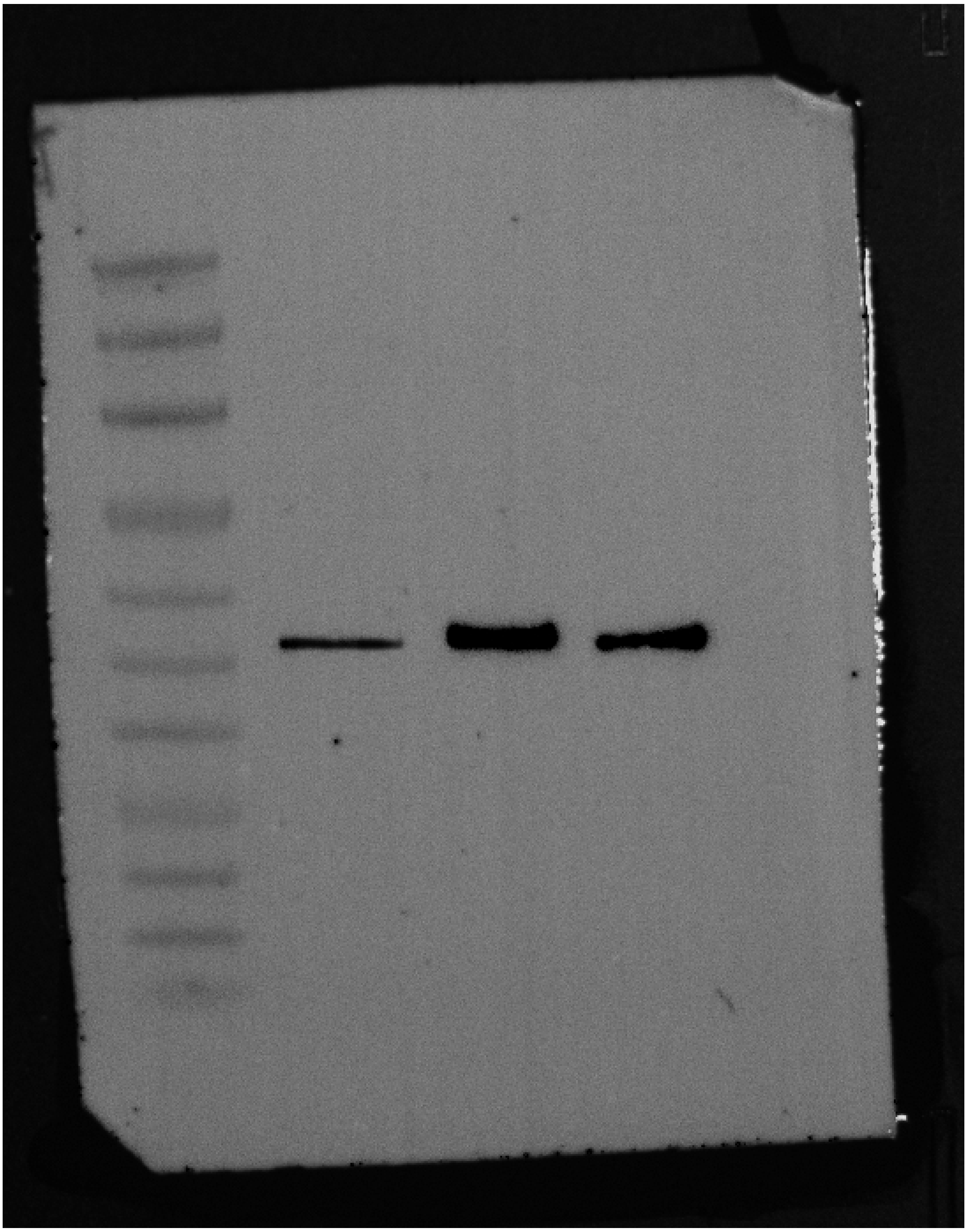

SCD-1-2

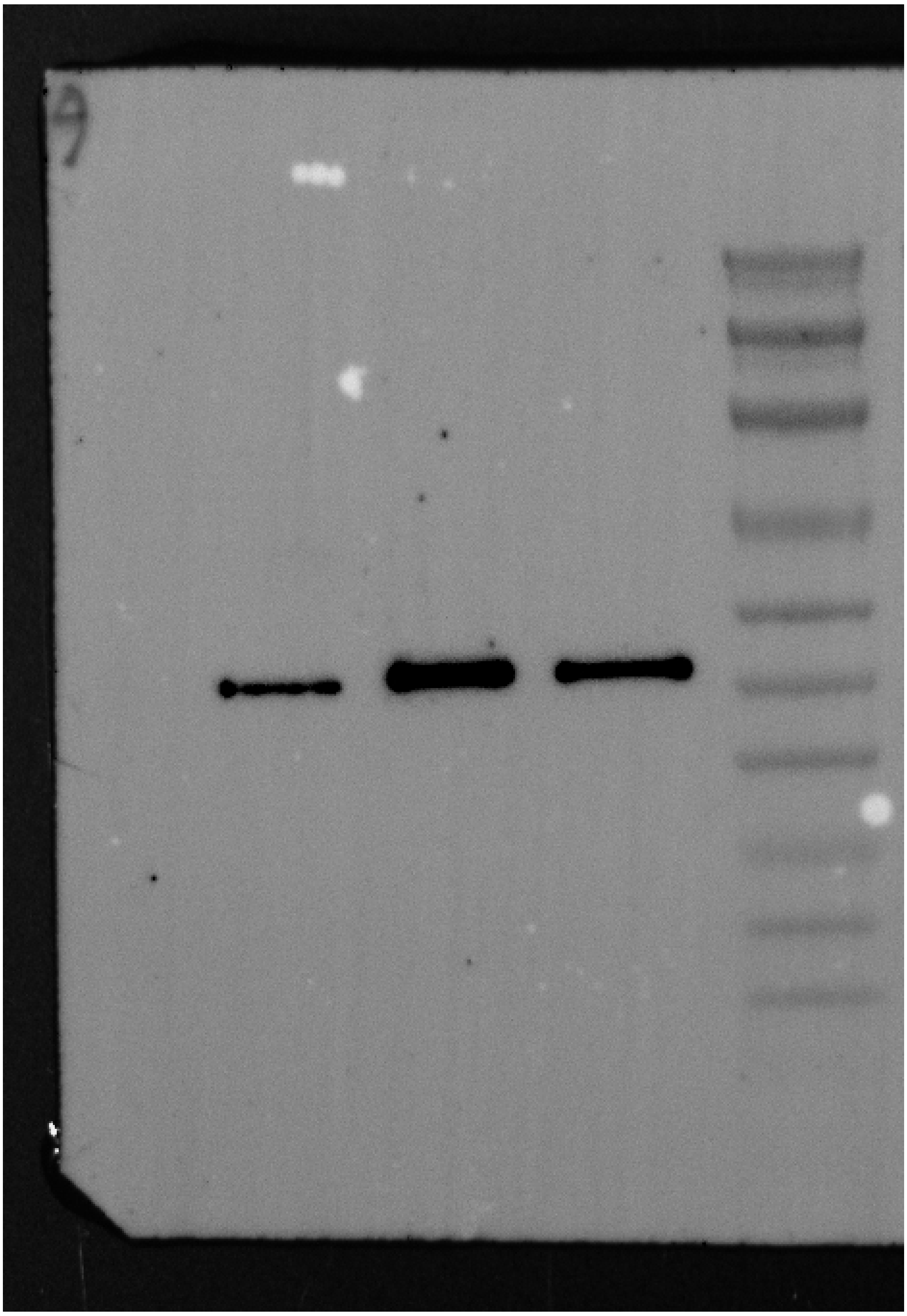

SCD-1-3

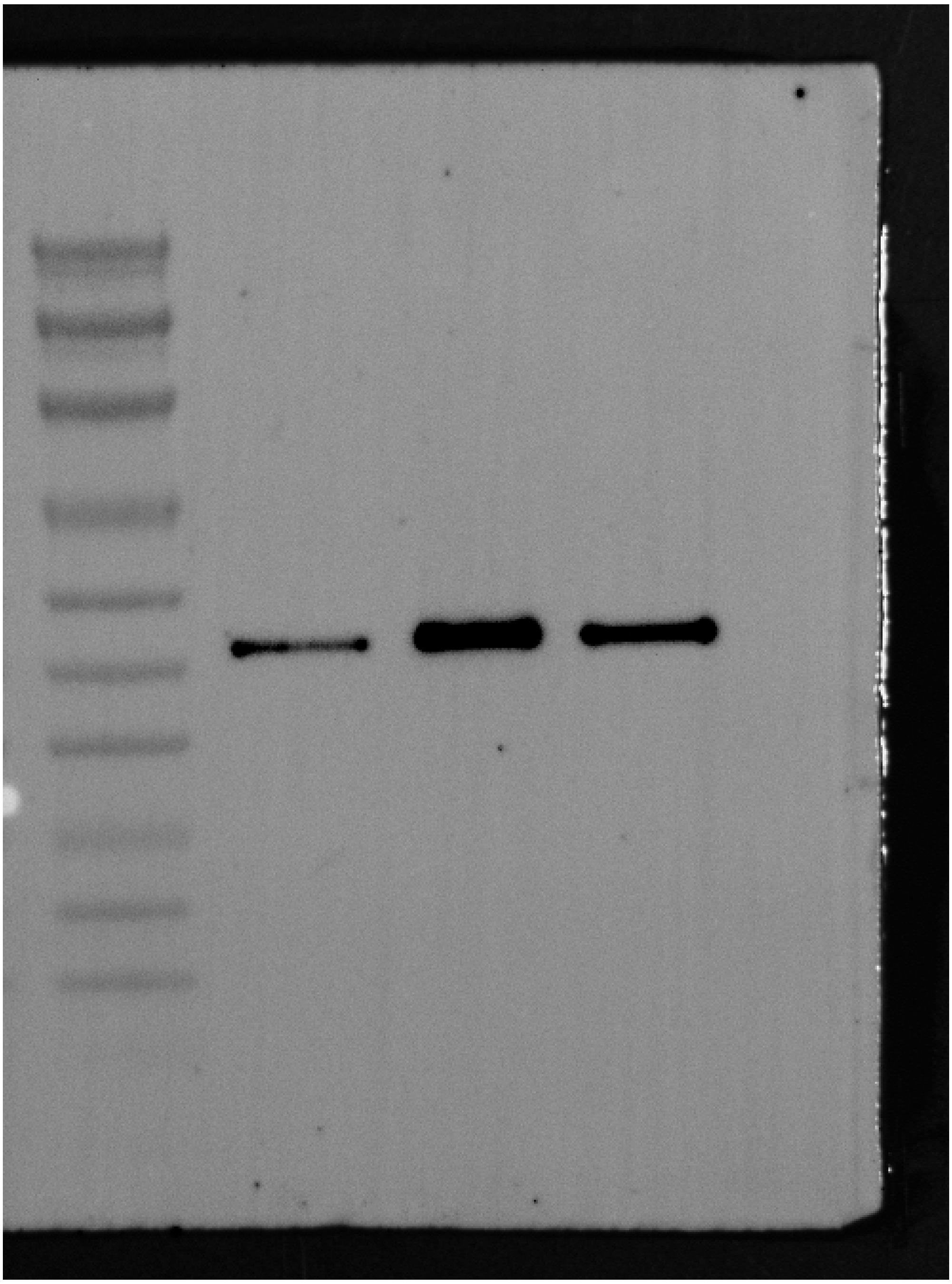

ADIPOQ-1

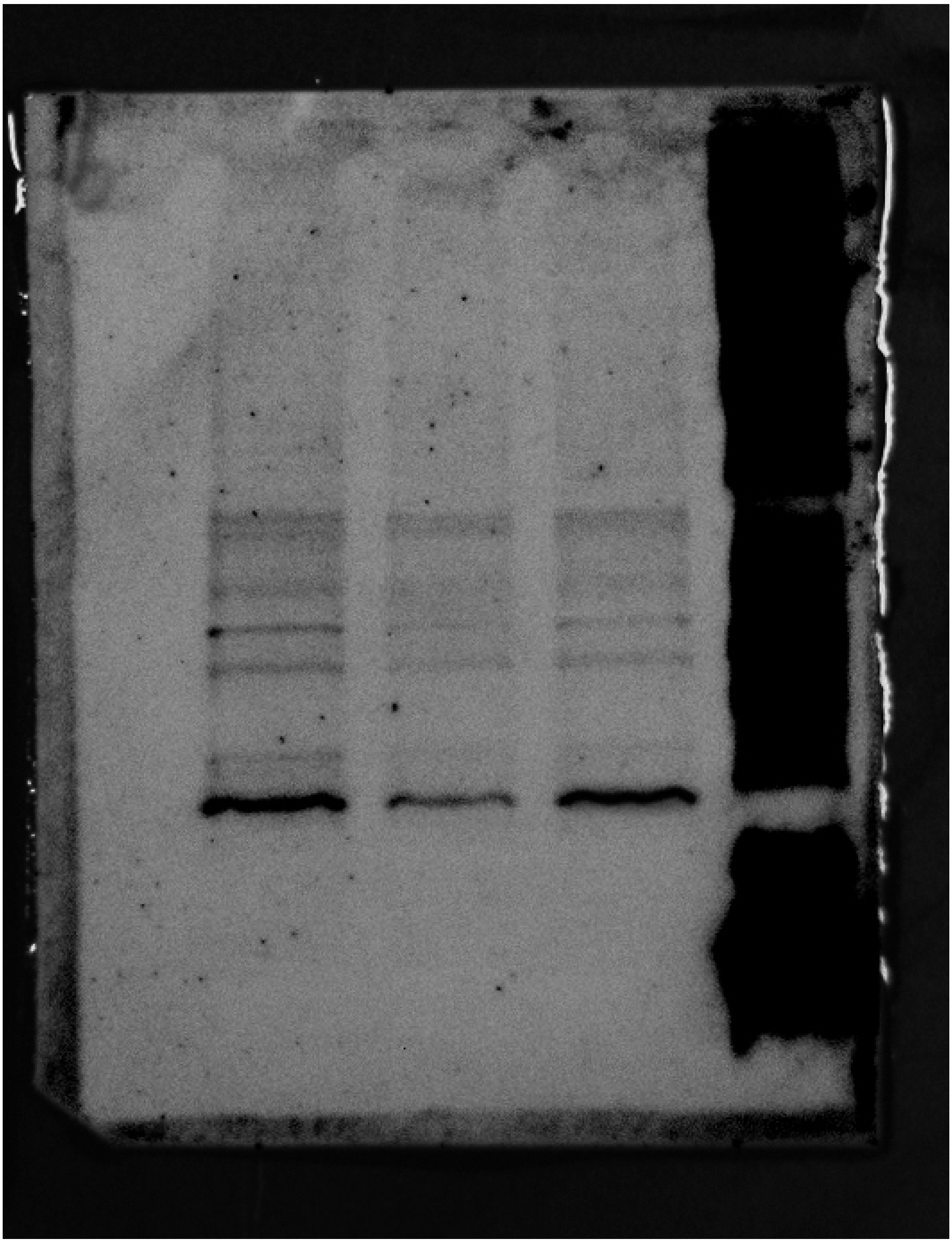

ADIPOQ-2

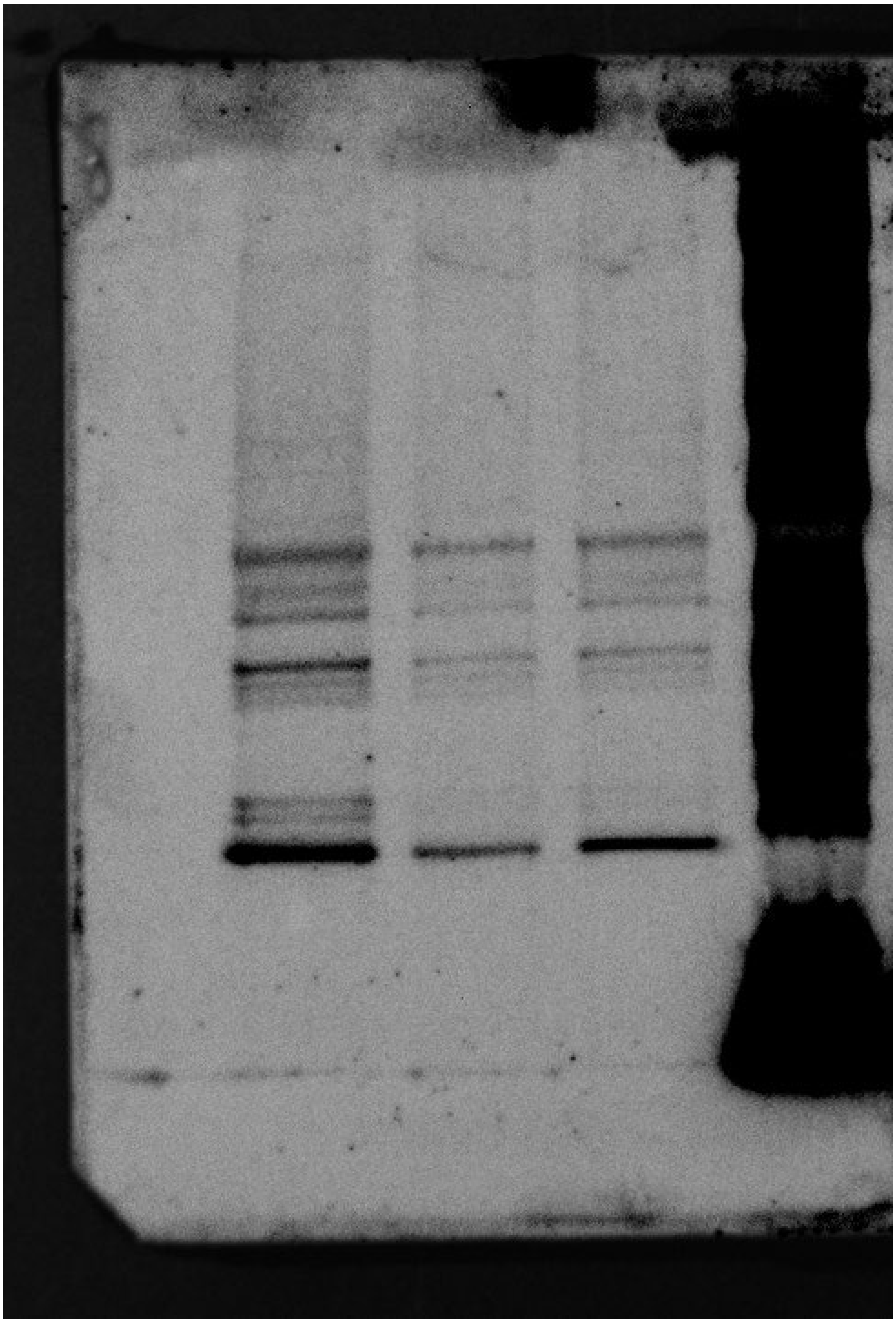

ADIPOQ-3

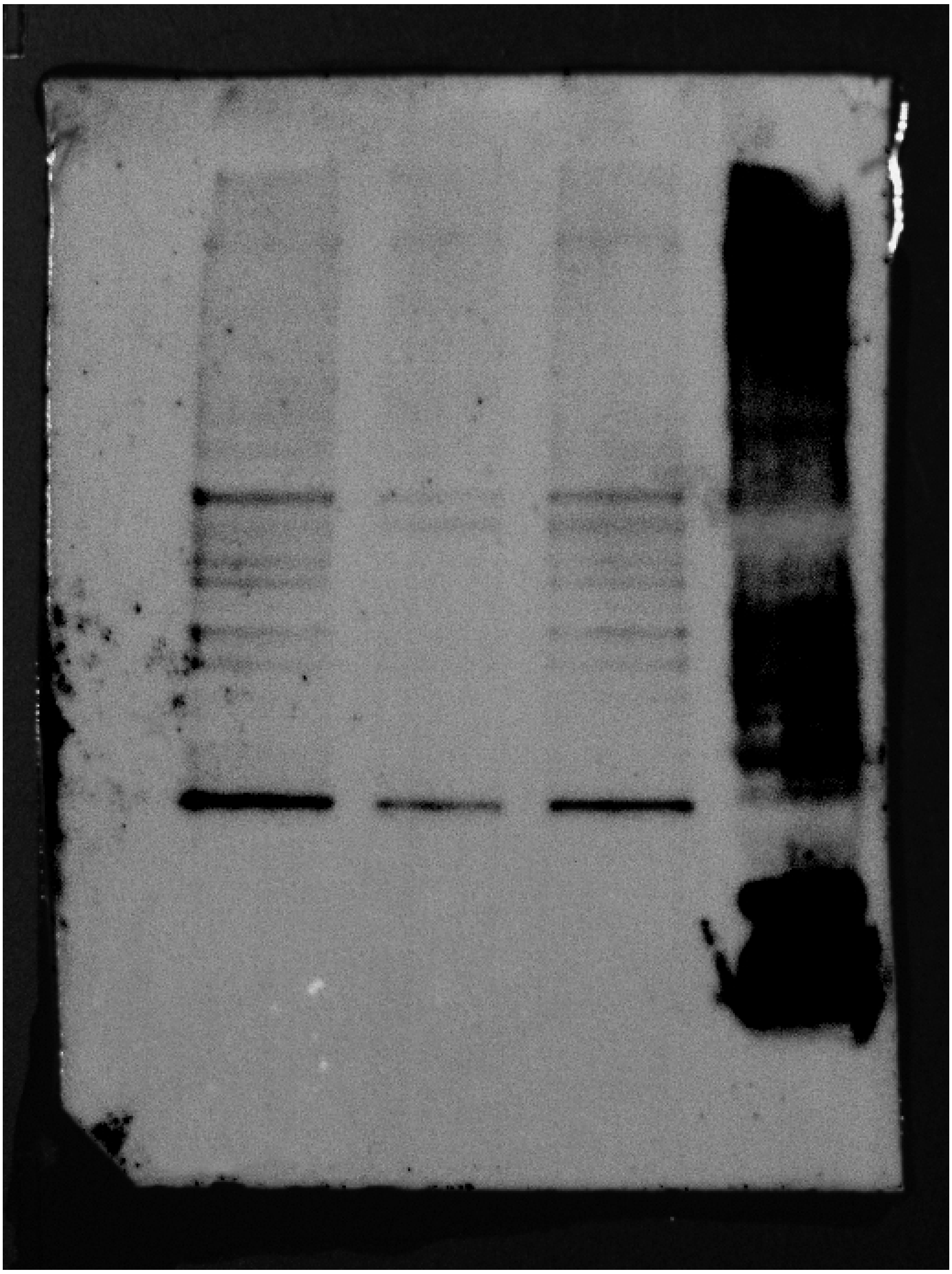

GAPDH-1

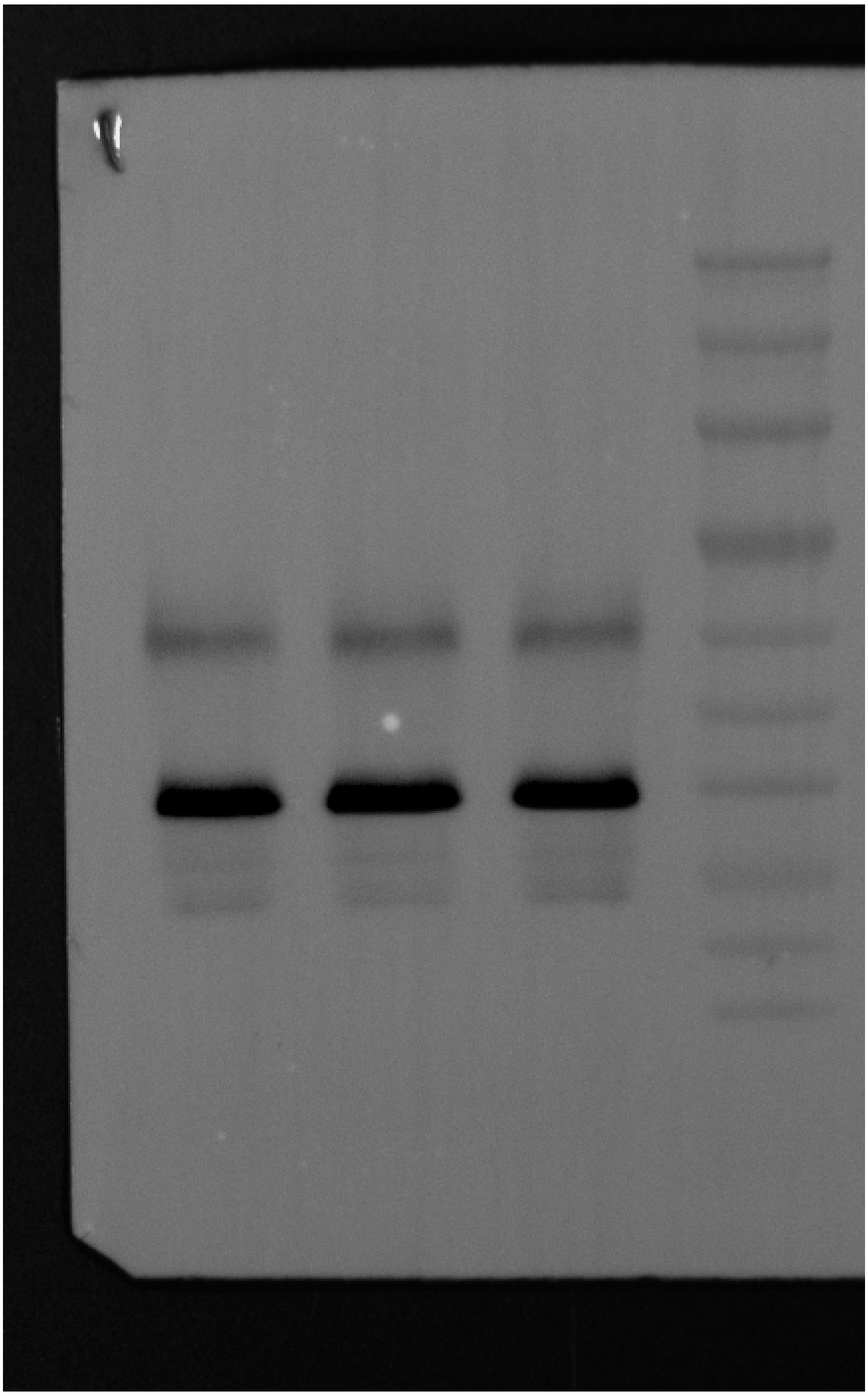

GAPDH-2

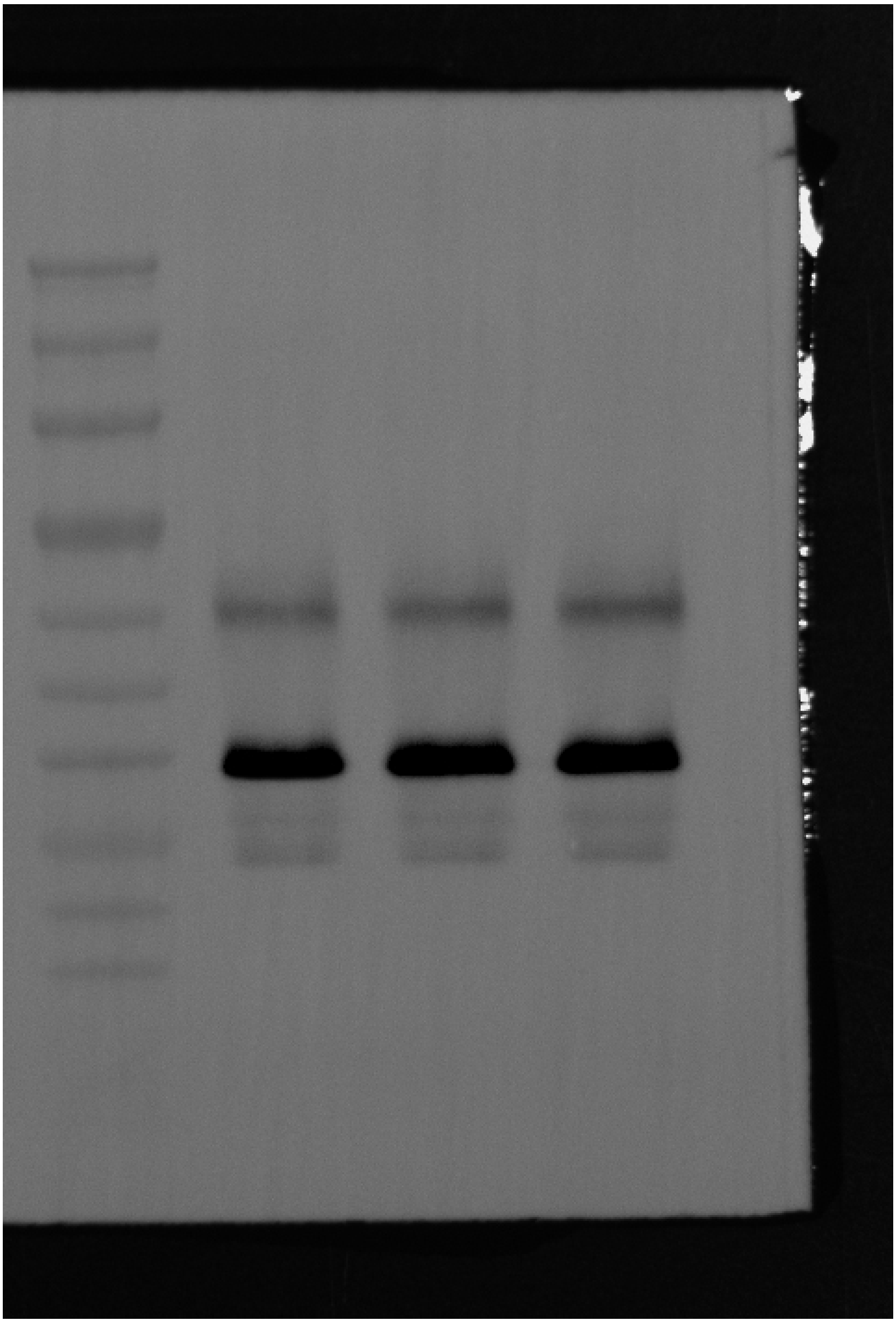

GAPDH-3

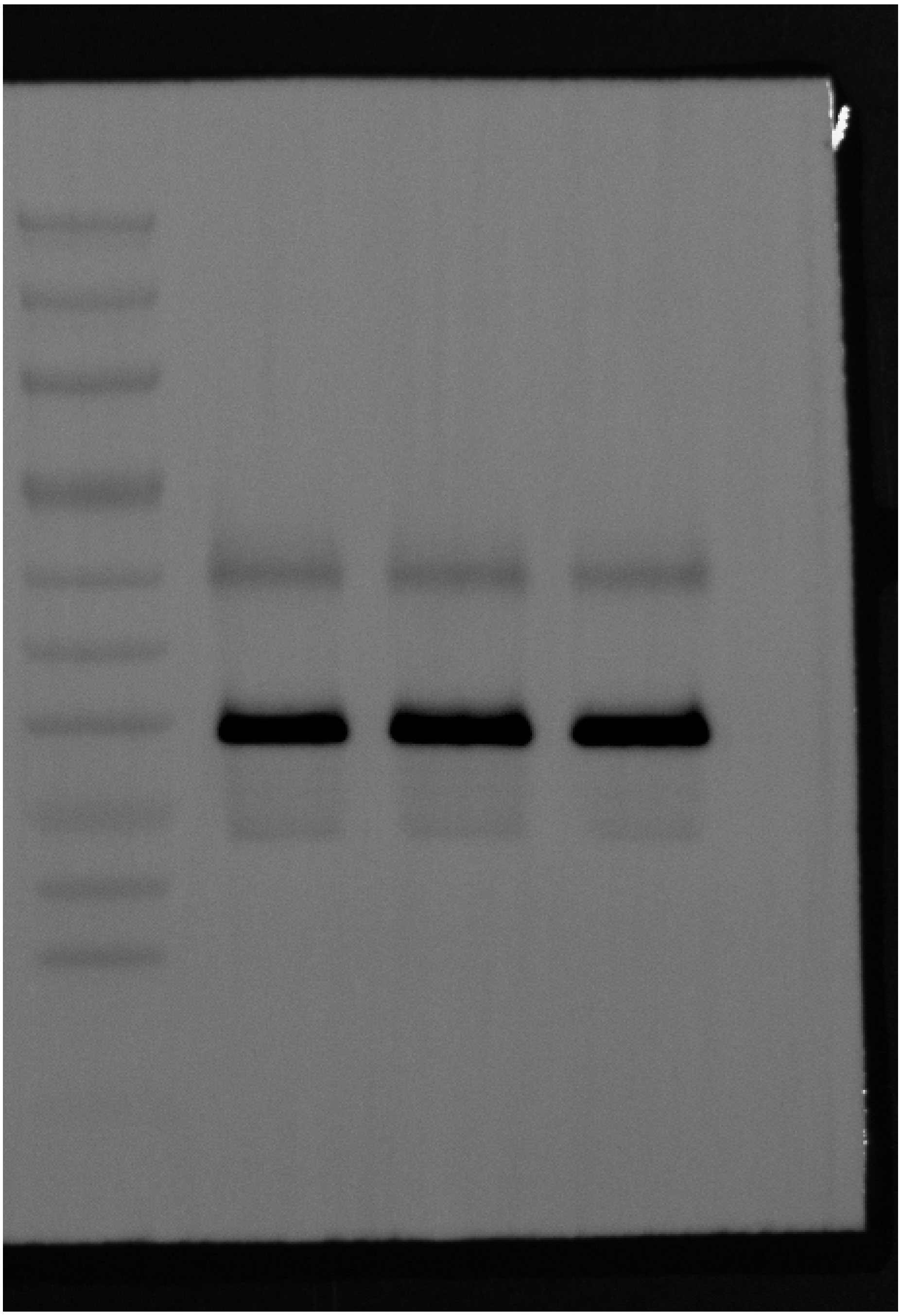

GAPDH-4

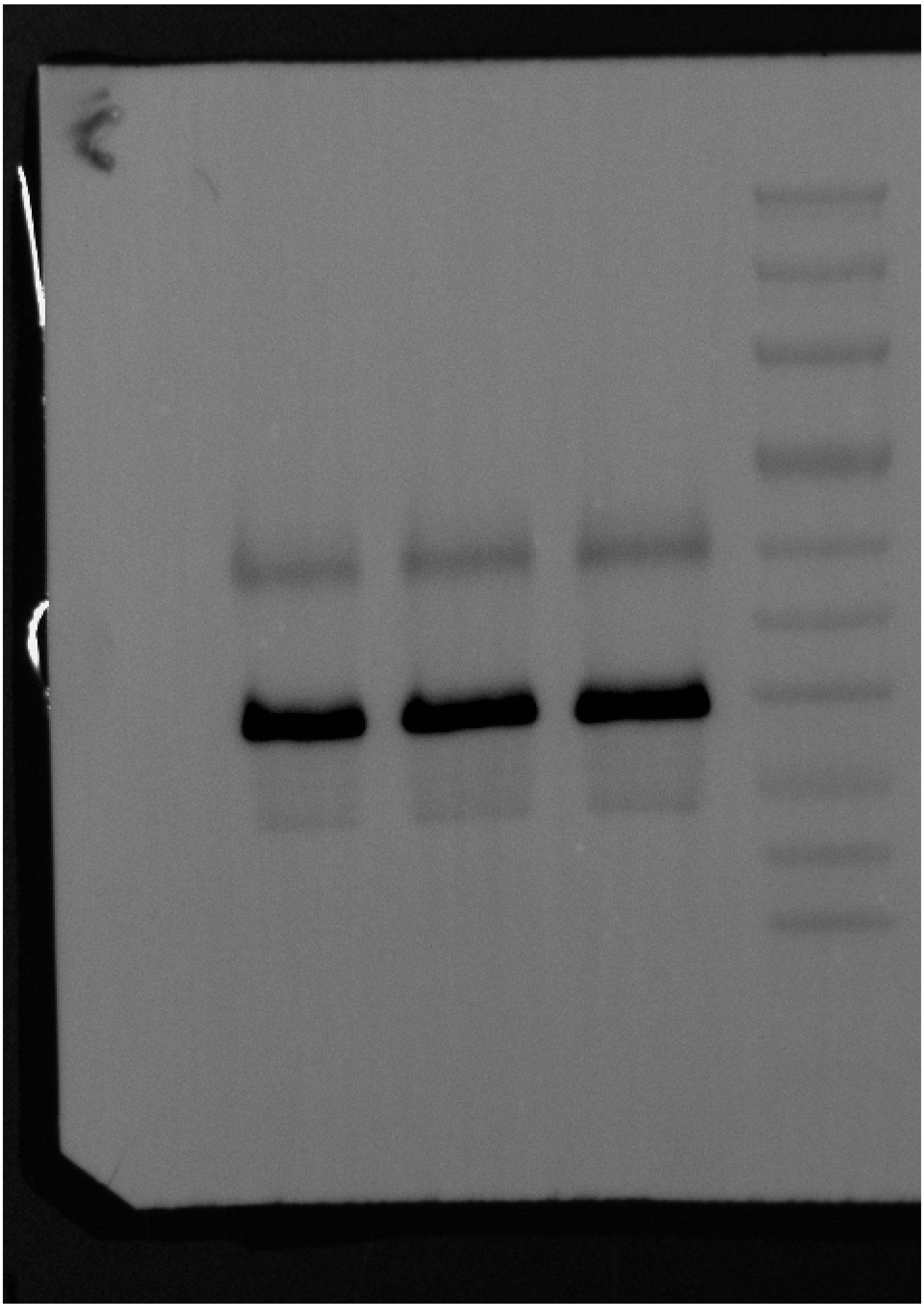

GAPDH-5

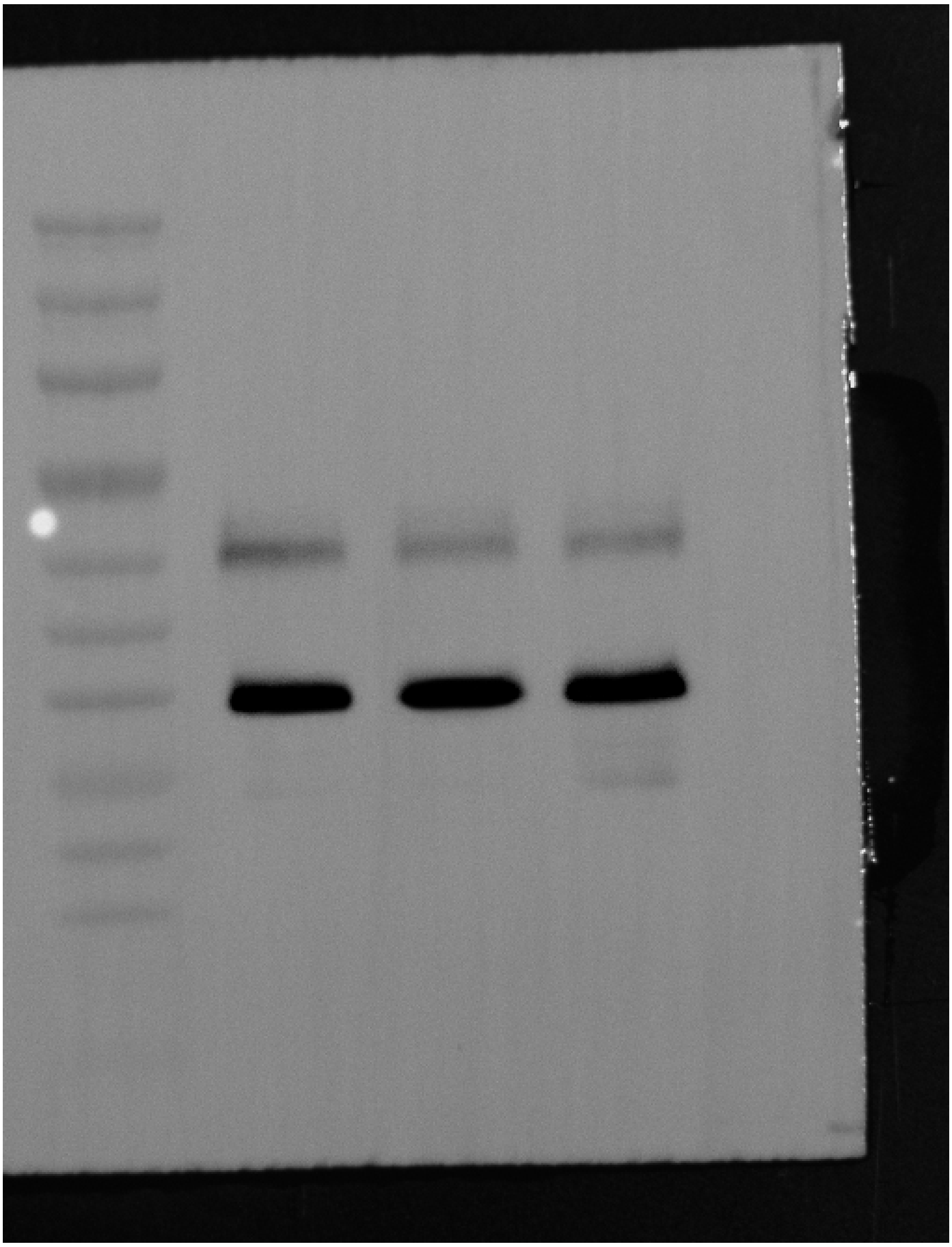

GAPDH-6

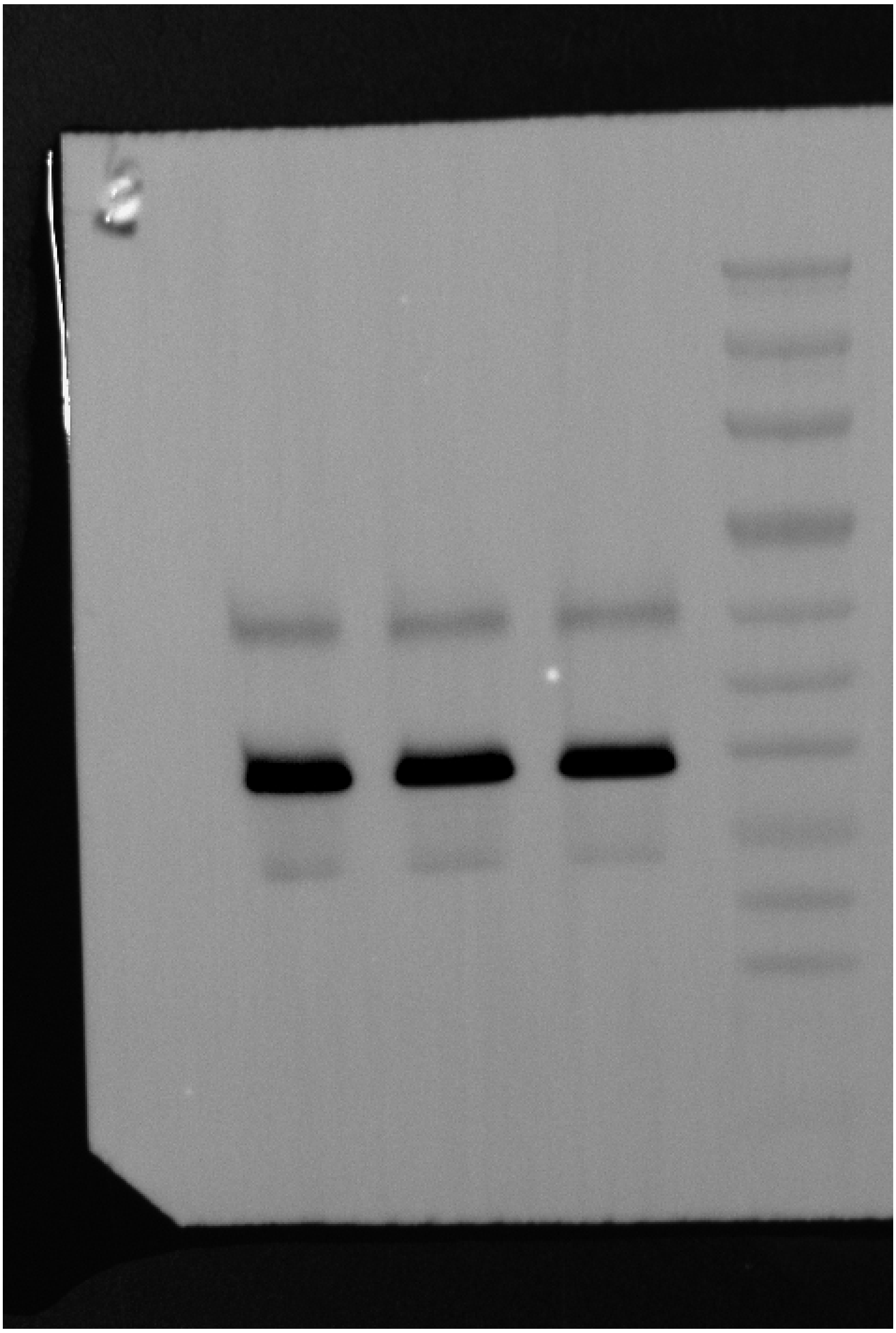

TLR4-1

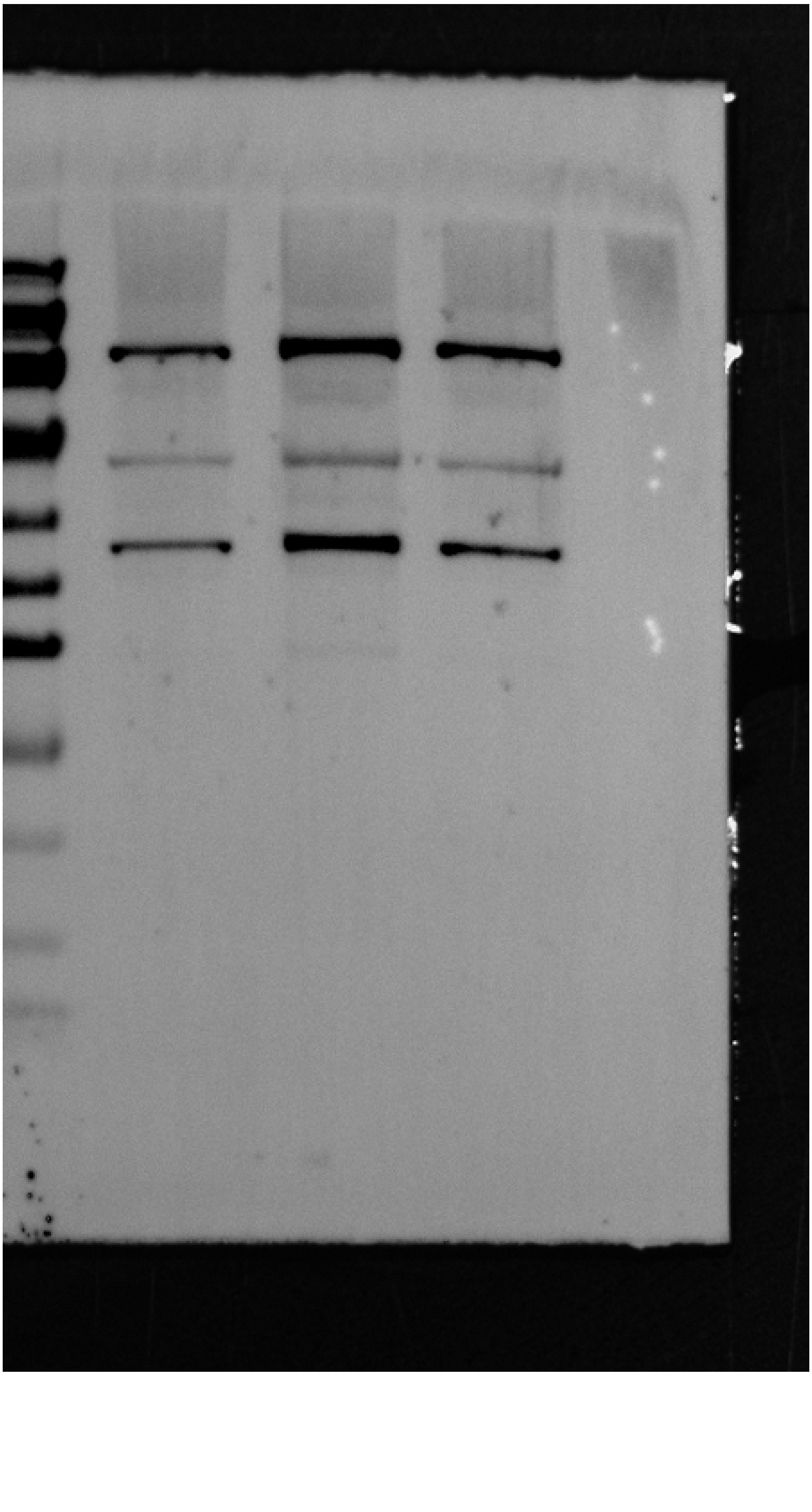

TLR4-2

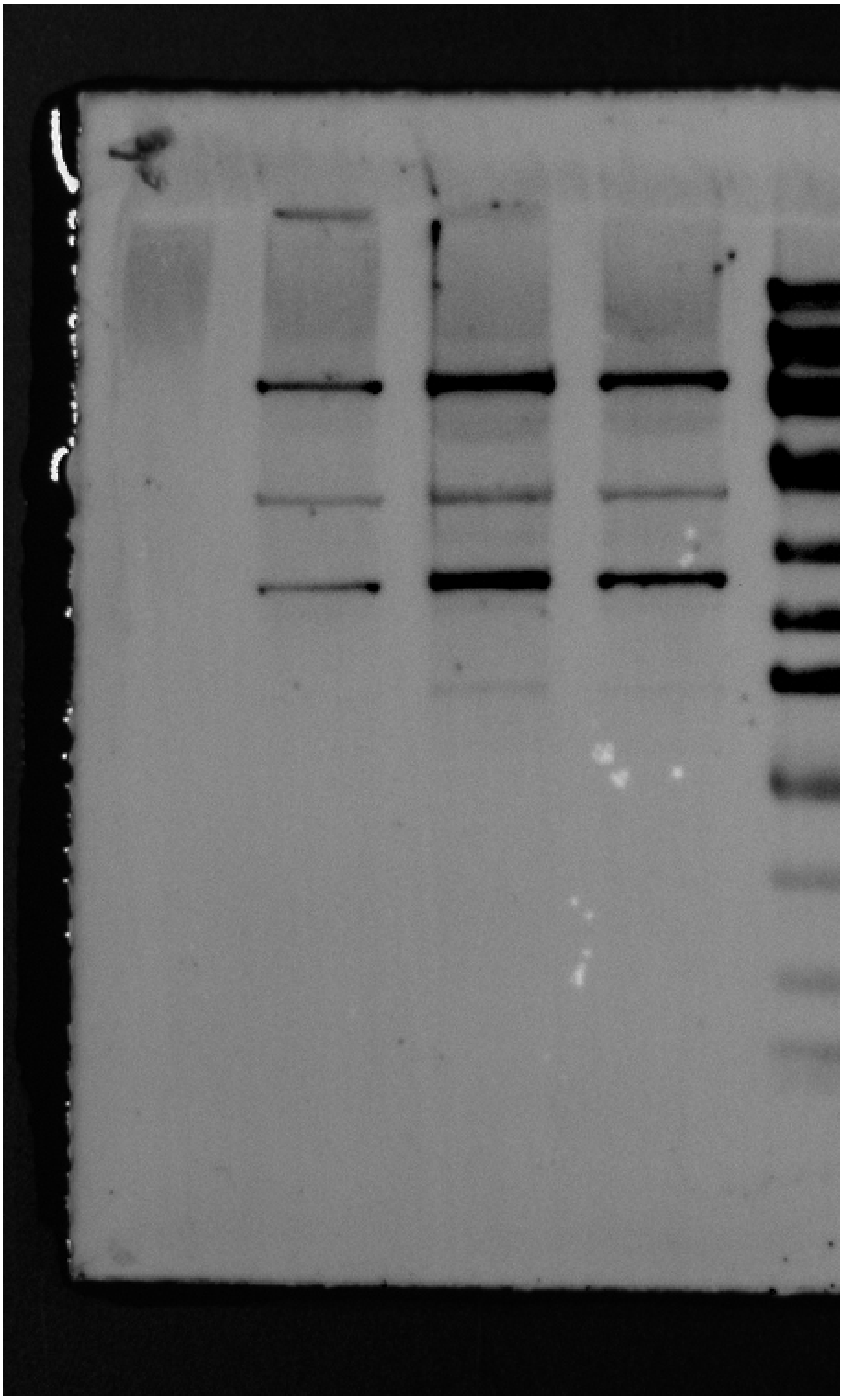

TLR4-3

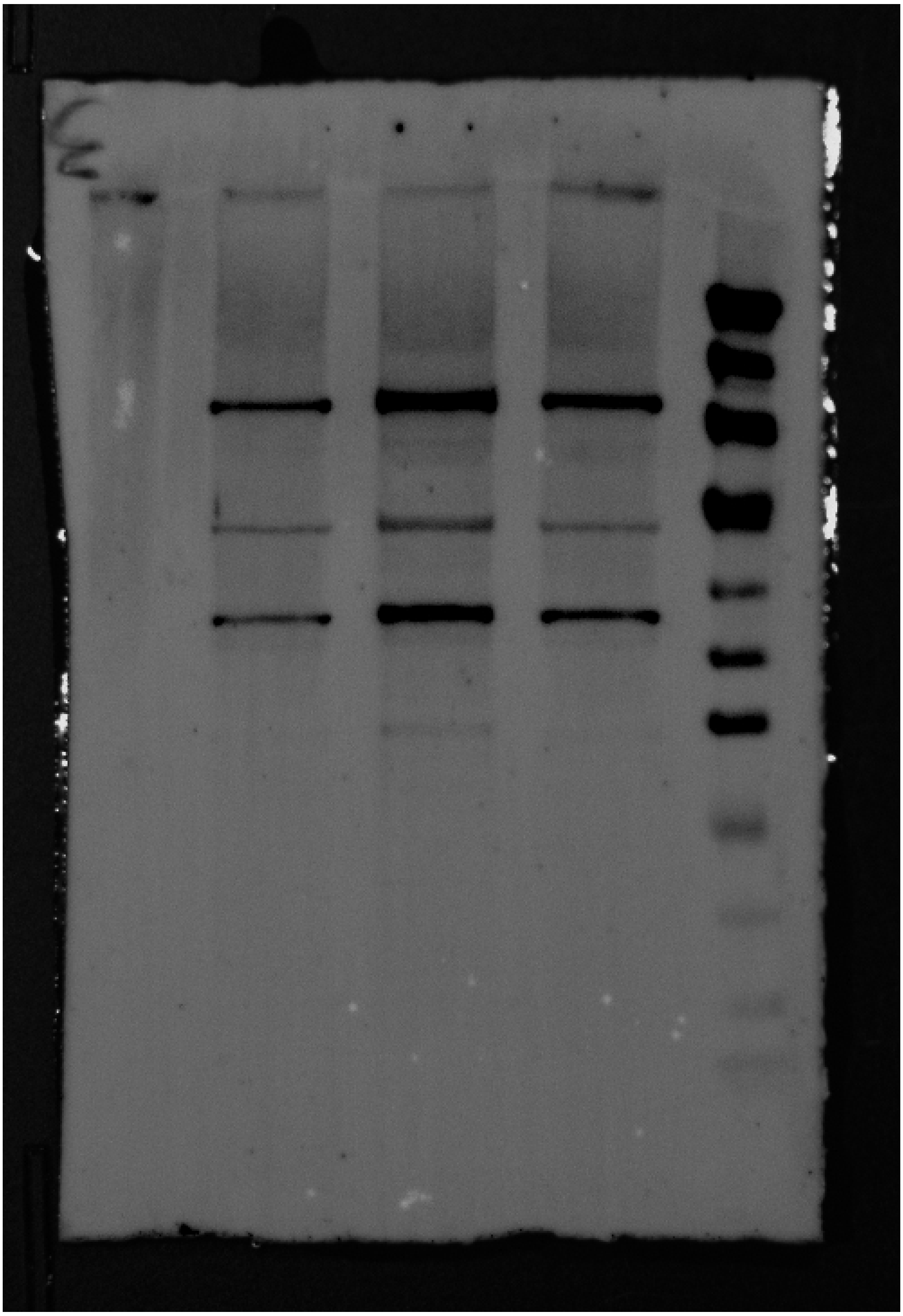

IKKβ-1

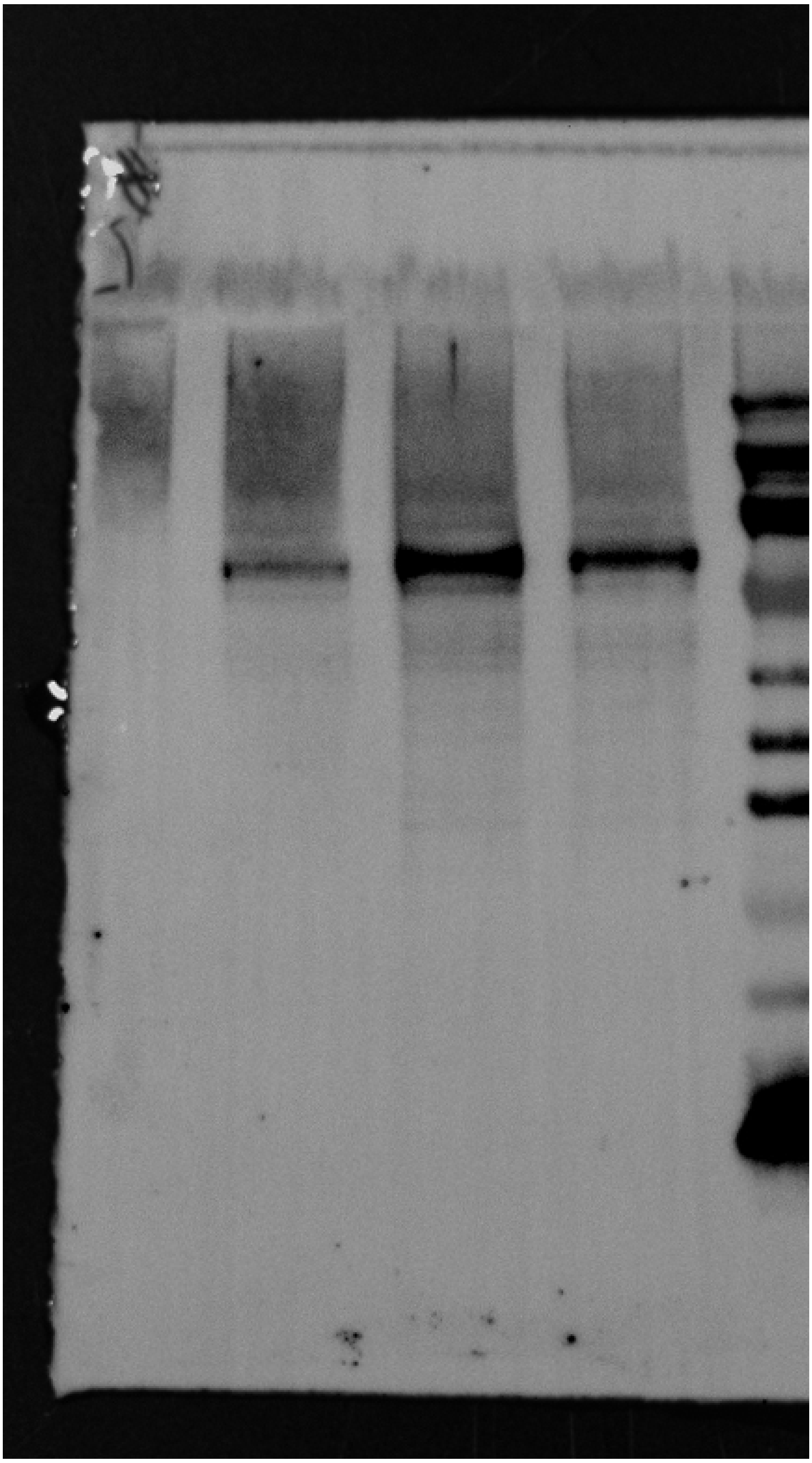

IKKβ-2

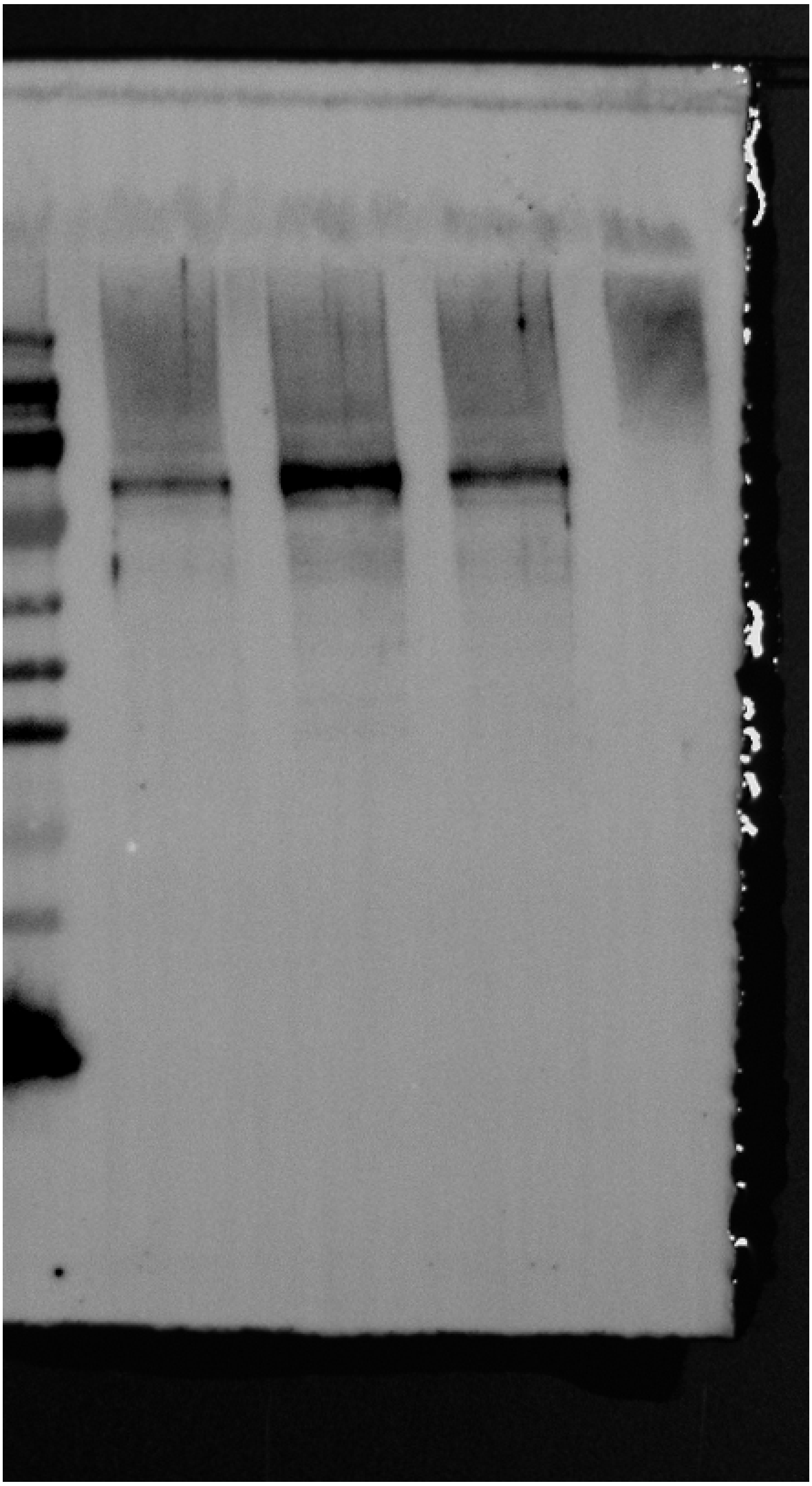

IKKβ-3

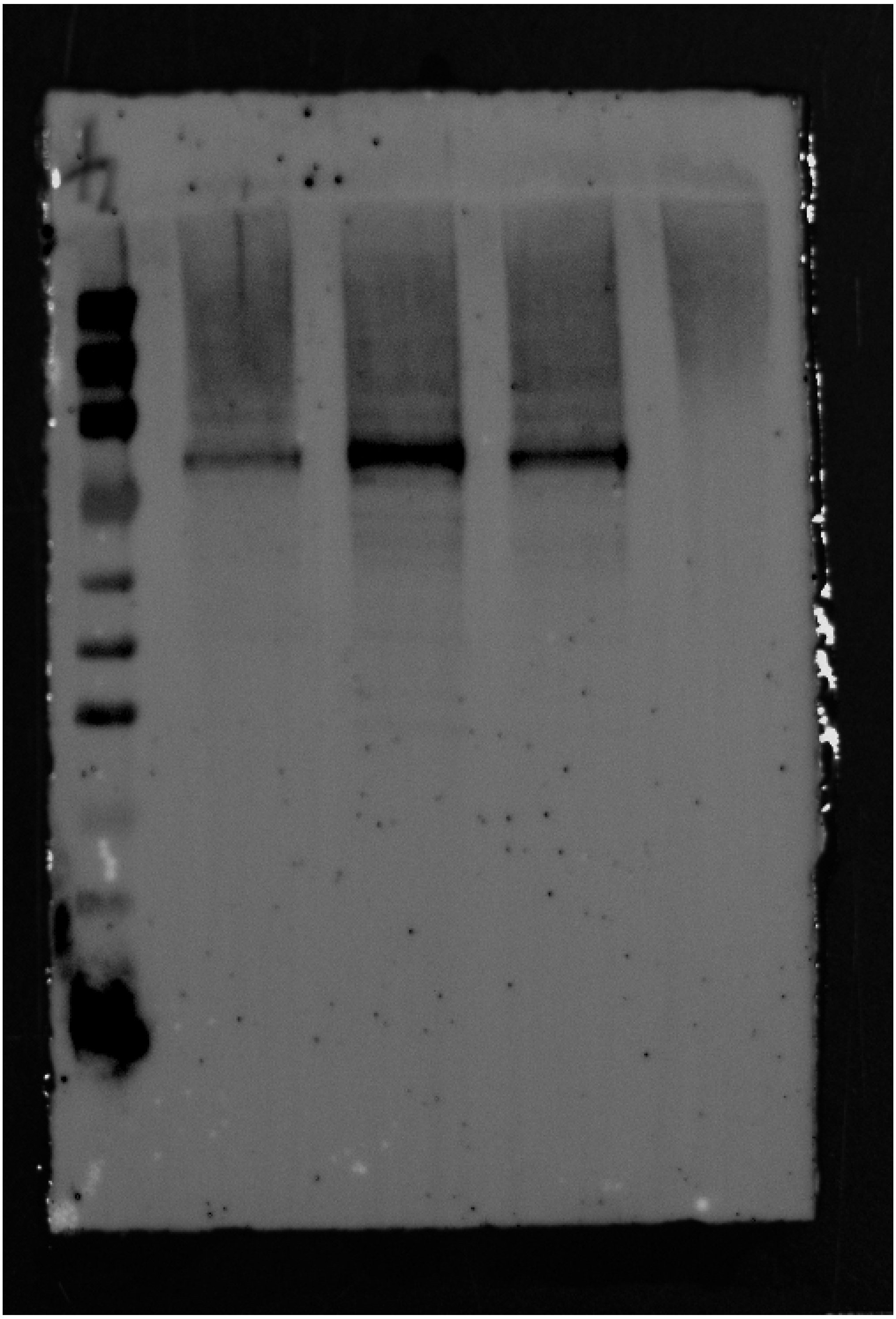

p-P65-1

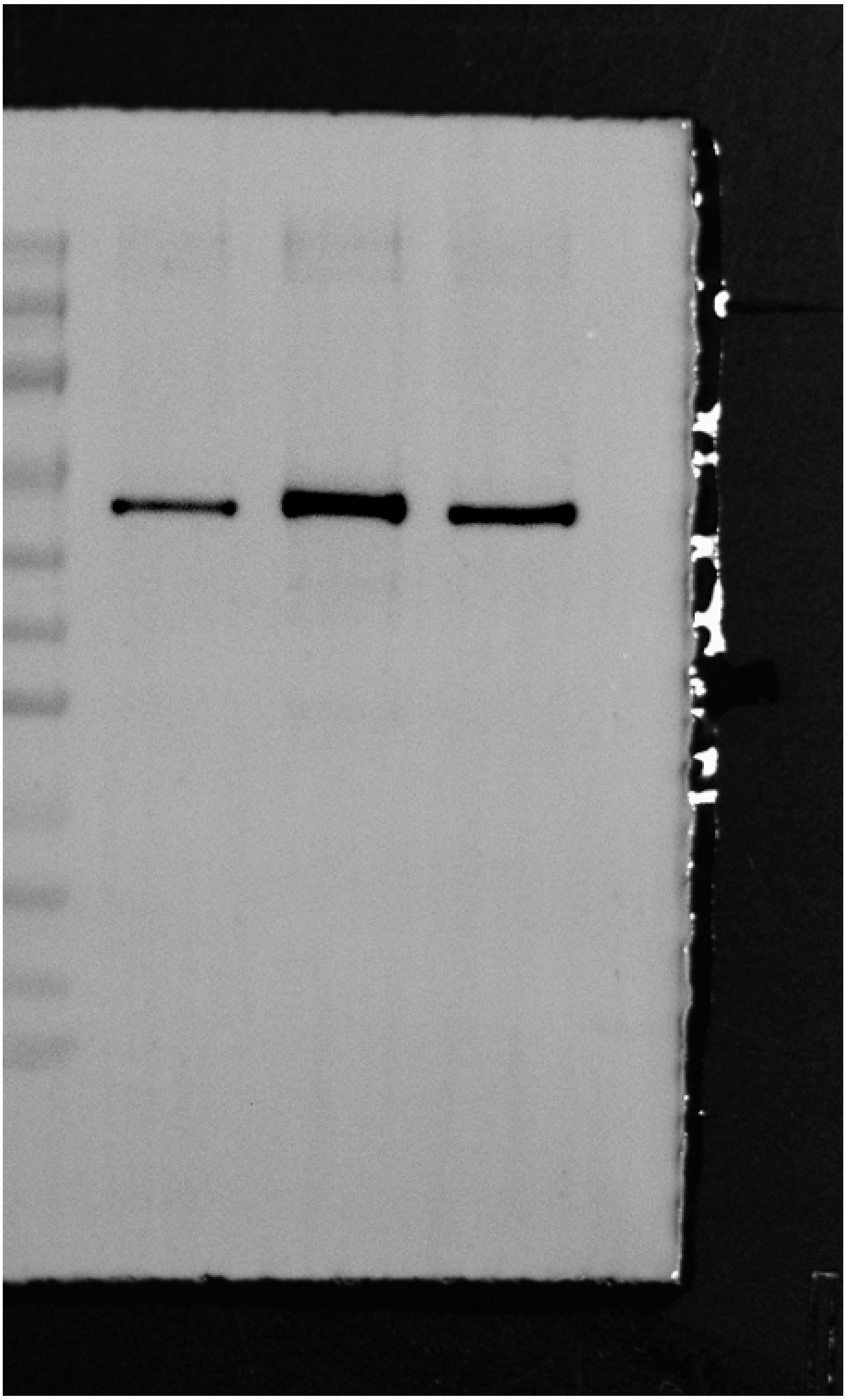

p-P65-2

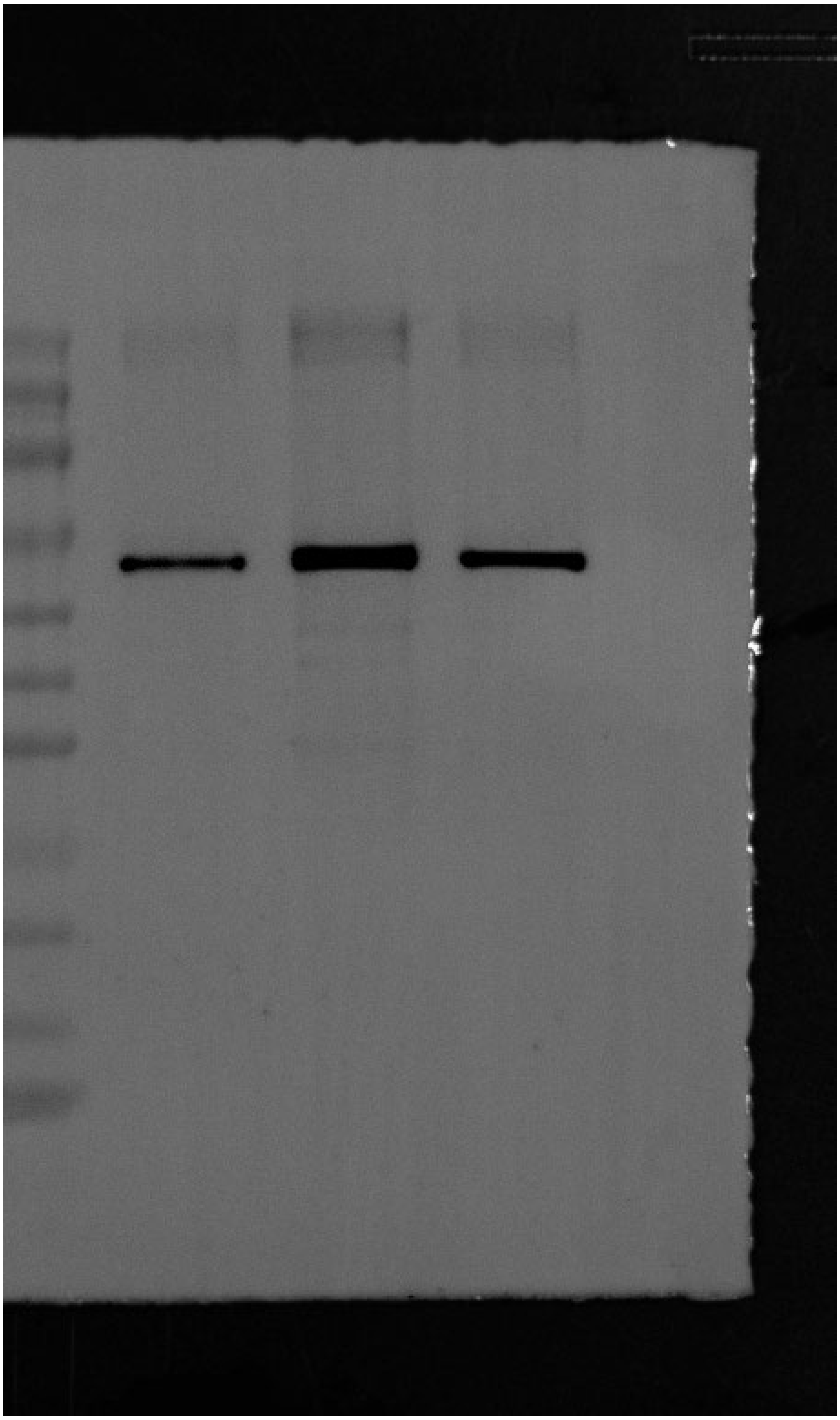

p-P65-3

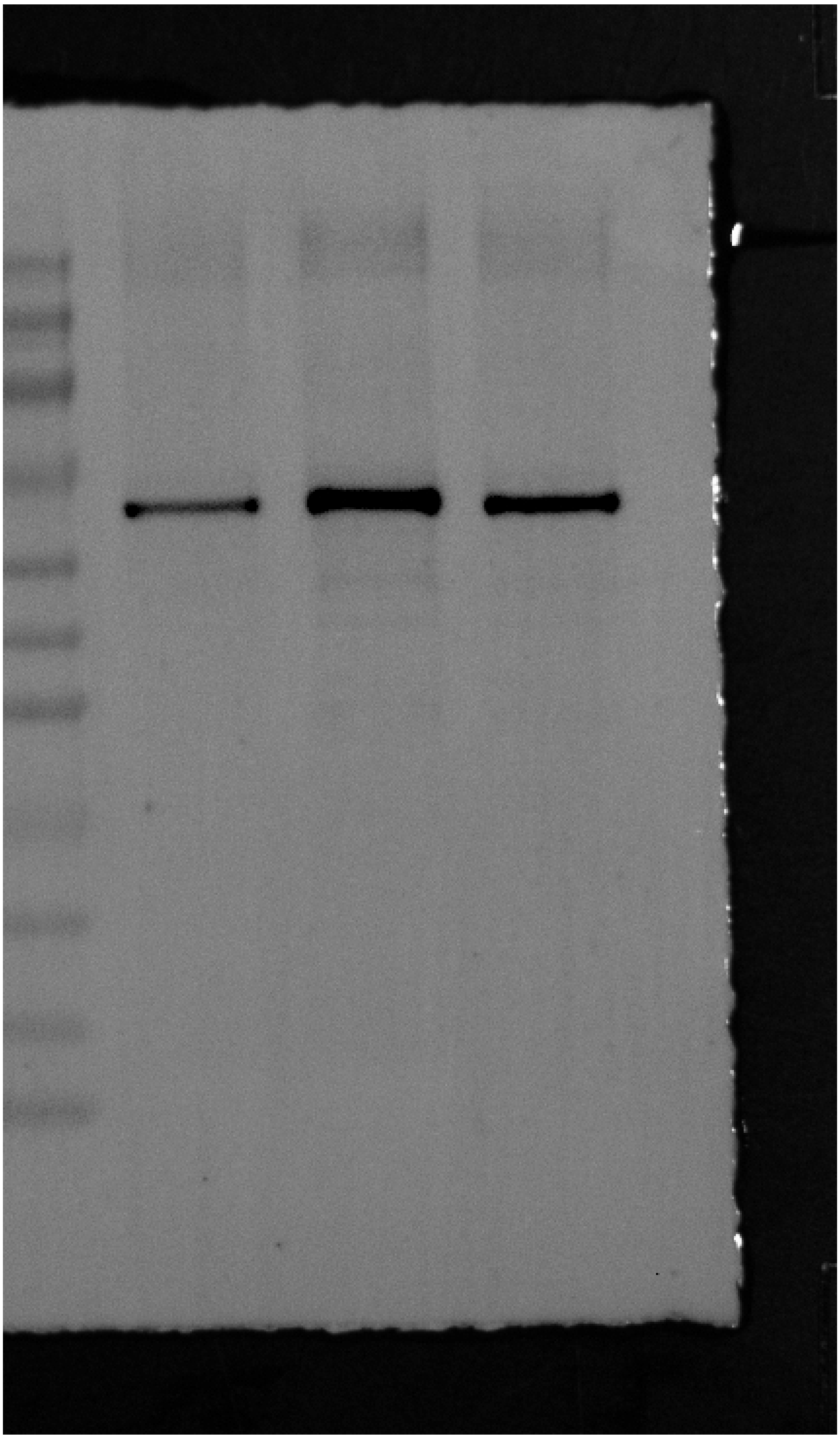

GAPDH-1

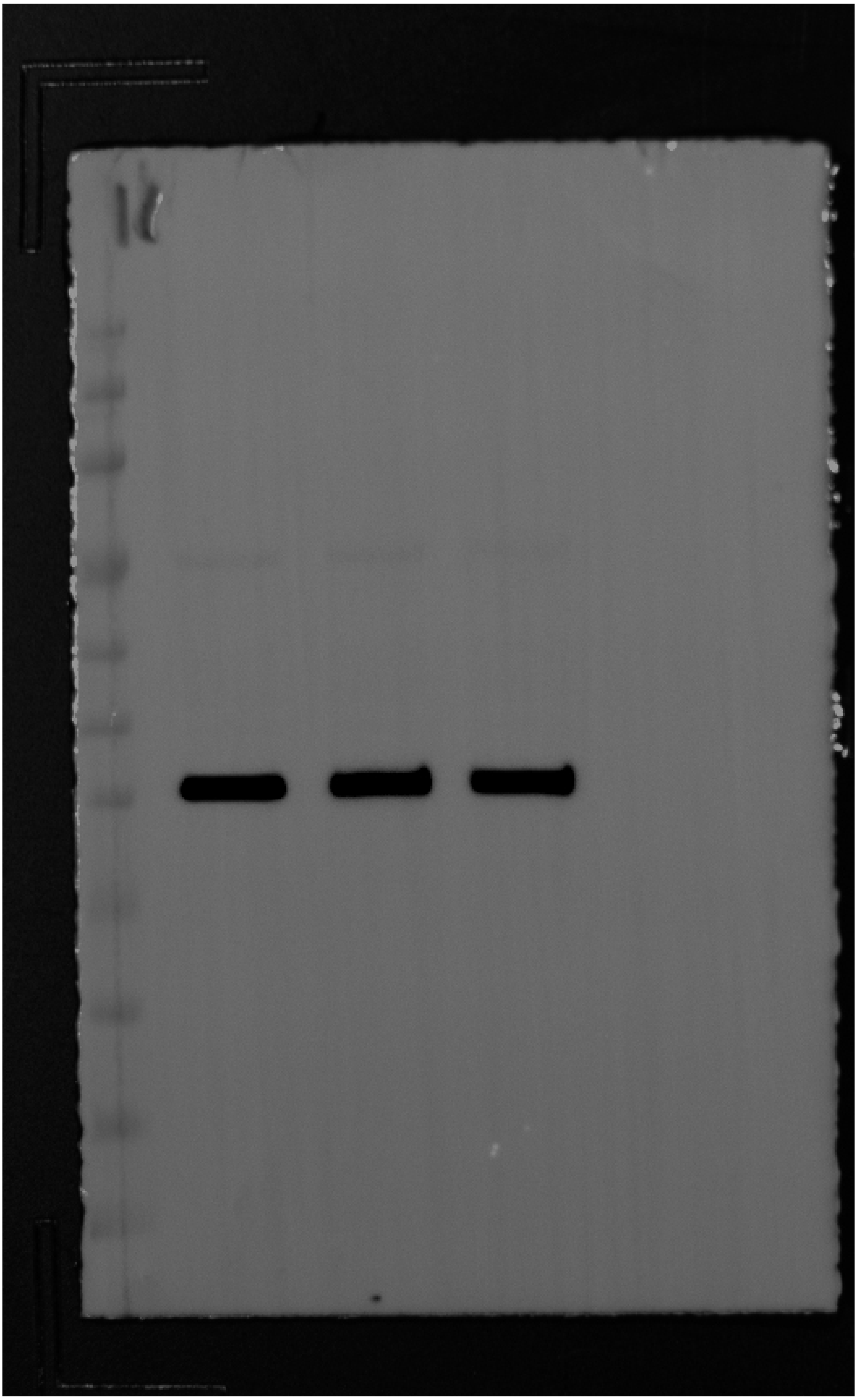

GAPDH-2

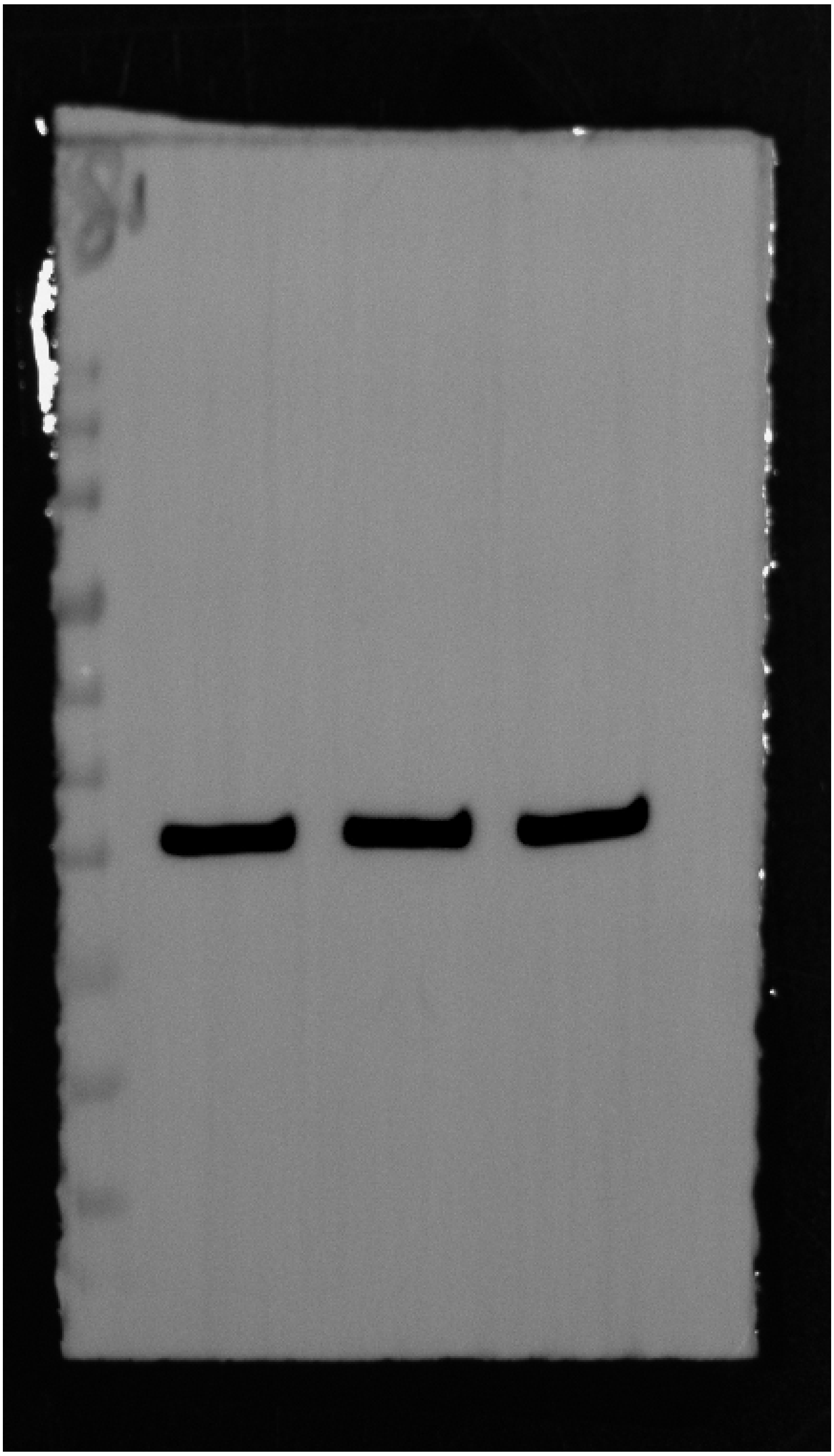

GAPDH-3

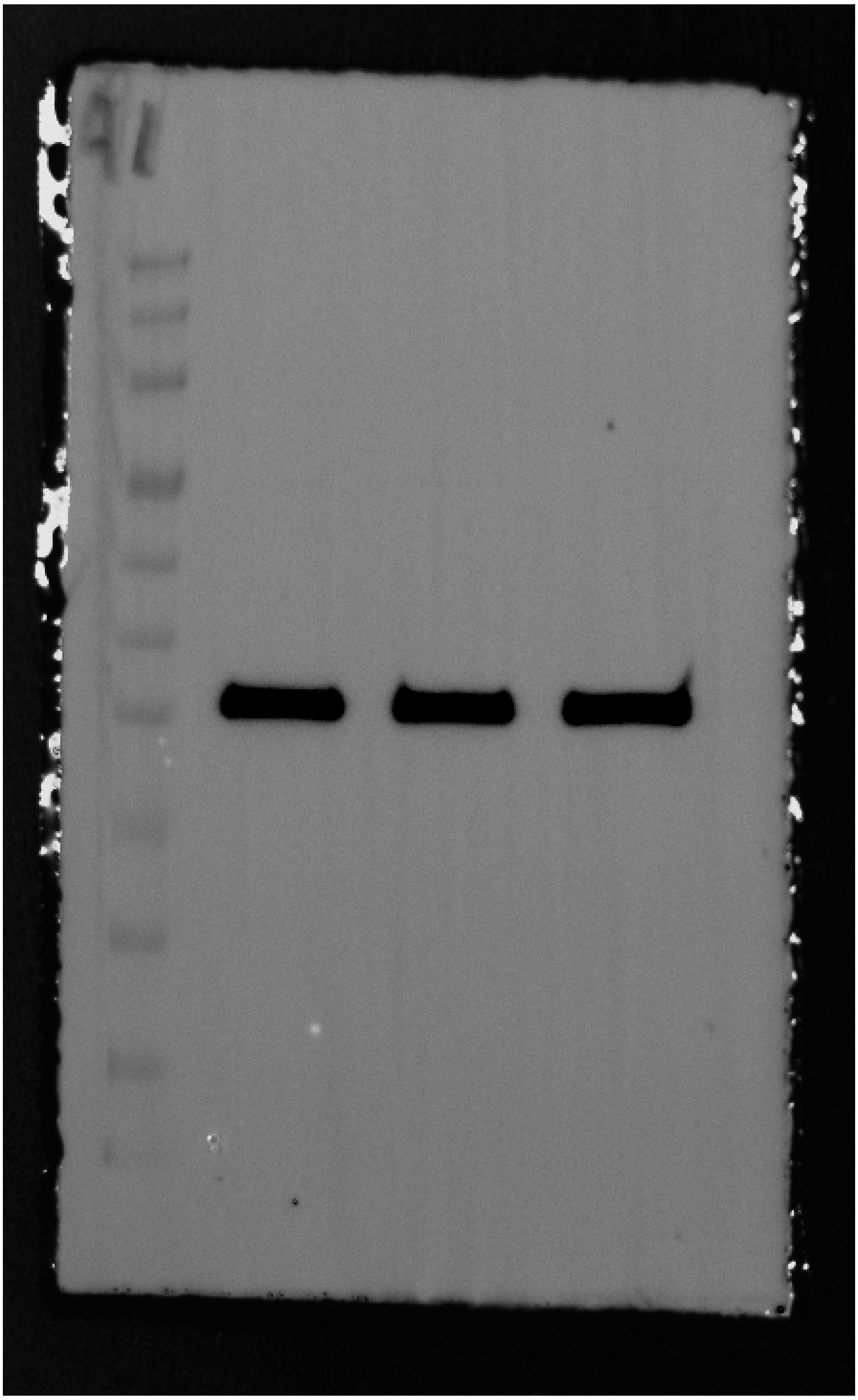

Supplement: Supplementary file 1 [file foods-15-01396-s001.zip › Uncropped, unprocessed and full gel and blot2.pdf]
