# Supplementary figures and images for: Chemical Composition Analysis of Highland Barley (Hordeum vulgare L.) with Different Modification Methods and Lipid Metabolism Mechanism Analysis of Highland Barley with Microwave Fluidization Modification
Source: Foods. 2026 Apr 17;15(8):1396. doi: 10.3390/foods15081396 (PMC13114515; doi:10.3390/foods15081396)

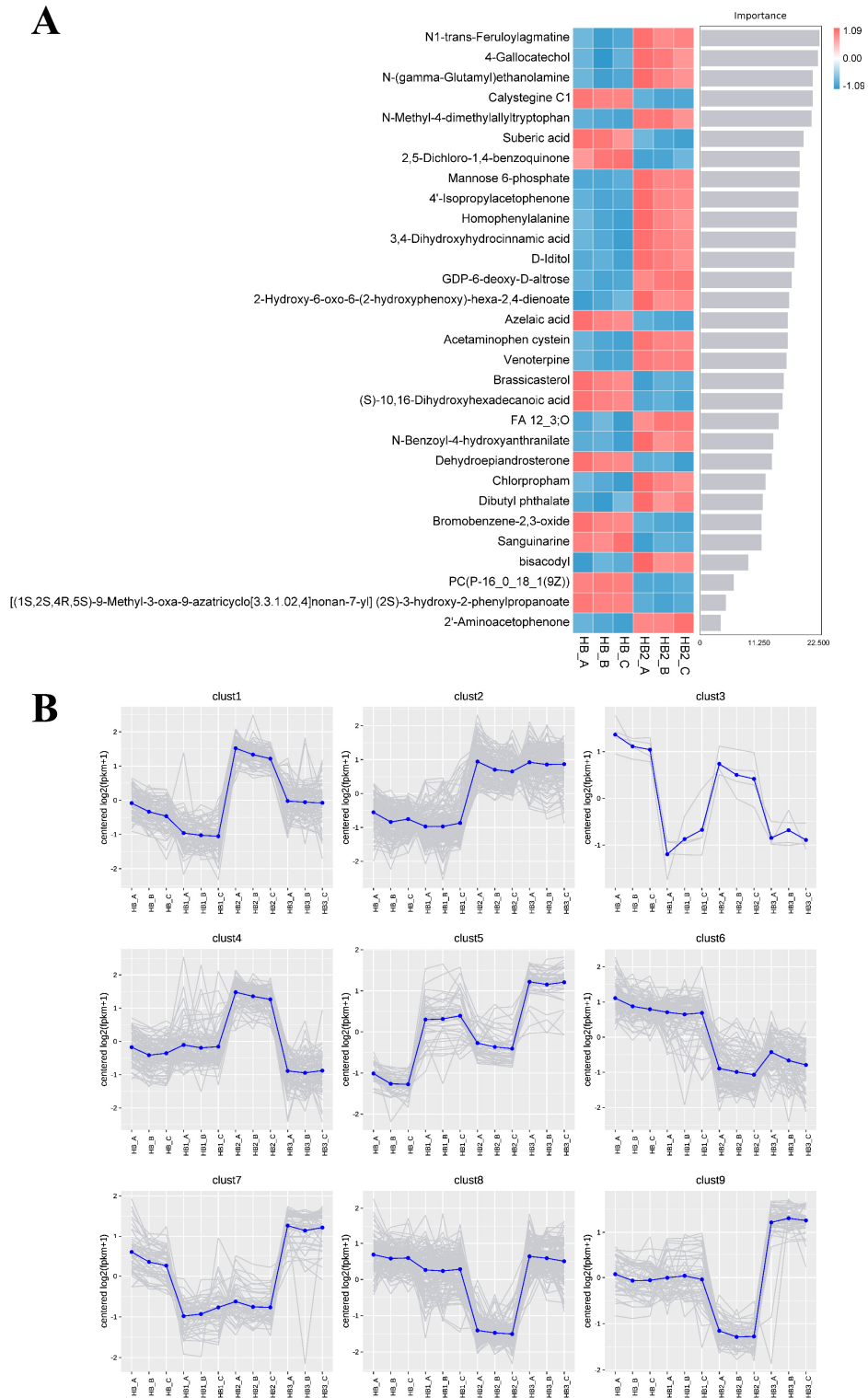

**Figure S10** (A) Random Forest plot of HB VS HB-2; (B) Differential metabolites trend analysis.

Supplement: Supplementary file 1 [file foods-15-01396-s001.zip › Figure S10.pdf]

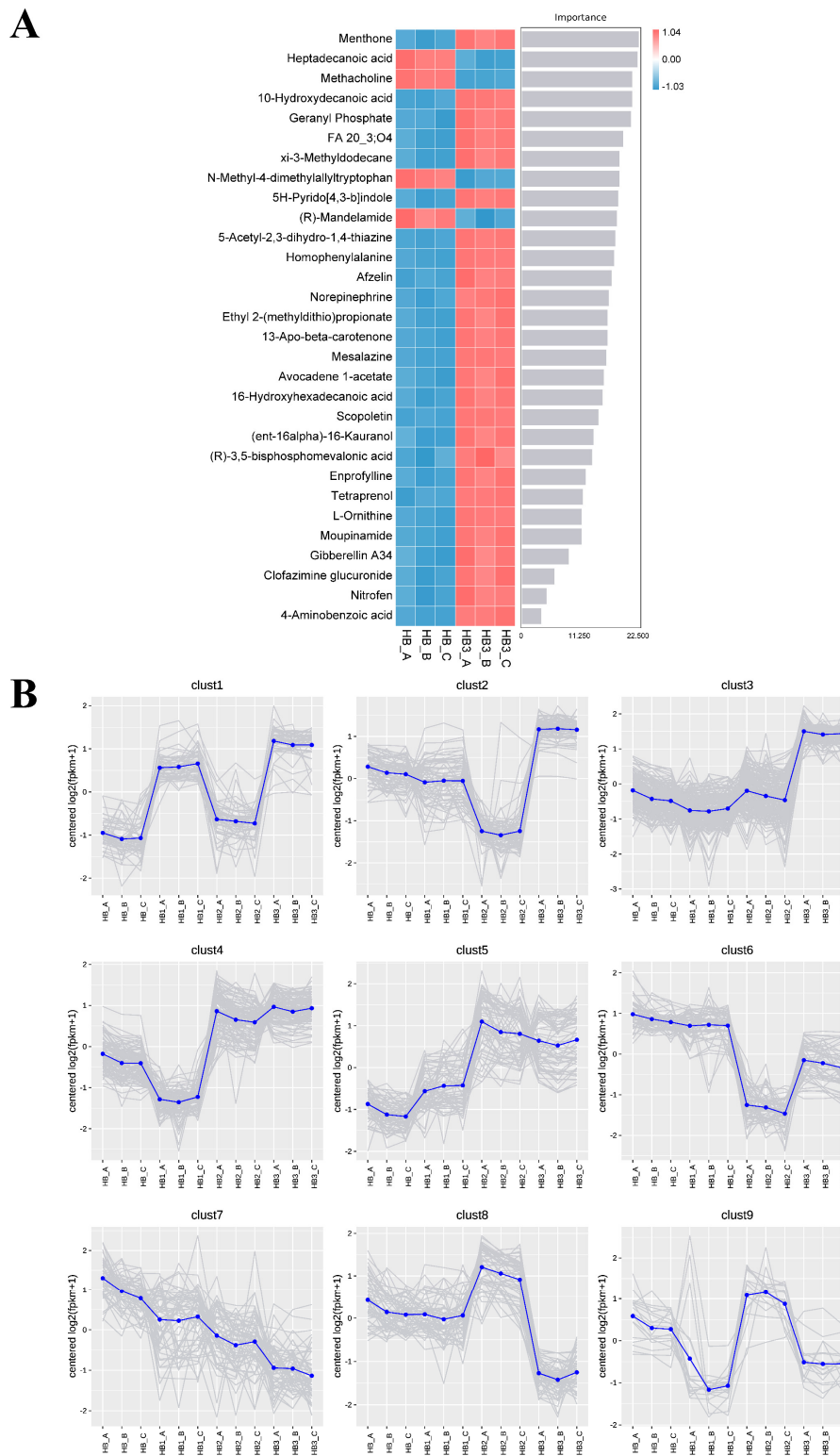

**Figure S11** (A) Random Forest plot of HB VS HB-3; (B) Differential metabolites trend analysis.

Supplement: Supplementary file 1 [file foods-15-01396-s001.zip › Figure S11.pdf]

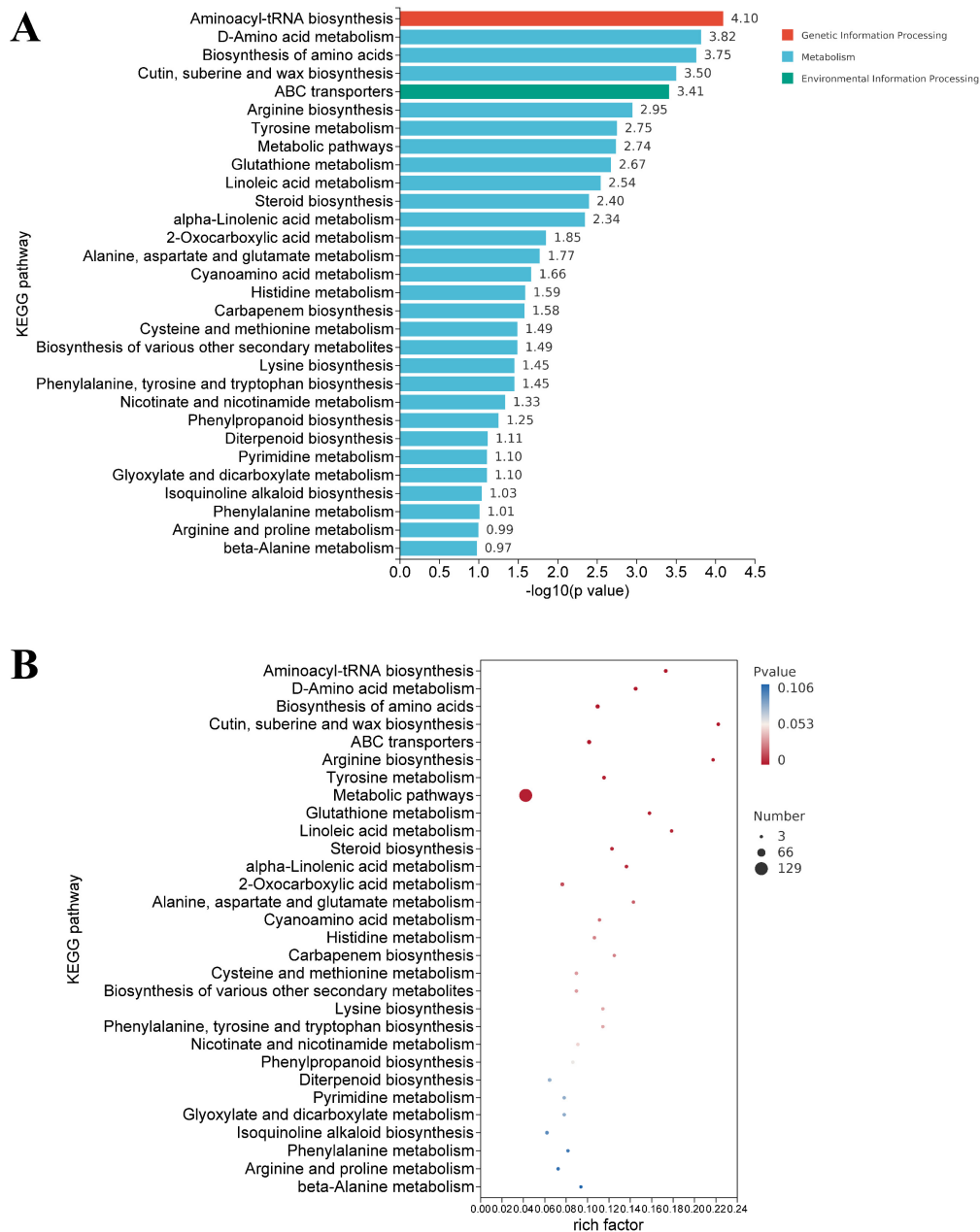

**Figure S12** (A) KEGG enrichment bar plot of HB VS HB-1; (B) KEGG enrichment factor plot of HB VS HB-1.

Supplement: Supplementary file 1 [file foods-15-01396-s001.zip › Figure S12.pdf]

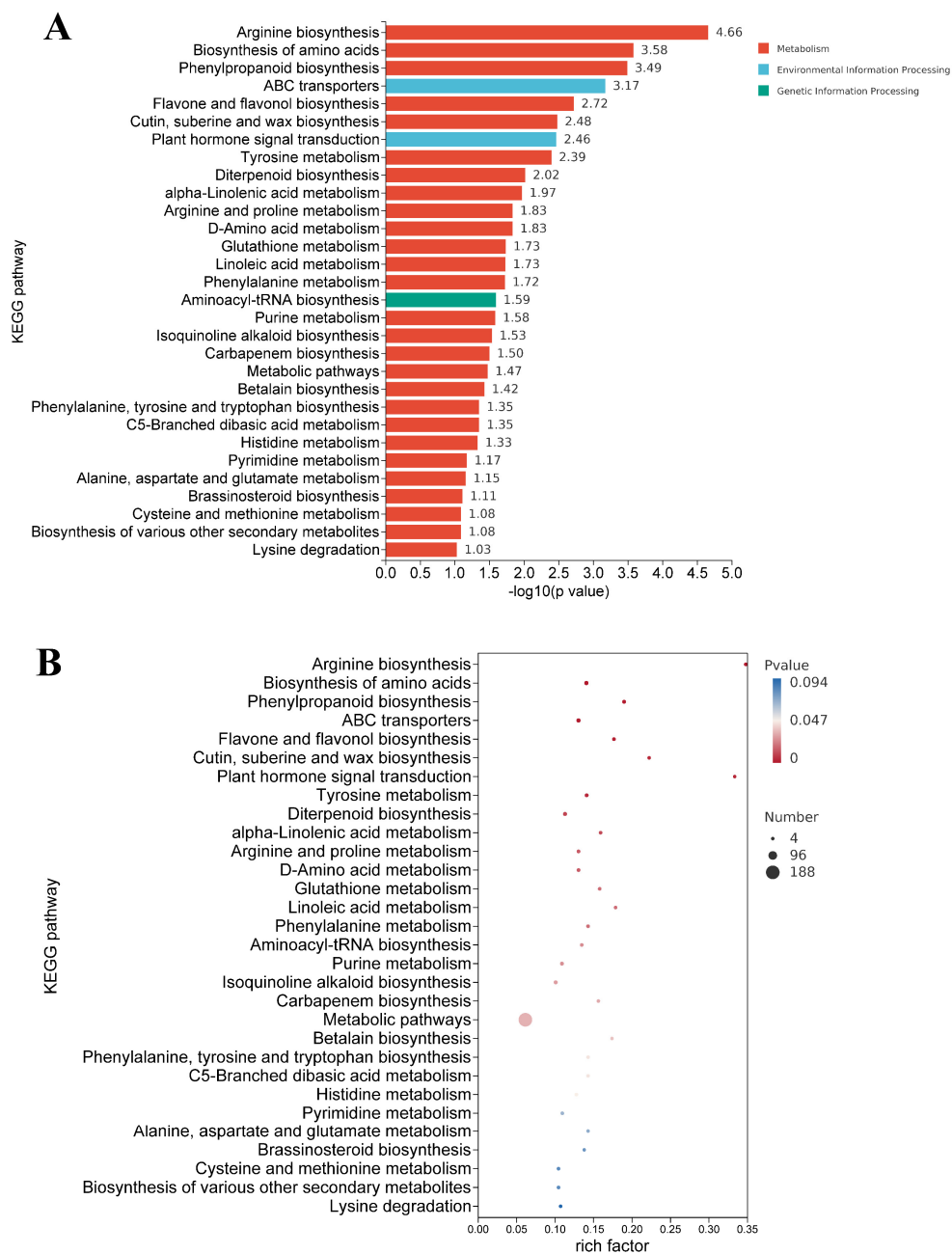

**Figure S13** (A) KEGG enrichment bar plot of HB VS HB-2; (B) KEGG enrichment factor plot of HB VS HB-2.

Supplement: Supplementary file 1 [file foods-15-01396-s001.zip › Figure S13.pdf]

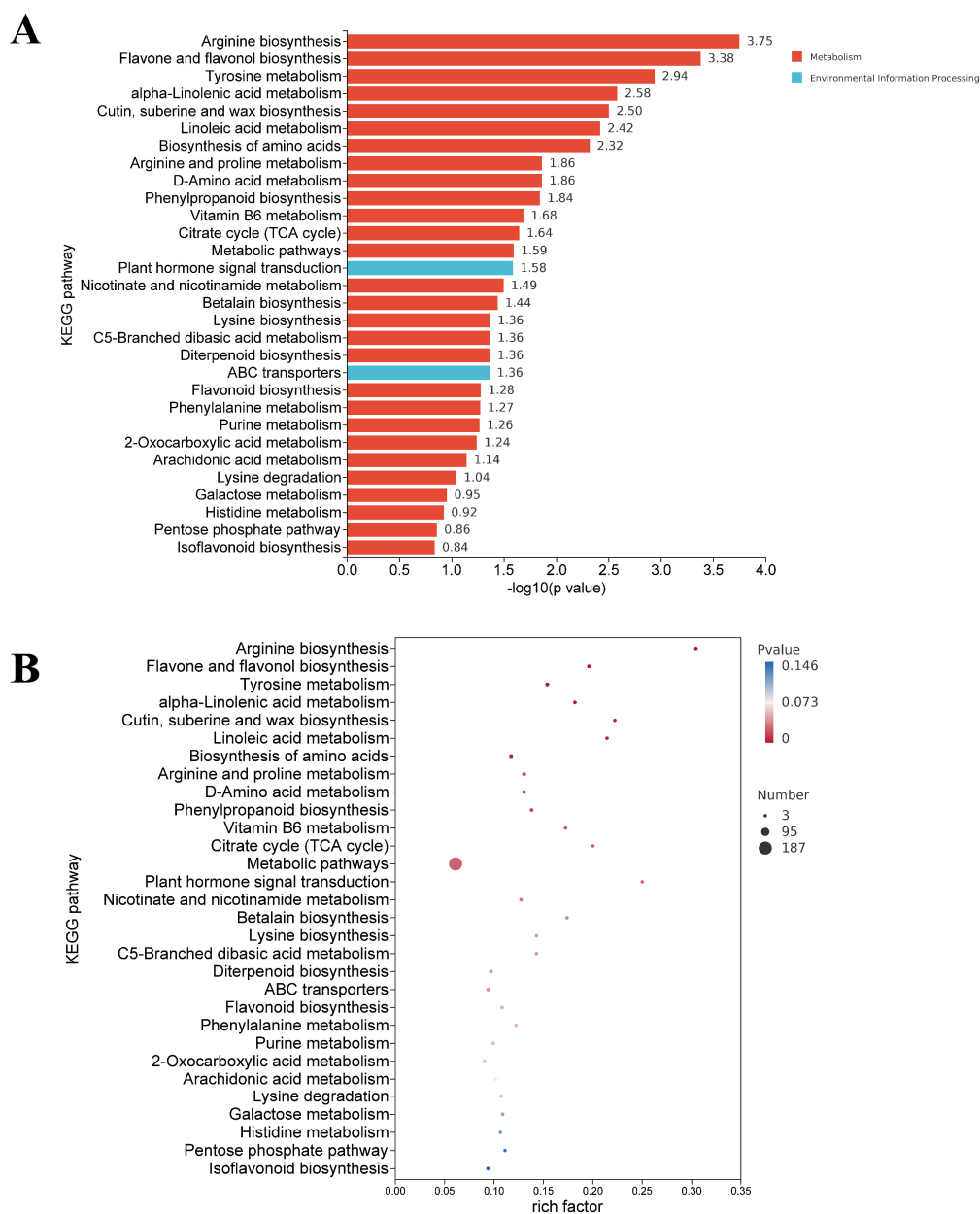

**Figure S14** (A) KEGG enrichment bar plot of HB VS HB-3; (B) KEGG enrichment factor plot of HB VS HB-3.

Supplement: Supplementary file 1 [file foods-15-01396-s001.zip › Figure S14.pdf]

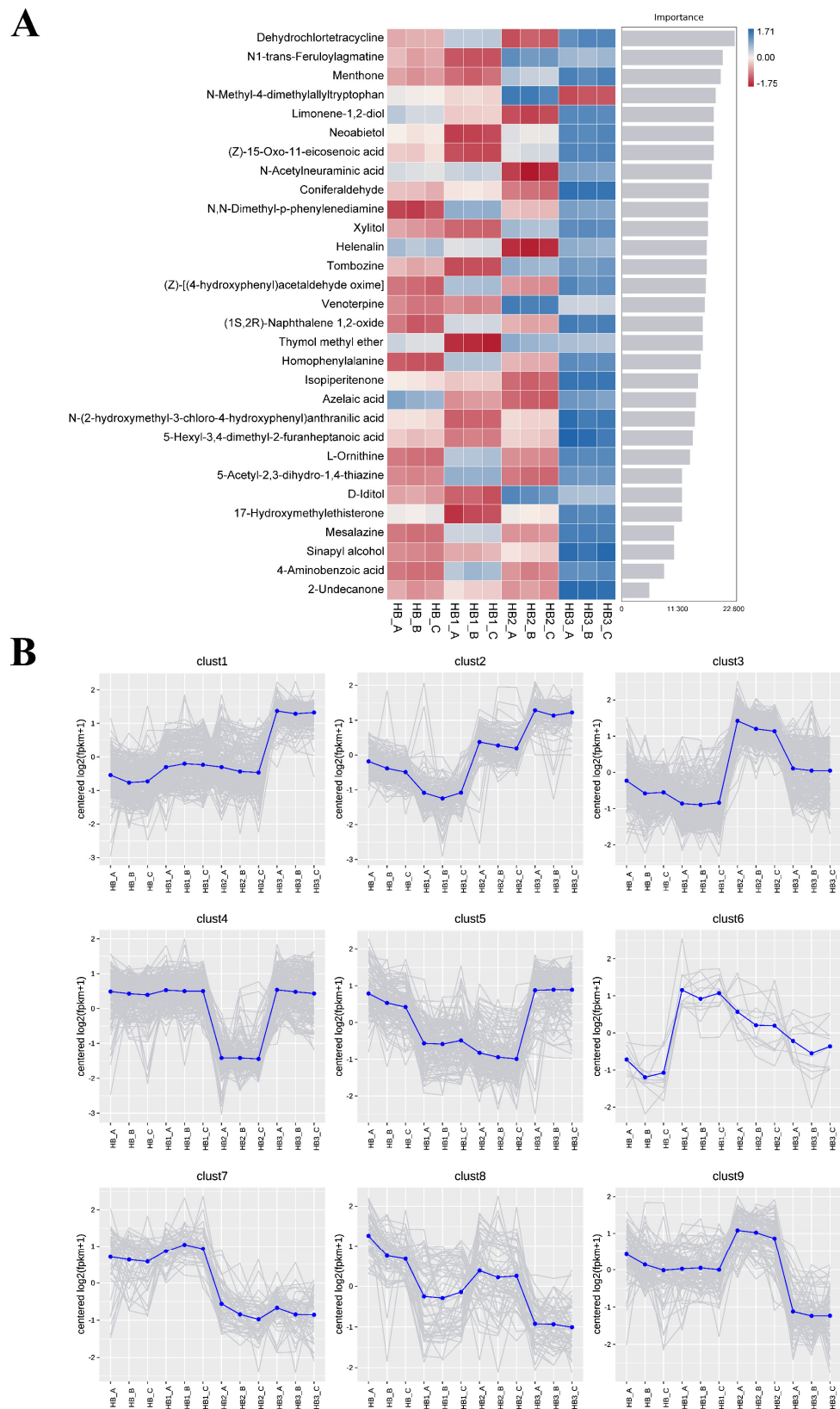

**Figure S16** (A) Random Forest plot among HB, HB-1, HB-2, and HB-3; (B) Differential metabolites trend analysis.

Supplement: Supplementary file 1 [file foods-15-01396-s001.zip › Figure S16.pdf]

A

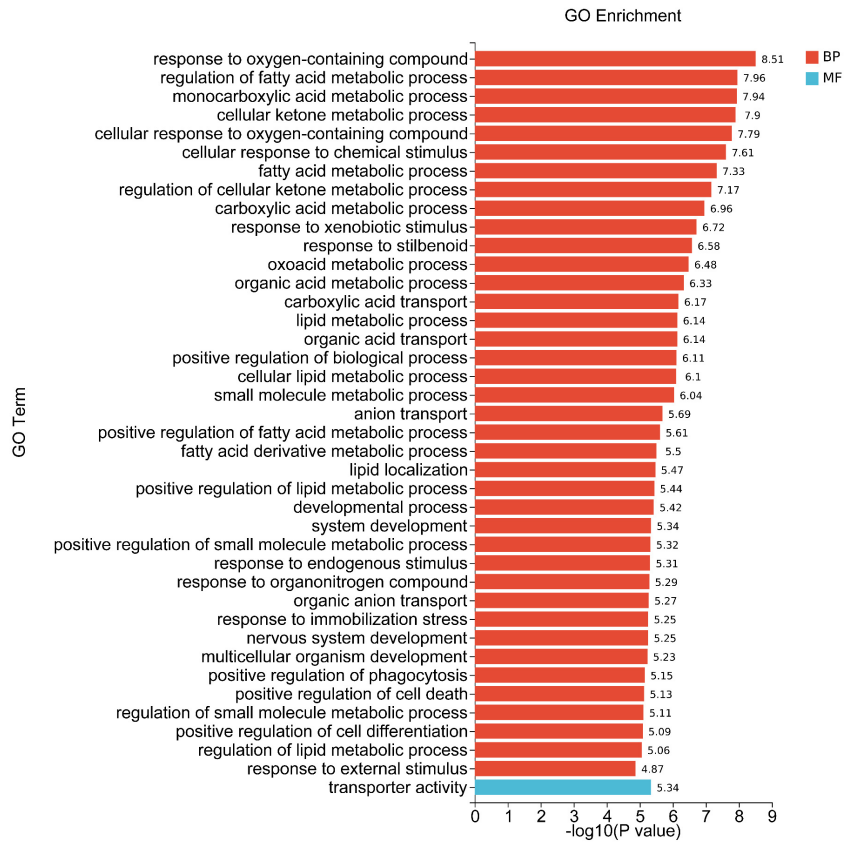

B

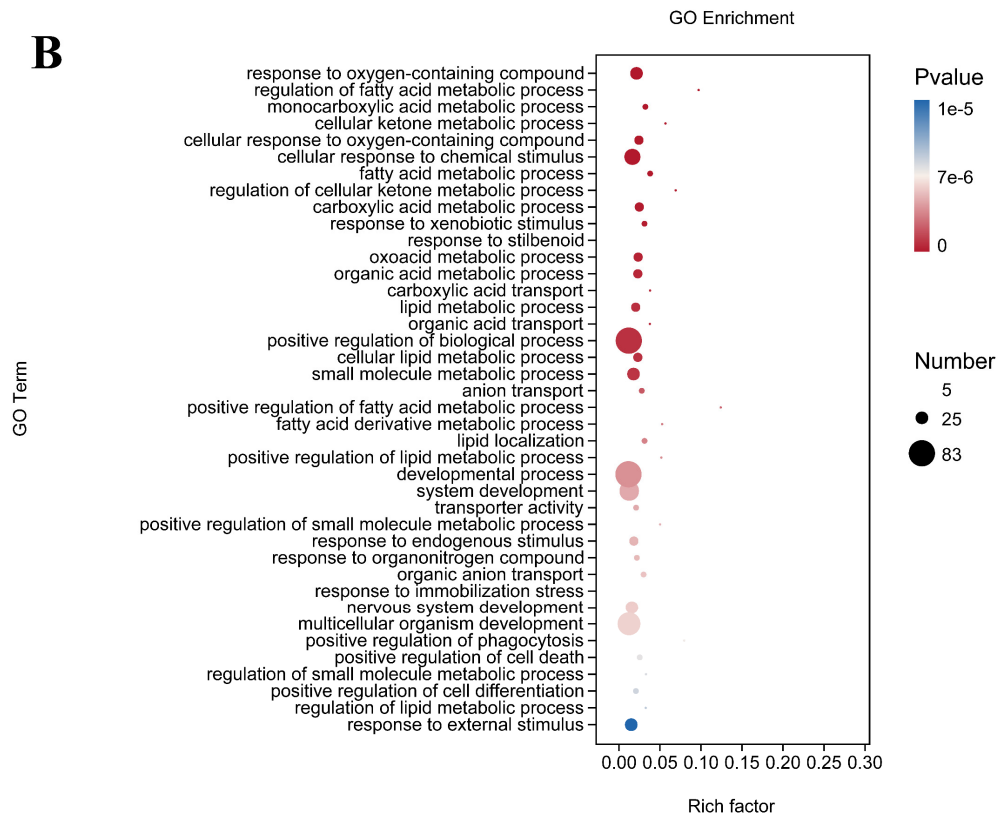

Figure S19 GO enrichment pathways analysis between HFCD and HFCD+HB-1.

Supplement: Supplementary file 1 [file foods-15-01396-s001.zip › Figure S19.pdf]

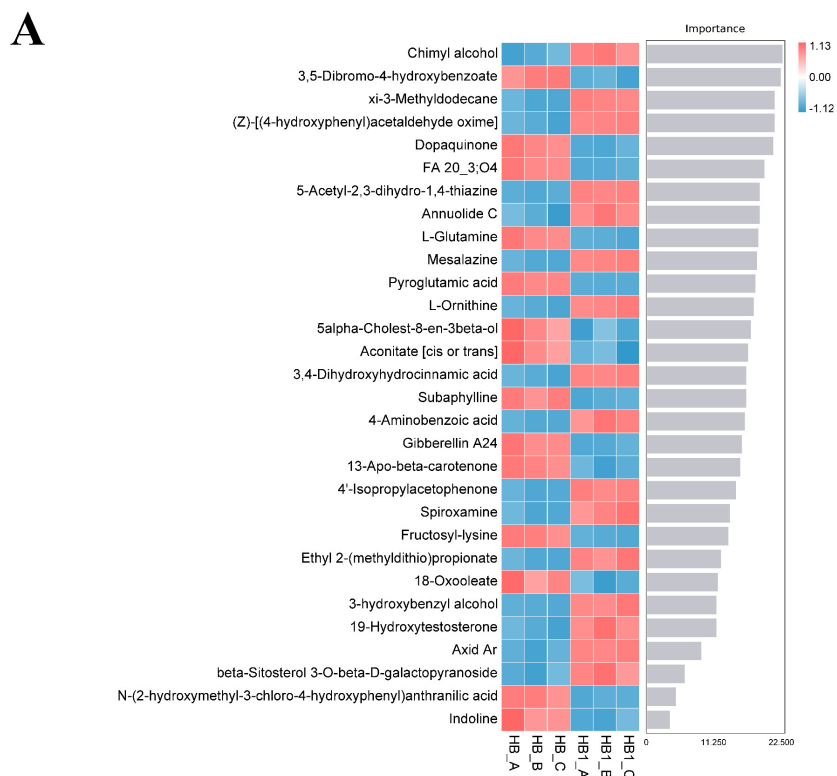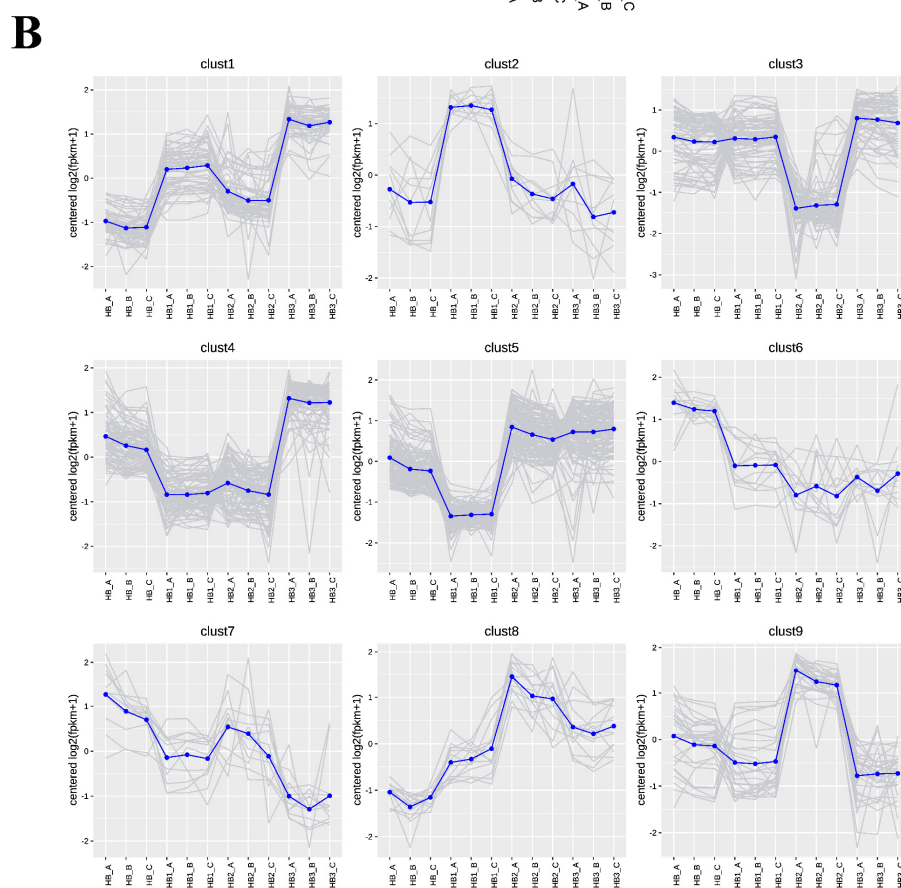

**Figure S9** (A) Random Forest plot of HB VS HB-1; (B) Differential metabolites trend analysis.

Supplement: Supplementary file 1 [file foods-15-01396-s001.zip › Figure S9.pdf]
